# A comprehensive benchmark for COVID-19 predictive modeling using electronic health records in intensive care

**DOI:** 10.1016/j.patter.2024.100951

**Published:** 2024-03-07

**Authors:** Junyi Gao, Yinghao Zhu, Wenqing Wang, Zixiang Wang, Guiying Dong, Wen Tang, Hao Wang, Yasha Wang, Ewen M. Harrison, Liantao Ma

**Affiliations:** 1 Peking University, Beijing 100871, China; 2 Centre for Medical Informatics, University of Edinburgh, EH16 4UX Edinburgh, UK; 3 Health Data Research UK, NW1 2BE London, UK; 4 Peking University People’s Hospital, Beijing 100044, China; 5 Peking University Third Hospital, Beijing 100191, China

**Keywords:** electronic health record, EHR, COVID-19, benchmark, deep learning, intensive care unit, ICU, mortality prediction, length-of-stay prediction

## Abstract

The COVID-19 pandemic highlighted the need for predictive deep-learning models in health care. However, practical prediction task design, fair comparison, and model selection for clinical applications remain a challenge. To address this, we introduce and evaluate two new prediction tasks—outcome-specific length-of-stay and early-mortality prediction for COVID-19 patients in intensive care—which better reflect clinical realities. We developed evaluation metrics, model adaptation designs, and open-source data preprocessing pipelines for these tasks while also evaluating 18 predictive models, including clinical scoring methods and traditional machine-learning, basic deep-learning, and advanced deep-learning models, tailored for electronic health record (EHR) data. Benchmarking results from two real-world COVID-19 EHR datasets are provided, and all results and trained models have been released on an online platform for use by clinicians and researchers. Our efforts contribute to the advancement of deep-learning and machine-learning research in pandemic predictive modeling.

## Highlights

• Introduced two predictive tasks for COVID-19 ICU care
• Benchmarking 18 models with real-world data for clinical use
• Open-source data preprocessing framework and online access to models and results

## The bigger picture

Predictive learning models aim to understand data and retrieve information about future outcomes. In medical settings, these models have enormous potential to predict relevant information, for example, the length of hospital stays, that can in turn help medical providers establish adequate treatment and patient care strategies. The COVID-19 pandemic revealed the importance of applying predictive models in health care. Studies evaluating multiple models and tasks that reflect the reality of clinical settings with real-world health data are critical for developing better and more precise frameworks for research in patient-level pandemic predictive modeling.

## Graphical abstract

**Figure:**
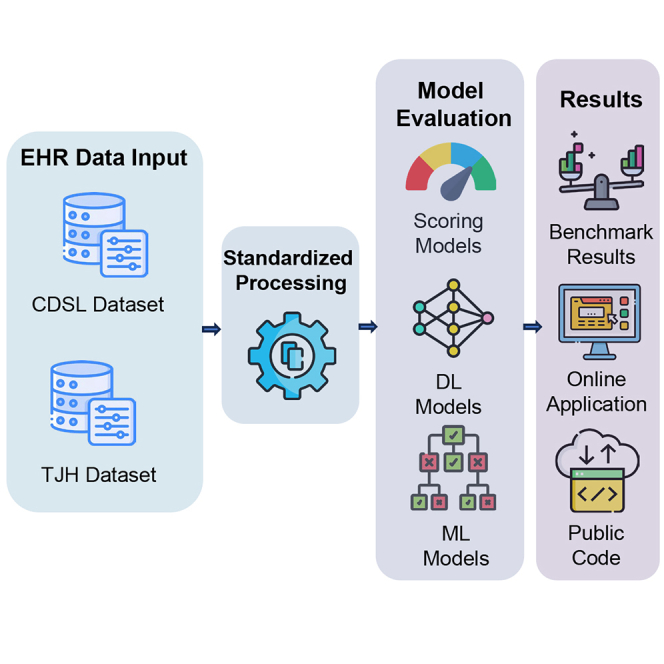

## Teaser

In this study, researchers tackle the challenges of predicting health-care outcomes during pandemics like COVID-19. They introduce innovative prediction tasks specifically for intensive care patients, evaluating a wide range of models from traditional to advanced deep learning. The study not only benchmarks these models using real-world data but also makes the results and tools publicly available. This work is significant, as it advances the use of deep learning in health care, offering valuable resources for both clinicians and researchers.

## Introduction

The COVID-19 pandemic has undoubtedly left an indelible impact on the global community. Although recent studies suggest that new COVID-19 variants are less lethal, their heightened transmissibility contributes to a continual surge in cases worldwide.^1^ Given these circumstances, the critical need for early risk prediction and disease progression estimation, especially for COVID-19 patients in intensive care units (ICUs), is essential to optimally allocate medical resources and alleviate the strain on our health-care system in the post-pandemic era.

Despite the commendable performance of existing COVID-19 predictive modeling works on specific datasets,^2^^,^^3^^,^^4^^,^^5^^,^^6^^,^^7^^,^^8^^,^^9^^,^^10^ researchers and clinicians often encounter difficulties when attempting to apply state-of-the-art prediction models to new data. Questions arise regarding whether to employ deep-learning (DL) models or traditional machine-learning (ML) models and how different models compare in terms of prediction performance. Although there are a few descriptive review works,^11^^,^^12^ there is still a lack of a fair quantitative comparative framework for existing models. Various models have been applied to different datasets, many of which either are not publicly available or entail strict access restrictions. Direct comparison of their results is therefore problematic, and model selection for new data is equally challenging, as re-implementing all existing models on new data is resource intensive, particularly for clinicians. This limitation curtails the practical usage of these models and hinders further research, underlining the necessity for a benchmark for COVID-19 predictive modeling that facilitates model comparison using identical data and evaluation strategies.

Existing electronic health record (EHR) prediction benchmarks^13^^,^^14^^,^^15^^,^^16^ predominantly rely on publicly available ICU datasets, such as MIMIC-III^17^ and MIMIC-II.^18^ These benchmarks compare various machine-learning and deep-learning models across multiple standardized prediction tasks, including mortality prediction, patient phenotyping, length-of-stay (LOS) prediction, etc. However, these efforts primarily evaluate the performance of basic machine-learning and deep-learning models like recurrent neural networks (RNNs) or long-short-term memory networks (LSTM), despite the presence of numerous advanced deep-learning models specifically designed for EHR data and clinical tasks. Notably, comprehensive benchmarking results for machine-learning, deep-learning, and more advanced models on publicly available datasets remain absent for COVID-19 predictive tasks.

Another concern lies in the fact that current COVID-19 prediction works largely mirror the task settings of previous EHR data-mining endeavors. Risk or mortality prediction models, for instance, typically output the predicted risk score at the final time step of available EHR sequences.^2^^,^^10^ This methodology can be problematic as it may be too late to initiate life-saving treatments for high-risk COVID-19 patients (especially ICU patients) by the time their status at the last time step is identified as critical.^19^^,^^20^ Simultaneously, the LOS prediction task is commonly formulated as a regression task,^15^ with the model outputting the predicted remaining LOS at each time step. This approach presents a notable issue where high-risk and low-risk patients may exhibit the same LOS value, as both death and ICU discharge are defined as the end of the stay in previous benchmarking works.^14^^,^^15^^,^^21^^,^^22^ Although the model can theoretically learn non-linearity and map these statuses to distinct locations in the latent embedding space, the practical application may prove challenging for clinicians. Adaptations are thus required for both tasks to cater to the unique intensive care setting associated with COVID-19. Consequently, it is imperative to compare existing deep-learning and machine-learning models using more clinically practical prediction tasks, identical publicly available data, and consistent evaluation settings.

In this study, we aim to bridge these gaps by proposing a standardized and comprehensive benchmark for COVID-19 prediction tasks in ICUs. This benchmark enables comparison among machine-learning, basic deep-learning, and state-of-the-art EHR-specific models. Our contributions are 3-fold:

(1) Task, metric, and model designing. We introduce two tasks based on the clinical practice for COVID-19 patients in ICUs: outcome-specific LOS prediction and early-mortality prediction*.* Distinct from existing LOS-prediction frameworks, the outcome-specific LOS prediction is formulated as a multitarget task. This task simultaneously outputs the patient outcome and the corresponding LOS, enabling clinicians to distinguish progression status between low-risk and high-risk patients. The early-mortality prediction task is designed to alert clinicians to high-risk patients as early as possible, thereby preempting potential treatment delays. We design specific evaluation metrics for both tasks, namely the early-prediction score (ES) and the outcome-specific mean absolute error (OSMAE), to assess model performance. We have also designed a multitask training architecture for the LOS-prediction task and a time-aware (TA) loss term that can significantly improve deep-learning models’ early-prediction performance.
(2) Data preparation and preprocessing pipelines. We have established data preprocessing pipelines that include cleaning, filtering, missing-value imputation, and cohort construction for two real-world COVID-19 EHR datasets. Both datasets consist of heterogeneous longitudinal EHR data from ICUs. Features include lab tests, vital signs, diagnoses, and static demographic information.
(3) Modeling and benchmarking. We have implemented and evaluated 18 state-of-the-art predictive models across the two tasks, including one clinical scoring method, five machine-learning models, six basic deep-learning models, and six deep-learning predictive models specifically designed for multivariate time-series EHR data. We conducted fair and reproducible comparisons and provide detailed benchmarking results to foster further research in this field.

We have made our code publicly available, enabling others to build complete benchmarks and reproduce all results. Our well-structured data preprocessing and modeling modules can also be easily applied to generate customized tasks and results. The benchmark code and documentations can be accessed at Zenodo: https://doi.org/10.5281/zenodo.10573567.^23^ Moreover, we have released all the benchmark experiment results and trained models on an online platform, which includes model performances with all hyperparameter combinations for both tasks and makes the results easy to query and download. The platform can be accessed at https://pyehr.netlify.app.^24^.

## Results

### Dataset description and problem formulation

The datasets used in this study and the proposed prediction tasks are as follows.

#### EHR datasets for COVID-19 patients in intensive care

In this work, we utilize two COVID-19 EHR datasets to conduct benchmark experiments. After performing an exhaustive search for publicly available COVID-19 EHR datasets worldwide, we selected two datasets based on ease of access and absence of regional restrictions:

Dataset 1 (Tongji Hospital COVID-19 dataset [TJH])^2^ comprises anonymized EHR data from 485 COVID-19 patients admitted to Tongji Hospital, China, between January 10 and February 24, 2020. The dataset includes 74 lab tests and vital signs, all of which are numerical features, as well as two demographic features (age and gender). This dataset is publicly accessible via GitHub (https://github.com/HAIRLAB/Pre_Surv_COVID_19). The dataset download script is included in our benchmark code.

Dataset 2 (HM Hospitales COVID Data Save Lives [CDSL])^25^ originates from the HM Hospitales EHR system and contains anonymized records of 4,479 patients admitted with a diagnosis of COVID-19 or suspected COVID-19 infection. The dataset includes heterogeneous medical features such as detailed information on diagnoses, treatments, admissions, ICU admissions, diagnostic imaging tests, laboratory results, and patient discharge or death status. The dataset is open to global researchers and can be accessed upon request (https://www.hmhospitales.com/prensa/notas-de-prensa/comunicado-covid-data-save-lives). Prospective users must complete a request form.

These datasets have been utilized in several mortality prediction studies.^2^^,^^26^ However, we found no existing well-organized data preprocessing pipeline code or benchmarking results for them, which prompted us to provide a comprehensive benchmark analysis and data-processing suite for these publicly available datasets, thereby facilitating related research.

#### Problem formulation and evaluation metrics

In this study, we formulate two tasks—outcome-specific LOS prediction and early-mortality prediction—to evaluate and compare the performances of various machine-learning and deep-learning models. These tasks, which are adaptations of common LOS- and mortality-prediction models, are tailored to suit the requirements of COVID-19 intensive care settings.

##### Problem 1 (outcome-specific length-of-stay prediction)

LOS prediction is an important task in clinical practice, which better facilitates clinical resource management. We define outcome-specific LOS prediction as a multitarget prediction task, which encompasses a binary outcome classification task and an LOS regression task. At each time step *t*, the model generates two outputs: a predicted outcome ${\hat{y}}_{m,t}\in \left\{0,1\right\}$ (i.e., 1 for mortality and 0 for survival) and a predicted LOS ${\hat{y}}_{l,t}\geq 0$ indicating the remaining days corresponding to the predicted outcome or the end of the ICU stay. Compared with traditional LOS-prediction models that output only ${\hat{y}}_{l,t}$, our model provides a more comprehensive evaluation of a patient’s progression, allowing clinicians to differentiate LOS according to different outcomes. The model’s learning process may also benefit from this setting, as patients with varying health statuses are explicitly modeled in the latent space.

We use mean absolute error (MAE) and mean squared error (MSE) as evaluation metrics. Furthermore, to evaluate this multitarget task comprehensively, we propose a new metric, OSMAE, formulated as follows:

$$OSMAE=\left\{ \begin{array}{cc} \epsilon \left(\max\left(E-{\hat{y}}_{l},0\right)+\max\left(E-y_{l},0\right)\right) & ,{\hat{y}}_{m}\neq y_{m}\\ \epsilon \left|{\hat{y}}_{l}-y_{l}\right| & ,{\hat{y}}_{m}=y_{m} \end{array}\right.$$

$$\epsilon =\left\{ \begin{array}{cc} \frac{t}{E-L+\gamma }, & 0<t\leq L-\gamma \\ 1, & t>L-\gamma \end{array}\right.\text{.}$$

**Figure 1:**
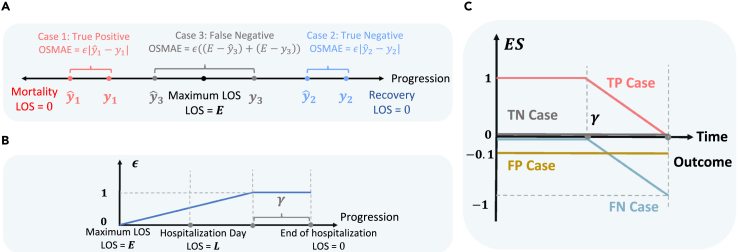
Illustrations of the proposed metrics (A) Illustration of the outcome-specific mean absolute error (OSMAE). (B) Illustration of the penalty term ϵ. (C) Illustration of the early-prediction score (ES).

Here, *t* represents the time step, *L* is the total LOS, and *E* is the maximum value at the 95th percentile of the LOS of all patients. ϵ is a penalty term represented by a piecewise function. A lower OSMAE indicates better model performance. Excluding the penalty term ϵ, OSMAE is equivalent to the conventional MAE for true-positive and true-negative predictions, i.e., ${\hat{y}}_{m}=y_{m}$. However, for false-positive and false-negative patients, OSMAE is significantly higher than the original MAE, since the model’s prediction for the patient’s outcome deviates completely from the actual outcome. We introduce the penalty term to prevent the model from incurring a high OSMAE during the early time steps, as a patient’s status may be uncertain during the initial ICU-stay phase. γ serves as a penalty threshold, indicating that we expect the model to make accurate predictions within the final γ days. The default value of γ is $0.5*{\bar{y}}_{l,0}$, where ${\bar{y}}_{l,0}$ is the average value of all patients’ total LOS. We provide a sensitivity analysis for γ in the discussion. Figure 1 illustrates the calculation of OSMAE and the penalty term using three examples: true positive, false negative, and true negative.

##### Problem 2 (early-mortality prediction)

Early-mortality prediction is defined as a binary sequential prediction task. For each time step in the longitudinal EHR input sequence, the model will predict the mortality risk ${\hat{y}}_{r}\in \left\{0,1\right\}$ for this ICU stay, signifying whether the patient will succumb by the end of the ICU stay. An optimal early-prediction model should raise an alert as soon as possible during the stay. Many existing mortality-prediction models feed the entire EHR sequence into the model and predict the mortality risk within a short future time window (e.g., 24 or 12 h).^15^^,^^16^^,^^27^^,^^28^ However, this approach restricts the clinical applicability of these models. Particularly for COVID-19 patients in the ICU, their health status may have severely deteriorated by the final time step, making the risks evident to clinicians. At this advanced stage, an alarm indicating high risk may be too late to initiate life-saving treatments or procedures. To address this limitation, we propose to assess early-prediction performance using weighted metrics.

To evaluate models’ early-prediction performance, we not only utilize traditional measures such as area under the receiver operating characteristic (AUROC) and area under the precision-recall curve (AUPRC), but we also introduce the early-prediction score (ES) to specifically assess the early-prediction performance of the models. Figure 1C illustrates the ES. Drawing from previous studies,^20^ the idea is to assign a full score to early true-positive predictions while penalizing late false-negative predictions, i.e., the model fails to predict patients’ outcomes accurately even in the final moments. We also give false alarms a small penalty (−0.1). Different penalty scores will not affect the relative comparison between different models. We provide details in the discussion. We normalize the total ES for one patient as follows:

$$ES_{normalized}=\max\;\left(ES_{total}/ES_{optimal},-1\right)\text{.}$$

This normalization ensures that the ground truth (i.e., the highest possible score) receives a normalized score of 1 and the worst algorithm, which outputs only negative predictions, receives a normalized score of −1.

### Pipeline design

In this section, we introduce our benchmarking pipeline design, including data preprocessing, baseline selection, and training and evaluation strategies.

#### Data preprocessing

Our benchmark data processing pipeline comprises four stages: data cleaning, merging, normalization, and imputation. Although both datasets are well-formatted data tables, they still contain artifacts that necessitate preprocessing. Previous machine-learning and deep-learning works on COVID-19 predictive modeling lack a uniform setting for these preprocessing details, leading to subtle performance differences. In this study, we propose a reproducible and reliable preprocessing pipeline to establish a fair comparison base for various models.

#### Data cleaning and merging

Our first step in preprocessing the dataset into feature matrices is to structure the EHR data format for use in machine-learning or deep-learning models. Each patient’s longitudinal EHR records are represented as a matrix, with each column representing a specific feature or label and each row representing a record. We also extract demographic information for each patient as static data. During this step, we calculate ground-truth labels, such as LOS and mortality outcome.

For error-record cleaning, we first compute each feature’s statistics, including the mean, standard deviation, minimum, maximum, median, and missing rate. For instance, in the TJH dataset, we observed that all entries for “2019-nCoV nucleic acid detection” are identical, and some other feature values are negative. We removed these clear error records. For example, in the CDSL dataset, we removed obvious errors such as oxygen saturation values above 100 and maximum blood pressure exceeding 220. All filtered values are replaced with NaN (not a number). Detailed feature statistics can be found in supplemental experimental procedures 9.

The raw datasets have record-level missing rates of 84.25% for the TJH dataset and 98.38% for the CDSL dataset. High missing rates may impair models’ prediction performance. Hence, following the previous benchmark preprocessing settings,^15^ we merged data into day level for the TJH dataset and hour level for the CDSL dataset, which reduces the missing rate of recorded physiological characteristics. It is worth noting that for the day/hour that does not have any record, we do not duplicate records from previous time steps. This data merging will not result in significant information loss since most records (over 95%) do not record different values for the same feature on the same day or at the same hour. If there were multiple records for the same feature in the same time slot, we recorded their mean value. For the CDSL dataset, the feature dimensions are much larger and features are sparser than in the TJH dataset: nearly 85% of the features have a missing rate beyond 90%, signifying that over 90% of patients never have records for these features. Therefore, we removed features with a missing rate higher than 90% among all patients.

#### Data normalization and imputation

We applied *Z* score normalization to all demographic features, vital signs, lab test features, and LOSs. Here, we calculated the mean and standard deviation based on the data in the 5%–95% quantile range. Preventing future information leakage is crucial for maintaining the fairness of a benchmark. To address this, we implemented stringent strategies: (1) for missing values, we employed a forward-filling imputation method. Specifically, we replaced missing values with the most recent ones. If a patient lacked a prior record for a specific feature, we filled the missing data with the median value from all patients in the training set. (2) We utilized the means and standard deviations from the training set to normalize the entire dataset. This practice ensures no leakage regarding test set data distribution, a detail often overlooked in many previous benchmarking studies.

The data statistics of the two processed datasets are shown in Tables 1 and 2.

**Table 1:** Statistics of the TJH dataset

Mortality outcome	Total	Alive	Dead
No. of patients	361	195 (54.02%)	166 (45.98%)
No. of records	1,704	1,050 (61.62%)	654 (38.38%)
Average number of records	5.0 [3, 6]	5.0 [4, 7]	3.0 [2, 5]
Age (years)	62.0 [46.0, 70.0]	51.0 [37.0, 62.0]	69.0 [62.25, 77.0]
>Average (58)	205 (56.79%)	68 (34.87%)	137 (82.53%)
≤Average (58)	156 (43.21%)	127 (65.13%)	29 (17.47%)
Gender	58.7% male	47.2% male	72.3% male
Male	212 (58.73%)	92 (47.18%)	120 (72.29%)
Female	149 (41.27%)	103 (52.82%)	46 (27.71%)
No. of features	75	–	–
Length of stay (days)	10.0 [4.0, 15.0]	14.0 [9.0, 17.0]	5.0 [3.0, 10.0]

The reported statistics are of the form median [Q1, Q3].

**Table 2:** Statistics of the CDSL dataset

Mortality outcome	Total	Alive	Dead
No of. patients	4,255	3,715 (87.31%)	540 (12.69%)
No. of records	123,044	108,142 (87.89%)	14,902 (12.11%)
Average no. of records	24.0 [15, 39]	25.0 [15, 39]	22.5 [11, 37]
Age (years)	67.2 [56.0, 80.0]	65.1 [54.0, 77.0]	81.6 [75.0, 89.0]
>Average (67)	2,228 (52.36%)	1,748 (47.05%)	480 (88.89%)
≤Average (58)	2,027 (47.64%)	1,967 (52.95%)	60 (11.11%)
Gender	59.1% male	58.5% male	63.3% male
Male	2,515 (59.11%)	2,173 (58.49%)	342 (63.33%)
Female	1,740 (40.89%)	1,542 (41.51%)	198 (36.67%)
No. of features	99	–	–
Length of stay (days)	6.4 [4.0, 11.0]	6.1 [4.0, 11.0]	6.0 [3.0, 10.0]

The reported statistics are of the form median [Q1, Q3].

### Benchmarking experiment settings

To provide a comprehensive benchmark comparison between existing models, we categorized the baseline models into four categories: clinical scoring models, machine-learning models, basic deep-learning models, and EHR-specific predictive models. Machine-learning and basic deep-learning models are popular for general classification and regression tasks. EHR-specific predictive models are designed explicitly for clinical predictive tasks with EHR data. We provide a list of baseline models below, and detailed descriptions of these models can be found in supplemental experimental procedures 1.

(1) Clinical scoring model: 4C mortality score.^29^
(2) Machine-learning models: decision tree (DT), random forest (RF), gradient boosting decision tree (GBDT), XGBoost,^30^ CatBoost.^31^
(3) Basic deep-learning models: multilayer perceptron (MLP), RNN,^32^ LSTM,^33^ gated recurrent units (GRU),^34^ temporal convolutional network (TCN),^35^ Transformer.^36^
(4) EHR-specific predictive models: RETAIN,^37^ StageNet,^38^ Dr. Agent,^22^ AdaCare,^27^ ConCare,^28^ GRASP.^39^

**Figure 2:**
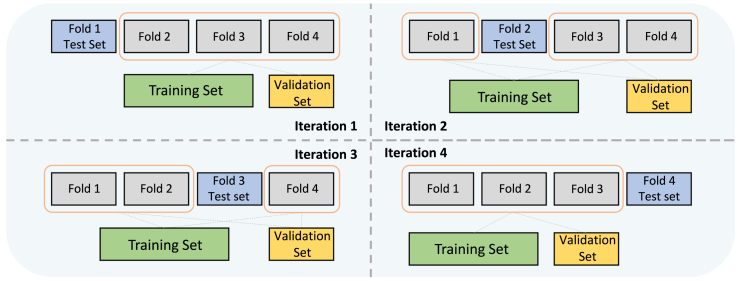
The K-fold cross-validation strategy We take 4-fold as an example. We use a stratified shuffle split to ensure that the proportions of alive and dead patients on all folds are the same as in the total cohort.

Machine-learning models and some deep-learning models (e.g., MLP) cannot handle sequential data as input. When training these models, we use the feature values at the current time step as input and predict the target. The clinical scoring method is applicable only to the mortality-prediction task. To minimize the randomness of the test-sample selection process, we employed the stratified 10-fold cross-validation strategy to train and evaluate the models. We randomly divided patients into 10 groups or “folds.” Then, we repeated the training and evaluation process 10 times. In the *i*-th iteration, we selected the *i*-th fold as the test set and divided the remaining folds into training and validation sets at an 8:1 ratio. An illustration of this process is provided in Figure 2. Finally, we report the means and standard deviations of the model performances on all test sets from the 10 iterations. Moreover, to evaluate the model’s generalizability over time more comprehensively, we have also conducted a standard holdout experiment. We divided the dataset into training, validation, and test sets in a 7:1:2 ratio, based on admission time (using the latest 20% of patients as the holdout dataset). We retrained all models five times with different random seeds and report the standard deviations. The results for the holdout performance are shown in supplemental experimental procedures 4.

Experiments are conducted on a server equipped with dual Intel Xeon Silver 4210R CPUs, each with 10 cores supporting 20 threads, two NVIDIA RTX 3090 GPUs, and 64 GB of RAM. Notably, all experiments are executed solely on one of the GPUs for consistent results. The model parameters and average training time are shown in Table 3.

**Table 3:** Model parameters and training time

Dataset	TJH		CDSL		
Model	Number of parameters	Runtime (1 epoch)	Number of parameters	Runtime (1 epoch)	
RF	–	1.71 s	–	26.69 s	
DT	–	1.30 s	–	8.10 s	
GBDT	–	4.04 s	–	128.55 s	
CatBoost	–	1.54 s	–	7.40 s	
XGBoost	–	1.50 s	–	9.40 s	
MLP	38.0k	1.91 s	39.5k	6.27 s	
RNN	13.9k	1.91 s	17.0k	6.10 s	
LSTM	41.0k	1.89 s	48.6k	6.30 s	
GRU	31.9k	1.85 s	38.1k	6.84 s	
TCN	55.8k	3.31 s	60.4k	31.60 s	
Transformer	73.0k	2.29 s	124k	17.37 s	
RETAIN	79.0k	2.38 s	135k	60.30 s	
StageNet	603k	2.31 s	622k	17.81 s	
Dr. Agent	41.9k	2.89 s	48.1k	15.81 s	
AdaCare	114k	3.05 s	130k	21.38 s	
GRASP	35.3k	2.41 s	39.9k	37.65 s	
ConCare	66.4k	2.59 s	89.1k	11.98 s	

Hyperparameters for the models are determined using a grid-search strategy on the validation set for each task. Hyperparameter settings can be found in supplemental experimental procedures 2, and the grid search results are available on our online platform. All deep-learning models are trained on the training set for 100 epochs, using an early stopping strategy. During each epoch, we assess the model’s performance on the validation set of each fold. After training, we load the model parameters that yield the best performance on the validation set and then evaluate the model on the test set. For the outcome prediction task, we select models based on the AUPRC score. For the LOS-prediction task, we use the MAE score as the criterion. All metrics are computed on a per-record basis.

### Model adjustments for the proposed tasks

This research additionally introduces model modifications tailored to the intended tasks in terms of both the loss function and the model architecture.

#### Outcome-specific length-of-stay prediction

**Figure 3:**
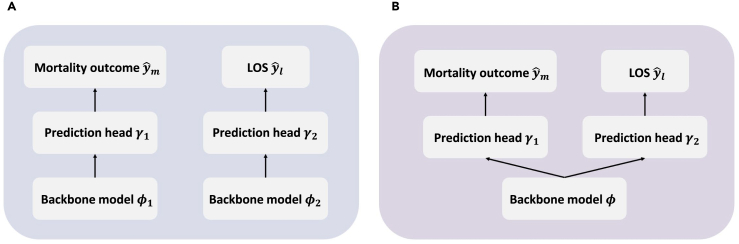
Illustrations of two training settings (A) The two-stage training setting. (B) The multitask training setting.

The task of outcome-specific LOS prediction is framed as a multitarget prediction task, for which we employ two distinct strategies. The first is end-to-end multitask learning, whereby a singular model backbone and two MLP prediction heads are trained, one for outcome prediction and the other for LOS prediction. The backbone and prediction heads are co-trained in an end-to-end fashion. The second approach is the two-stage training, in which two separate models, each with identical structures, are trained to predict the outcome and LOS independently. Depictions of these two training scenarios can be found in Figure 3. We include performance metrics for both approaches. It is important to note that traditional machine-learning models accommodate only two-stage settings; hence, we present only the performance metrics of the two-stage training approach for such models.

#### Early-mortality prediction

Given the nature of the early-mortality prediction task, we devised a straightforward yet potent loss function to guide deep-learning models about the decision time threshold. The suggested TA loss is formulated as follows:

$$L_{TA}=\frac{1}{T}\sum_{t}^{T}\left(L_{CE}*\;\exp\;\left(-\zeta l_{t}\right)\right)-\eta r$$

$$r=\frac{1}{T}\sum_{t}^{T}\left(l_{t}*\left|{\hat{y}}_{t}-y_{t}\right|\right)\text{.}$$

In this formula, $L_{CE}$ is the cross-entropy loss, $l_{t}$ is the time to outcome at the *t*-th time step, ζ denotes the decay rate, η denotes the reward factor, and *r* denotes the reward term. The second part confers rewards on earlier correct predictions and penalizes late incorrect predictions by either decreasing or increasing the loss values. The first part of the proposed loss function works to prevent early incorrect predictions from incurring excessive penalties through the application of an exponential decay function. This proposed TA loss function can be readily integrated into any deep-learning model, thereby improving early-mortality prediction performance. It should be noted that as the loss term is employed only during the training phase, the $l_{t}$ term is not required for the inference stage. Consequently, this ensures that there is no future information leakage at the inference stage.

In this section, we present comprehensive benchmarking results of all predictive models across all datasets and tasks. To assess the statistical significance of our model’s performance, we performed t tests on all results. The bootstrap T test^40^ was employed to calculate p values. We set the sample number for the bootstrap at 1,000 and the number of sampling processes at 50. The comparisons were made between different variations of the model, for instance, comparing the model with and without TA loss in the early-mortality prediction task and between two-stage and multitask settings in the LOS-prediction task. The p values are reported in supplemental experimental procedures 7.

### Benchmarking performance of outcome-specific length-of-stay prediction

The benchmarking performances for the task of outcome-specific LOS prediction, under both multitask and two-stage settings, are presented in Table 4. On the TJH dataset, the GRU model with two-stage learning achieves the lowest MAE and MSE. Dr. Agent with multitask learning demonstrates the lowest OSMAE. On the CDSL dataset, StageNet with two-stage learning achieves the lowest MAE and MSE, while the TCN model with multitask learning achieves the lowest OSMAE.

**Table 4:** Benchmarking performance of outcome-specific length-of-stay prediction

Dataset	TJH			CDSL			
Metric	MAE (↓)	MSE (↓)	OSMAE (↓)	MAE (↓)	MSE (↓)	OSMAE (↓)	
RF_t_	4.83 ± 0.53	40.94 ± 8.77	6.14 ± 0.99	4.05 ± 0.13	31.30 ± 4.42	4.90 ± 0.16	
DT_t_	5.07 ± 0.53	50.16 ± 12.06	7.02 ± 1.33	4.11 ± 0.15	32.23 ± 4.81	4.99 ± 0.14	
GBDT_t_	4.79 ± 0.53	42.03 ± 9.58	5.92 ± 0.82	4.01 ± 0.14	30.74 ± 4.55	4.82 ± 0.19	
CatBoost_t_	4.71 ± 0.53	39.72 ± 9.23	5.70 ± 0.85	4.01 ± 0.14	30.62 ± 4.61	4.79 ± 0.17	
XGBoost_t_	4.78 ± 0.55	41.76 ± 9.47	5.77 ± 0.92	4.02 ± 0.13	31.10 ± 4.42	4.81 ± 0.15	
MLP_t_	5.08 ± 0.48	47.21 ± 11.10	6.34 ± 0.97	4.05 ± 0.15	32.08 ± 4.96	4.80 ± 0.17	
MLP_m_	4.90 ± 0.52∗^#^	44.35 ± 14.00∗^#^	7.26 ± 2.81	4.08 ± 0.16	31.54 ± 4.53∗^#^	4.86 ± 0.21	
RNN_t_	4.57 ± 0.48	39.82 ± 10.02	5.54 ± 1.30	3.90 ± 0.22	30.66 ± 5.58	4.37 ± 0.26	
RNN_m_	4.68 ± 0.49	40.21 ± 9.29	5.46 ± 1.43∗^#^	3.87 ± 0.14∗^#^	29.52 ± 4.98∗^#^	4.43 ± 0.18	
LSTM_t_	4.49 ± 0.63	37.38 ± 12.64	4.88 ± 1.30	3.81 ± 0.16	29.29 ± 4.88	4.34 ± 0.23	
LSTM_m_	4.46 ± 0.60∗^#^	39.24 ± 10.30	5.53 ± 1.86	3.85 ± 0.13	30.13 ± 4.22	4.40 ± 0.19	
GRU_t_	4.33 ± 0.49^†^	35.10 ± 9.61^†^	5.16 ± 1.31	3.75 ± 0.18	28.90 ± 4.68	4.34 ± 0.32	
GRU_m_	4.80 ± 0.48	41.78 ± 9.49	5.38 ± 1.09	3.98 ± 0.18	32.14 ± 4.31	4.55 ± 0.22	
TCN_t_	4.69 ± 0.60	43.29 ± 13.27	5.77 ± 0.92	3.75 ± 0.17	29.48 ± 4.78	4.31 ± 0.15	
TCN_m_	4.79 ± 0.55	41.56 ± 9.73∗^#^	5.52 ± 0.85∗^#^	3.80 ± 0.16	29.45 ± 4.47∗^#^	4.22 ± 0.18∗^#†^	
Transformer_t_	5.04 ± 0.43	43.88 ± 7.22	5.96 ± 1.62	3.98 ± 0.20	32.35 ± 5.61	5.19 ± 0.19	
Transformer_m_	5.06 ± 0.46	46.02 ± 11.63	6.61 ± 1.57	4.00 ± 0.20	32.02 ± 4.68∗^#^	5.13 ± 0.19∗^#^	
RETAIN_t_	4.64 ± 0.61	41.22 ± 14.45	4.77 ± 1.18	3.83 ± 0.16	30.56 ± 5.99	4.41 ± 0.26	
RETAIN_m_	4.62 ± 0.55∗^#^	40.06 ± 10.34∗^#^	5.03 ± 0.96	3.84 ± 0.20	29.92 ± 4.48∗^#^	4.41 ± 0.30	
StageNet_t_	4.60 ± 0.76	41.70 ± 14.21	5.59 ± 1.33	3.72 ± 0.15^†^	28.81 ± 4.57^†^	4.33 ± 0.22	
StageNet_m_	4.49 ± 0.42∗^#^	39.55 ± 8.52∗^#^	7.10 ± 1.74	3.78 ± 0.18	29.93 ± 4.50	4.28 ± 0.19∗^#^	
Dr. Agent_t_	4.61 ± 0.58	40.85 ± 12.12	4.93 ± 1.14	3.80 ± 0.18	29.70 ± 4.96	4.46 ± 0.27	
Dr. Agent_m_	4.41 ± 0.58∗^#^	37.55 ± 11.38∗^#^	4.75 ± 1.11∗^#†^	3.82 ± 0.19	29.45 ± 4.70∗^#^	4.49 ± 0.28	
AdaCare_t_	4.55 ± 0.61	39.67 ± 11.35	5.08 ± 0.62	3.80 ± 0.15	29.07 ± 4.57	4.42 ± 0.15	
AdaCare_m_	4.41 ± 0.63∗^#^	38.86 ± 10.75∗^#^	5.24 ± 0.98	3.81 ± 0.16	32.05 ± 4.27	4.43 ± 0.18	
GRASP_t_	4.57 ± 0.43	40.65 ± 11.46	5.51 ± 1.13	3.84 ± 0.16	29.95 ± 5.06	4.43 ± 0.25	
GRASP_m_	4.44 ± 0.50∗^#^	37.79 ± 10.27∗^#^	5.31 ± 1.31∗^#^	3.89 ± 0.12	30.01 ± 4.33	4.49 ± 0.28	
ConCare_t_	4.69 ± 0.52	40.26 ± 11.13	5.29 ± 1.31	3.81 ± 0.17	30.16 ± 5.03	4.29 ± 0.23	
ConCare_m_	4.67 ± 0.56∗^#^	40.42 ± 10.77	5.27 ± 1.35∗^#^	3.85 ± 0.11	29.15 ± 3.90∗^#^	4.46 ± 0.24	

The reported score is in the form of mean ± SD. Subscript m signifies a multitask learning strategy, while subscript t indicates a two-stage learning strategy. ^†^The best performance. ^#^The multitask setting outperforms the two-stage learning strategy. ∗The performance improvement against the two-stage model is statistically significant (p < 0.05).

We observe that the multitask strategy generally outperforms the two-stage strategy on the TJH dataset. On the TJH dataset, we find that EHR-specific models can better benefit from multitask learning settings, suggesting that the multitask setting may better facilitate these models’ ability to map patients’ diverse statuses in the latent space. We visualize the learned embeddings in the latent space using t-distributed stochastic neighbor embedding (t-SNE) in supplemental experimental procedures 6. In addition, it is important to note the discrepancy between OSMAE and MAE. Despite both metrics assessing absolute error, the OSMAE values are notably larger than the MAE values on both datasets. On average, the OSMAE is 22% higher than the MAE on the TJH dataset and 17% higher on the CDSL dataset. This suggests that MAE might potentially distort the evaluation of model performance, as it cannot measure the error for false-negative and false-positive predictions, despite its widespread use in previous LOS-prediction studies. We also conduct the error analysis for the MAE in supplemental experimental procedures 8. In our current setting, we utilize two identical model structures for two-stage training. However, future work could potentially involve the use of distinct models for the two tasks. Further research could also focus on designing a more sophisticated multitask architecture or incorporating the principles of transfer learning.

### Benchmarking performance of early mortality prediction

The comparative performances of the models in early-mortality prediction are presented in Table 5. We also introduced the TA loss, labeling these adjusted models with a “TA” suffix for all deep-learning models. For the TJH dataset, the AdaCare-TA model delivered the highest AUPRC and AUROC, while Dr. Agent provided the highest ES. In contrast, for the CDSL dataset, ConCare-TA achieved the highest AUPRC, while StageNet-TA produced the highest AUROC and ES. Among traditional machine-learning and basic deep-learning models, CatBoost and GRU showed better performance on both datasets.

**Table 5:** Benchmarking performance on the task of early-mortality prediction

Dataset	TJH			CDSL		
Metric	AUPRC(↑)	AUROC(↑)	ES(↑)	AUPRC(↑)	AUROC(↑)	ES(↑)
4C	89.84 ± 4.46	94.16 ± 2.57	–	23.93 ± 2.99	76.15 ± 4.06	–
RF	95.56 ± 2.69	96.58 ± 2.20	72.63 ± 9.15	49.48 ± 3.79	84.35 ± 2.66	−10.54 ± 3.57
DT	80.48 ± 7.26	87.41 ± 3.99	67.86 ± 11.24	38.27 ± 5.21	79.67 ± 4.61	−8.23 ± 4.17
GBDT	95.13 ± 3.51	96.41 ± 2.35	76.45 ± 7.13	50.32 ± 5.15	85.15 ± 2.83	2.25 ± 4.86
CatBoost	95.99 ± 2.61	97.14 ± 1.81	74.91 ± 9.00	50.86 ± 4.34	85.09 ± 2.86	−3.94 ± 3.89
XGBoost	95.70 ± 2.98	96.84 ± 2.09	76.41 ± 9.90	49.70 ± 5.06	84.59 ± 3.09	−3.12 ± 4.44
MLP	93.78 ± 2.80	95.95 ± 1.62	72.87 ± 8.22	48.60 ± 3.57	84.15 ± 2.72	−0.76 ± 3.95
MLP-TA	93.21 ± 2.78	95.65 ± 1.85	73.94 ± 8.96^#^	48.67 ± 3.07^#^	84.24 ± 2.56^#^	0.55 ± 3.71^#^
RNN	96.03 ± 3.42	97.41 ± 2.19	78.33 ± 11.35	57.57 ± 6.55	87.81 ± 2.55	19.66 ± 8.86
RNN-TA	95.97 ± 3.65	97.38 ± 2.29	79.79 ± 11.06∗^#^	58.21 ± 6.34∗^#^	88.01 ± 2.36∗^#^	20.15 ± 11.25∗^#^
LSTM	94.82 ± 7.65	97.08 ± 3.65	84.80 ± 10.68	55.53 ± 8.54	87.02 ± 3.01	19.63 ± 11.41
LSTM-TA	95.40 ± 5.53∗^#^	97.10 ± 3.05∗^#^	83.78 ± 8.30	56.89 ± 7.34^#^	87.76 ± 2.50^#^	20.92 ± 13.10^#^
GRU	96.33 ± 3.18	97.59 ± 2.13	78.33 ± 15.45	56.78 ± 8.02	87.93 ± 2.89	21.66 ± 8.86
GRU-TA	96.50 ± 3.04^#^	97.70 ± 2.06^#^	80.93 ± 13.84∗^#^	57.75 ± 5.36∗^#^	87.98 ± 2.62∗^#^	23.54 ± 8.10∗^#^
TCN	93.13 ± 5.10	96.39 ± 1.89	74.66 ± 13.03	57.09 ± 5.84	87.40 ± 2.67	13.48 ± 12.04
TCN-TA	93.31 ± 4.97^#^	96.74 ± 1.87∗^#^	76.47 ± 12.27^#^	57.71 ± 5.72^#^	87.59 ± 2.60^#^	21.59 ± 12.68∗^#^
Transformer	93.47 ± 6.73	96.86 ± 2.13	79.21 ± 13.05	38.34 ± 5.07	81.32 ± 3.46	−3.96 ± 13.23
Transformer-TA	93.86 ± 6.42^#^	97.01 ± 2.01^#^	80.73 ± 12.47∗^#^	40.18 ± 5.00^#^	82.36 ± 3.61∗^#^	−0.78 ± 11.42∗^#^
RETAIN	96.49 ± 2.05	97.84 ± 1.44	82.59 ± 12.29	54.54 ± 7.97	85.02 ± 3.93	7.32 ± 10.67
RETAIN-TA	95.99 ± 2.42	97.82 ± 1.51	82.85 ± 11.36∗^#^	54.30 ± 6.50	85.43 ± 2.81^#^	9.00 ± 7.66^#^
StageNet	95.71 ± 3.77	97.27 ± 2.38	77.44 ± 12.52	56.57 ± 6.82	87.09 ± 3.54	23.93 ± 9.26
StageNet-TA	95.79 ± 3.86∗^#^	97.32 ± 2.41∗^#^	78.88 ± 12.29∗^#^	58.19 ± 6.48∗^#^	88.18 ± 2.82∗^#†^	24.55 ± 9.32^#†^
Dr. Agent	97.22 ± 2.44	98.00 ± 1.75	85.80 ± 7.97	54.17 ± 8.92	86.76 ± 3.64	17.53 ± 11.25
Dr. Agent-TA	97.16 ± 2.56	98.00 ± 1.97	86.01 ± 7.66^#†^	53.06 ± 7.34	87.06 ± 3.30∗^#^	19.35 ± 9.60∗^#^
AdaCare	97.86 ± 1.09	98.53 ± 0.77	78.39 ± 7.51	55.32 ± 4.45	86.59 ± 1.99	17.46 ± 8.66
AdaCare-TA	98.11 ± 1.13^#†^	98.64 ± 0.89^#†^	84.51 ± 8.17∗^#^	55.62 ± 5.44^#^	87.00 ± 2.18^#^	20.09 ± 9.86∗^#^
GRASP	96.04 ± 3.15	97.30 ± 2.30	81.63 ± 9.71	53.95 ± 8.37	84.44 ± 4.40	15.27 ± 15.17
GRASP-TA	96.16 ± 3.17^#^	97.40 ± 2.21^#^	82.82 ± 9.08^#^	53.50 ± 7.89	84.91 ± 3.73∗^#^	15.61 ± 13.76∗^#^
ConCare	97.01 ± 2.46	97.89 ± 1.62	80.97 ± 13.66	58.59 ± 6.49	87.76 ± 3.02	17.58 ± 12.34
ConCare-TA	97.04 ± 2.51^#^	97.94 ± 1.62^#^	82.31 ± 13.08∗^#^	58.68 ± 7.04^#†^	87.96 ± 3.23^#^	20.90 ± 10.43^#^

The reported score is of the form mean ± SD. “TA” denotes that the model trained with the time-aware loss. ^†^The best performance. ^#^The model with time-aware loss outperforms the original model. ∗The performance improvement against the model without the TA version is statistically significant (p < 0.05). All three metrics are multiplied by 100 for readability purposes.

The results indicate that the proposed TA loss term can enhance the performance of most models, particularly in terms of the ES. The average improvement of the ES is 1.93% for the TJH dataset and 33.58% for the CDSL dataset. This underscores that this simplistic loss term can effectively aid models in making correct early decisions. It is worth noting that our goal is to evaluate the direct effectiveness of the proposed TA loss term, so we did not specifically tune the hyperparameters of all TA models. So even under this adverse condition, most TA models can still outperform the original version. In addition, we observe that for the TJH dataset, the performance of all models was higher, especially for the ES, suggesting that the task is more straightforward on this dataset. Patients’ initial status was also more severe in the TJH dataset, resulting in a much higher ES than in the CDSL dataset. This might be attributable to the fact that the TJH data were collected during the initial months of the pandemic, when the virus was significantly more lethal. The performance of EHR-specific models generally surpasses that of basic deep-learning models, which in turn outperform traditional machine-learning models. This is to be expected, as most EHR-specific models are better equipped to utilize characteristics present in EHR data, such as disease progression, while compared with deep-learning models, traditional machine-learning models are unable to leverage temporal relationships in sequential data.

## Discussion

In this section, we further explore the models’ performance in terms of the proposed metrics ES and OSMAE. These metrics show the models’ performance on the early and outcome-specific perspectives of two tasks. We also discuss the limitations and future directions of this work.

### Analysis of early-prediction performance

**Figure 4:**
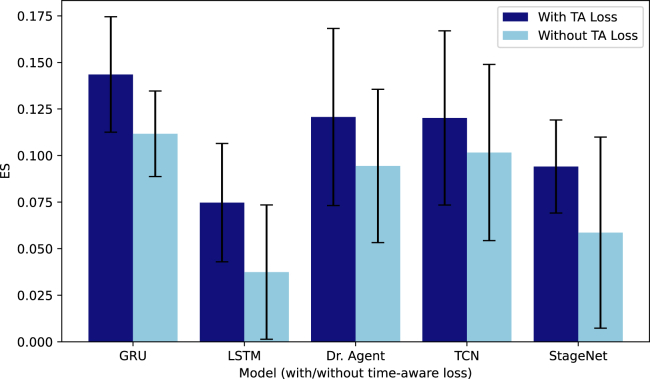
Early-prediction performance of five models with the highest ES on the CDSL dataset All models were trained using the first half of patient records. Error bars are standard deviations. All performance improvements are statistically significant (p < 0.05).

By enhancing early-prediction performance, models can discern patients’ health risks in the early stages. This can address the “cold-start” issue of predictive models, which is particularly beneficial during the early phases of a pandemic, when predictions must rely on sparse data. To assess the efficacy of our proposed early-prediction loss term in improving early-stage predictions, we simulated a scenario where the model generates forecasts using only the first half of a patient’s visit records. The ESs for the top five best-performing models (both with and without the TA loss) on the CDSL dataset are illustrated in Figure 4. The findings demonstrate that models incorporating the TA loss consistently achieve superior ESs in the early stages, which directly assesses the true-positive rate of predictions. This underscores the adaptability of our proposed TA loss in enhancing various models’ early-prediction capabilities.

**Figure 5:**
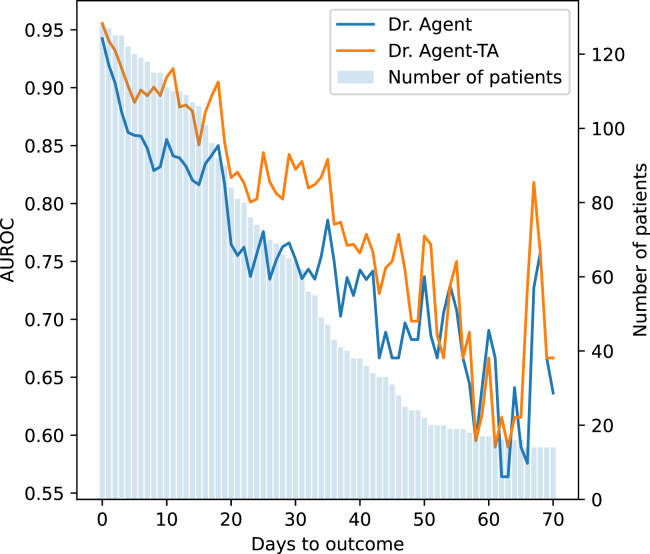
AUROC of Dr. Agent and Dr. Agent-TA at each visit We also plot the distribution of the number of patients on different days to outcome groups.

To offer a more granular view of the performance gains attributed to the TA loss, we have charted the AUROC at different visits (days to outcome) for the Dr. Agent model on the CDSL dataset as a case study in Figure 5. The figure shows that most patients’ LOS is less than 30 days, and the AUROC of the model with the TA loss is significantly higher. This suggests that, for most patients, the TA loss is more effective in identifying early health risks.

### Case study of prediction metrics

**Figure 6:**
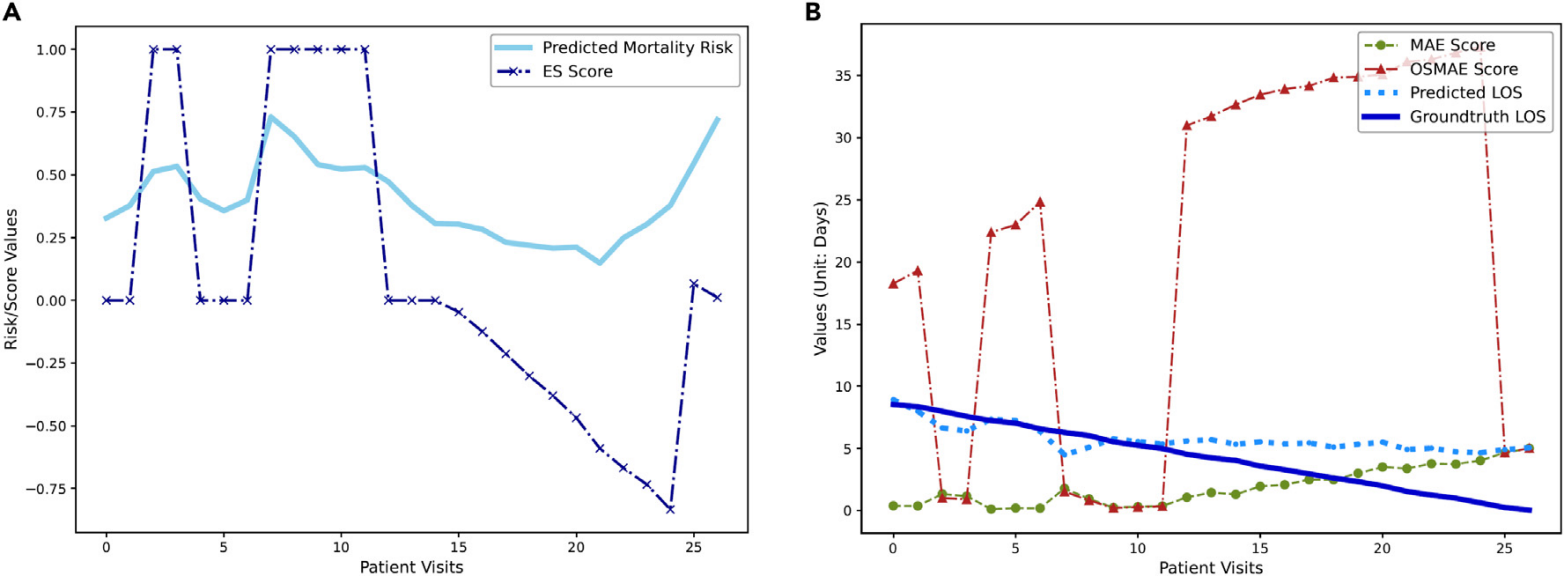
Case prediction plot on the CDSL dataset (A) ES and risk curve. (B) OSMAE, MAE, predicted LOS, and ground-truth LOS curve. We use the TCN model with the multitask setting to generate the prediction results.

The proposed ES and OSMAE metrics are inherently interpretable, as they are based solely on straightforward score calculations, independent of the models. This makes the calculation process transparent and easy to understand. To further elucidate our metrics, we have included a case study here, illustrated in Figure 6.

In Figure 6A, we plot the predicted risk probability over time alongside the ES at each time step for a patient whose actual outcome was mortality. Initially, the model accurately identifies early risk by predicting a risk probability above 0.5, earning a full ES (1.0) for these early correct predictions. However, over time, as the model incorrectly assesses the patient’s condition as improving, it receives progressively lower ESs. This decline reflects the model’s erroneous predictions as the patient approaches the final outcome. Near the end, even though the model predicts high risks again, the rewards for these correct predictions are lower due to their proximity to the final outcome.

In Figure 6B, we show the predicted LOS, the actual LOS, the MAE, and the OSMAE for the same patient. At first glance, the predicted LOS (dotted blue line) closely matches the actual LOS (solid blue line), resulting in a consistently low MAE (dotted green line). However, the higher OSMAE (red line) indicates that the model is making incorrect outcome predictions, suggesting a recovery when the patient’s condition is actually deteriorating. When outcomes are predicted accurately, OSMAE aligns with MAE. Using traditional MAE as a sole metric could lead to biased decisions, as it may not reflect the patient’s actual health trajectory.

This case study demonstrates that our proposed ES and OSMAE metrics can more accurately represent a model’s ability to assess early patient risk and understand disease progression, aspects often overlooked in traditional classification and regression metrics.

### Outcome-specific prediction performance

Another primary objective of our study is to determine how the proposed outcome-specific LOS-prediction task can enhance clinical decision-making. In Table 6, we display the prediction discrepancies for the top five models with the highest ΔMAE (OSMAE − MAE). To demonstrate how ΔMAE can retain information about the correctness of outcome predictions while computing the MAE, we also include the number and percentage of patients with incorrect outcome predictions.

**Table 6:** Prediction discrepancy of five models on the CDSL dataset

Model	Type	ΔMAE	Outcome wrong (n)	Outcome wrong (%)
Transformer	Two-stage	1.21	1,469	11.89%
Transformer	Multitask	1.13	1,389	11.24%
MLP	Multitask	0.78	1,322	10.70%
MLP	Two-stage	0.75	1,295	10.48%
Dr. Agent	Multitask	0.67	1,108	8.97%

The results indicate that as ΔMAE increases, the percentage of incorrect outcome predictions also rises. This suggests that an increase in outcome prediction error contributes to the discrepancy between our new OSMAE metric and the traditional MAE. This conclusion further implies that in traditional LOS-prediction tasks, relying solely on MAE to assess prediction accuracy can lead to erroneous conclusions and potentially compromise clinical decision-making. Consequently, refining the OSMAE metric—rather than solely concentrating on the conventional MAE—is crucial for future predictive modeling endeavors, possibly through a more judicious multitask learning framework or regularizer.

### Sensitivity analysis of OSMAE and ES

**Figure 7:**
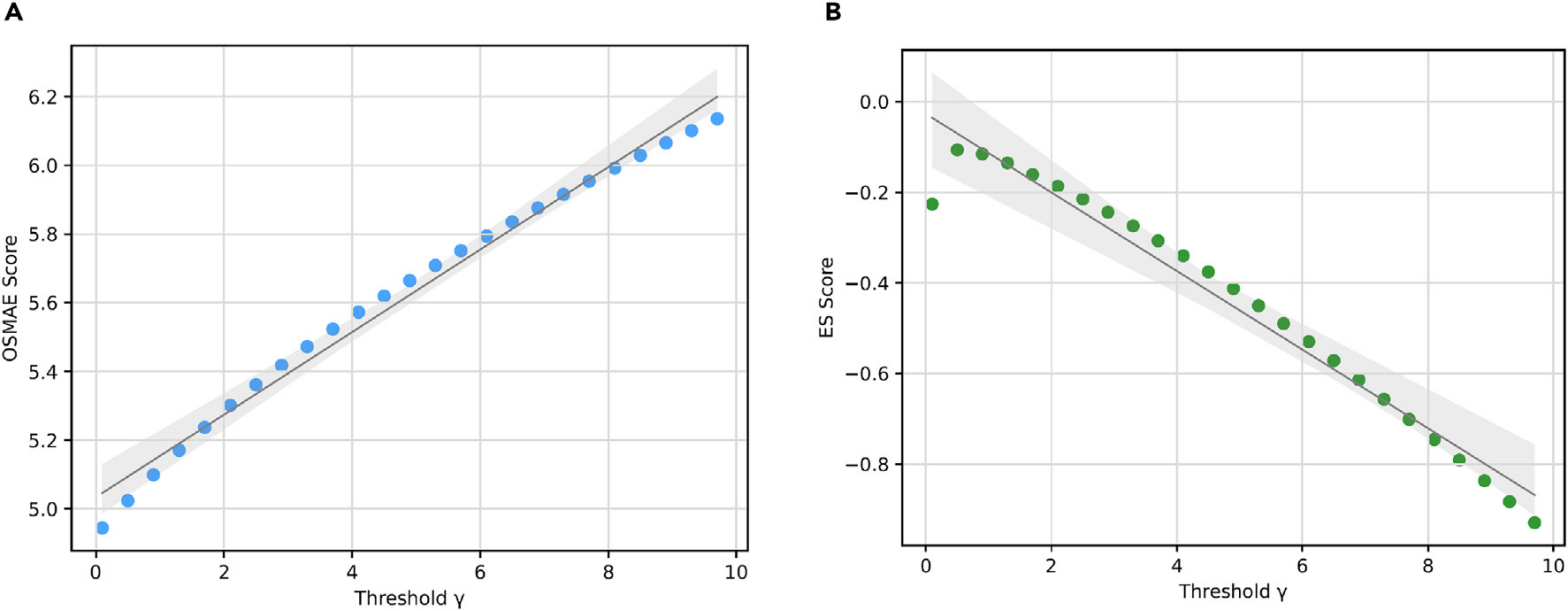
OSMAE and ES values with different γ (A) OSMAE with γ from 0 to 10. (B) ES with γ from 0 to 10. The line indicates the fitted regression line and the shadow indicates 95% confidence intervals. Scores were calculated using the same TCN multitask model on the CDSL dataset.

We investigate the influence of the threshold parameter γ on the OSMAE and the ES. The OSMAE and ES values for various γ thresholds, using the same GRU multitask model on the CDSL dataset, are plotted in Figure 7. Evidently, as γ increases, OSMAE increases and ES decreases. This occurs because a larger γ requires the model to accurately predict the target earlier, thereby increasing the task’s difficulty. In practical clinical settings, clinicians can choose an appropriate threshold based on their specific requirements. Another advantage of this γ setting is making both metrics invariant to the record length, which means patients with too many visits bring less bias to the proposed metrics compared with traditional AUROC and AUPRC. This is because both metrics are normalized by the visit time, ensuring that the length of a patient’s record does not disproportionately influence the metric values, regardless of the duration of the patient record. No matter how long the patient record is, the visits before the penalty time γ are regularized to have less effect on the metric values.

**Figure 8:**
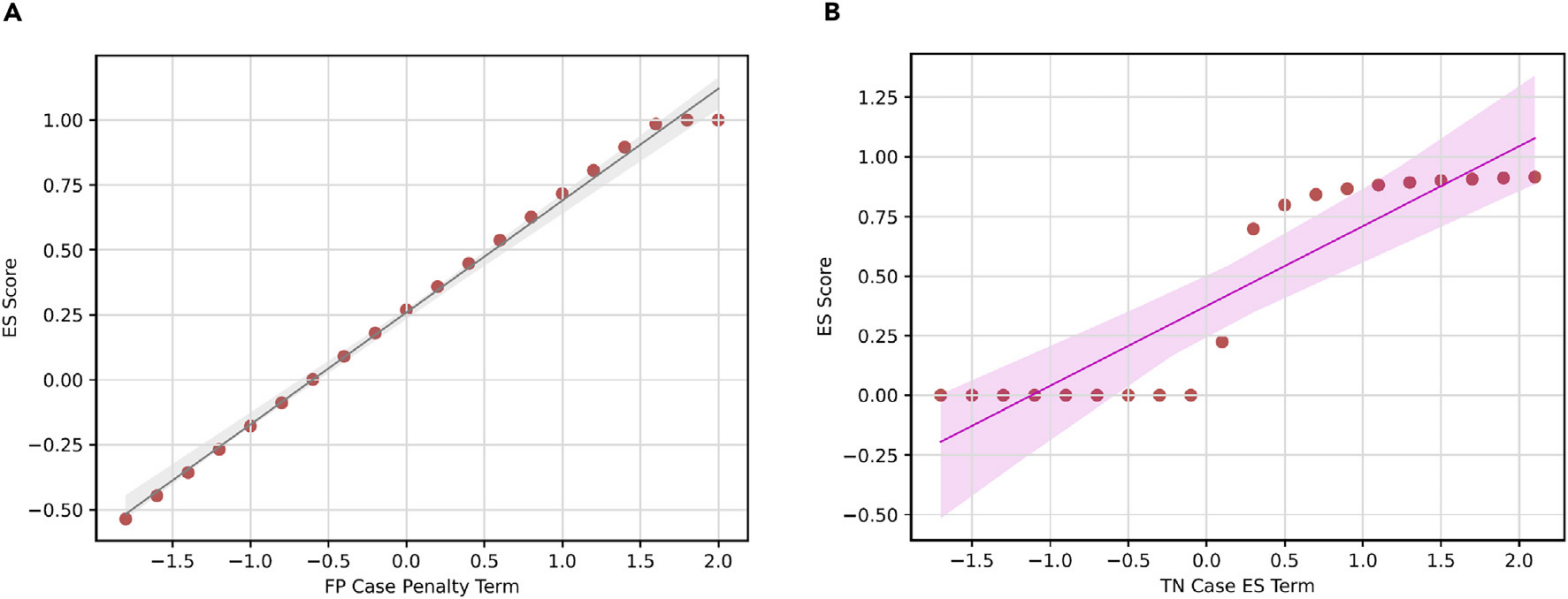
ES values with different penalty terms for a true-negative (TN) case and a false-positive (FP) case ranging from −2 to 2 (A) FP case penalty term. (B) TN case penalty term. The line indicates the fitted regression line and the shadow indicates 95% confidence intervals. Scores are calculated using the same TCN multitask model on the CDSL dataset.

We also aim to explore the effect of penalty score value in ES for false-negative and false-positive cases. We selected −0.1 as the penalty score for false-positive cases to mirror scenarios in real-world clinical settings, where the costs associated with false-negative predictions are typically higher than those for false positives, particularly in intensive care environments.^41^^,^^42^ Thus, a lower penalty score is assigned to false-positive predictions. However, clinicians may adjust this penalty score to suit their specific needs in various applications. For example, in a less critical clinical setting, clinicians may set higher penalty scores to select the model that produces fewer false alarms. Since the early-prediction score (ES) functions solely as a metric, it does not influence model performance. Figure 8 illustrates how the penalty score affects the ES value. As demonstrated in the figure, while the penalty score influences the absolute value of ES, it does not alter the underlying trend. True-negative cases receive a score of zero, aligning with the ES metric’s aim to evaluate models for early risk detection in patients. Since true-negative predictions do not trigger alarms, the model is neither rewarded nor penalized for these outcomes. It is important to note that the number of true-positive predictions is 649, compared with 10,381 true-negative predictions. Awarding points for true-negative predictions would introduce significant bias into the final score.

### Model interpretability, limitations, and future works

Interpretability is an important aspect of models, especially in clinical settings. With model interpretability, the clinicians can understand the model’s prediction process and make reliable clinical decisions. Many models benchmarked in this work claim they have different levels of interpretability. We group them into three groups: (1) visit level, (2) feature level, (3) others. Visit-level interpretability indicates that the model can identify the most important visit from longitudinal EHR sequences, and feature-level interpretability means that the model can identify the most important risk factors from single-visit data. Some models can provide other information to aid clinical decisions such as disease progression score. We summarize the characteristics of all interpretable models in Table 7.

**Table 7:** Interpretability characteristics of all interpretable models

Model	Visit level	Feature level	Others
4C	–	✓	–
RF	–	✓	–
DT	–	✓	–
GBDT	–	✓	–
CatBoost	–	✓	–
XGBoost	–	✓	–
RETAIN	✓	✓	–
StageNet	–	–	disease progression score
Dr. Agent	✓	–	–
AdaCare	–	✓	–
GRASP	–	–	patient clusters
ConCare	–	✓	feature correlations

We find that most machine-learning models are interpretable, as they are tree-based models. These models can provide only feature-level interpretability (i.e., feature-importance score) because they cannot process temporal dependencies. All naive deep-learning models are black-box models, and they are not interpretable. However, all EHR-specific deep-learning models studied in this work are designed to provide some interpretability. RETAIN can provide both visit-level and feature-level interpretabilities. StageNet, GRASP, and ConCare can provide additional information, including disease progression score, patient clustering information, and feature correlations.

We aim to establish a comprehensive benchmark for COVID-19 predictive modeling. However, our study has certain limitations. First, within our two-stage approach, the models predicting mortality and LOS might have distinct architectures. For comparison purposes, due to the inherent complexity, we assessed only scenarios where both models are identical. In addition, the multitask setting may benefit from more intricate multitask learning structures. Exploring various model combinations and learning structures is a promising avenue for future work. Second, during data preprocessing, we imputed missing values using the most recent measurement. While this method has been adopted in numerous studies,^15^^,^^27^^,^^28^ it is essential to investigate how different imputation techniques influence the outcomes in a clinical context. Last, when employing the OSMAE metric, other consequential clinical outcomes, like re-admission, in addition to mortality and recovery, should be considered. Future studies should account for these competing factors by devising more inclusive tasks and metrics. Our proposed tasks align with clinical practice requirements. Beyond task and metric design, the introduced early-prediction loss and multitask learning framework are innovative approaches yielding encouraging results for the tasks presented. We anticipate further refinements in future research endeavors.

We envision that our proposed benchmark analysis pipeline and coding framework can be extended to a wider range of clinical tasks and datasets. In this work, to bolster the comprehensiveness and validity of our evaluation, we incorporated an expansive selection of ICU datasets, namely MIMIC-III^17^^,^^43^ and MIMIC-IV.^44^^,^^45^ We undertook the in-hospital-mortality-prediction task using these two datasets, with results detailed in supplemental experimental procedures 5. While our efforts remain exploratory in nature, we aspire for our proposed tasks to be integrated across diverse clinical datasets, thereby enhancing the quality of health-care delivery. Another line for future research pertains to benchmarking the fairness of model predictions. We have undertaken preliminary experiments using the GRU model with four fairness metrics, with results detailed in supplemental experimental procedures 3. Subsequent efforts might focus on a thorough analysis aimed at minimizing bias and enhancing fairness across various predictive models.

### Conclusion

This paper presents a fair, comprehensive, and open-source benchmark for predictive modeling of COVID-19, using EHR data from ICUs. We introduce two innovative clinical prediction tasks—early-mortality prediction and outcome-specific LOS prediction—based on the clinical practice of COVID-19 prediction scenarios on two real-world datasets. Our approach ensures fairness and reproducibility through the implementation of meticulous data processing and model training pipelines. We provide benchmarking performances for a total of 17 predictive models, including 5 traditional machine-learning models, 6 basic deep-learning models, and 6 state-of-the-art EHR-specific deep-learning models. To improve accessibility, we have made our benchmarking results and trained models readily available online. This not only facilitates quick access to prediction results using our trained models, but also grants clinicians and researchers the ability to obtain swift prediction results using our trained models. Our endeavors aim to spur continuous development and advancement in the field of deep learning and machine learning, particularly in the context of pandemic predictive modeling.

## Experimental procedures

### Resource availability

#### Lead contact

Liantao Ma is the lead contact of this study and can be reached via e-mail (malt@pku.edu.cn).

#### Materials availability

This study did not generate new unique reagents.

#### Data and code availability

**Figure 9:**
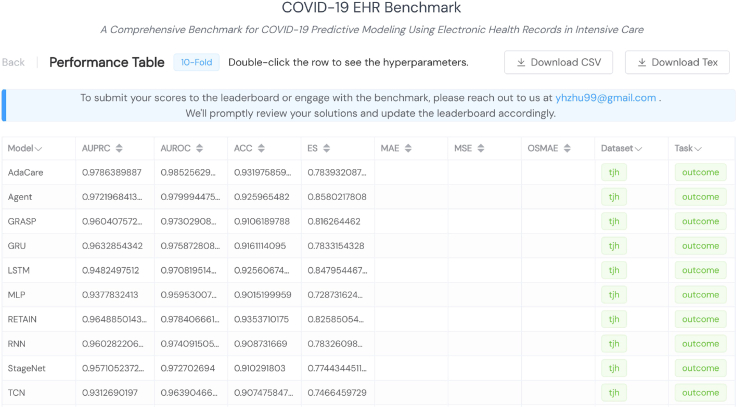
Screenshot of the online benchmark results visualization system
• The accession number for the benchmarking code reported in this paper is Zenodo: https://doi.org/10.5281/zenodo.10573567.^23^ The code structure of this benchmarking pipeline is depicted in Figure 10. The diagram is essentially self-explanatory. All runtime configurations for model parameters are stored in configs/directory. All deep-learning and machine-learning models are implemented with various modules. This clearly structured and modular design allows users to effortlessly extend the existing models, design new models and tasks, or even introduce new datasets by adding corresponding components without having an impact on the downstream benchmark prediction and evaluation process. The analytical files generated in this work are also publicly available online. These data analysis files are housed in the datasets/folder of the code repository.

• This research did not involve the collection of new patient EHR data. The TJH EHR dataset^2^ utilized in this study is publicly available on GitHub (https://github.com/HAIRLAB/Pre_Surv_COVID_19). The CDSL dataset^25^ is open to global researchers and can be accessed on request (https://hmhospitales.com/prensa/notas-de-prensa/comunicado-covid-data-save-lives). To gain access, applicants should fill out the request form. We use these datasets under their respective licenses.
• Furthermore, we have made our benchmarking system available online at https://pyehr.netlify.app, as illustrated in Figure 9. All model performances with their associated hyperparameter combinations on both tasks are accessible in the system. These results were used to generate all the performance analysis figures presented in this work. The tables are designed for easy querying, comparison, and sorting. Users can select specific rows and download the CSV files or LaTeX codes. In addition, all trained models are also released online, enabling clinicians and researchers to conveniently use these models to obtain prediction results with their own test samples. The accession number for the source code of this online system reported in this paper is Zenodo: https://doi.org/10.5281/zenodo.10573647^24^. Researchers can easily deploy an offline version.

**Figure 10:**
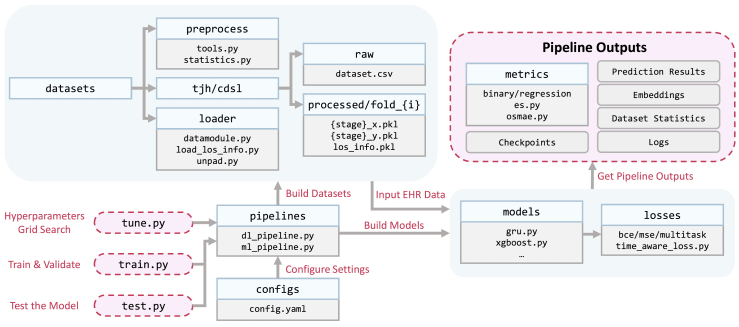
Benchmark code structure

## Supplemental information

Document S1. Figures S1–S7, Tables S1–S14, and supplemental experimental procedures

Document S2. Article plus supplemental information
