# Supplementary material for: A comprehensive benchmark for COVID-19 predictive modeling using electronic health records in intensive care
Source: Patterns (N Y). 2024 Mar 7;5(4):100951. doi: 10.1016/j.patter.2024.100951 (PMC11026964; doi:10.1016/j.patter.2024.100951)
Supplement: Document S1. Figures S1–S7, Tables S1–S14, and supplemental experimental procedures [file mmc1.pdf]

Patterns, Volume *5*

Supplemental information

A comprehensive benchmark for COVID-19 predictive modeling using electronic health records in intensive care

Junyi Gao, Yinghao Zhu, Wenqing Wang, Zixiang Wang, Guiying Dong, Wen Tang, Hao Wang, Yasha Wang, Ewen M. Harrison, and Liantao Ma

Supplemental Experimental Procedures 1
Baseline Models Descriptions

Clinical Scoring model

- **4C mortality score**[1] is a risk stratification tool that predict in-hospital mortality or in-hospital clinical deterioration (defined as any requirement of ventilatory support or critical care, or death) for hospitalised COVID-19 patients. They are designed to require only parameters that are commonly available at hospital presentation.

Machine learning models

- **Decision tree (DT)** is a non-parametric supervised learning algorithm with a hierarchical tree structure. DT has been widely applied in many clinical predictive tasks, such as mortality prediction for peritoneal dialysis patients[2].

- **Random forest (RF)** is a supervised ensemble learning method for classification, regression and other tasks. It constructs a multitude of decision trees during the training period. RF has been used to predict the patients' severity of the COVID-19 case and possible mortality outcome, recovery or death[3].

- **Gradient Boosting Decision Tree (GBDT)** is a widely-used algorithm for solving prediction problems in both classification and regression tasks. GBDT takes decision trees as the base learner and determines final prediction results based on a series of DTs' outputs. GBDT exhibits good performance in performing COVID-19 mortality prediction tasks with structured clinical data[4].

- **XGBoost**[5] is a recursive tree-based supervised machine learning classifier. XGBoost has been used to predict the mortality for COVID-19 infected patients[6].

- **CatBoost**[7] is also a gradient boost machine learning framework. It builds a symmetric tree and uses an ordered boosting strategy with a faster training and prediction speed compared to XGBoost. Catboost has been used to predict and detect the number of confirmed and death cases of COVID-19[8].

Basic deep learning models

- **Multi-layer perceptron (MLP)** is the feed-forward-based neural network. MLP has been used to predict acute kidney injury[9].

- **Recurrent neural network (RNN)**[10] is the most popular framework to learn the abstract embedding of variable-length time series. RNN has been widely used to predict the risk of the first episode of psychosis or heart failure[11,12].

- **Long-short term memory network (LSTM)**[13] is a variant of the Recurrent Neural Network (RNN), capable of learning long-term dependencies. LSTM has been used to perform the 90-day all-cause mortality in the intensive care unit (ICU), based on the concatenated static features and dynamic features[14].

- **Gated recurrent units (GRU)**[15] embeds the time series as the input to perform the target prediction. It is a widely applied variant of the Recurrent Neural Network (RNN), which improves the capability to maintain historical memories and reduces parameters in the update and reset gates. GRU has been used to predict several severe complications (mortality, renal failure with a need for renal replacement therapy, and postoperative bleeding leading to operative revision) in post-cardiosurgical care in real-time[16].

- **Temporal convolutional networks (TCN)** [17] is a generic temporal convolutional network architecture for sequence modeling. TCN has been used to forecast hospital resource utilization (i.e., the number of hospital beds and ventilators) for COVID-19 patients[18].
- **Transformer**[19] in this experiment is the encoder part of the original Transformer, which comprises the positional encoding module and the self-attention module. The transformer has been used to perform the mortality risk analysis for liver transplant recipients[20].

EHR-specific predictive models

- **RETAIN**[21] is the deep-based Reverse Time Attention model for analyzing EHR data. It utilizes a two-level neural attention module to attend important clinical visits and features. RETAIN has been used to predict heart failure by taking previous diagnoses (categorical variables) as features. In our benchmark, we modify the input layer of RETAIN with multi-layer perceptron (MLP) to make it capable of using numerical variables as features.
- **StageNet**[22] is the Stage-aware neural Network, which extracts disease stage information from EHR and integrates it into risk prediction. This model comprises a stage-aware LSTM module that extracts health stage variations unsupervisedly and a stage-adaptive convolutional module that incorporates stage-related progression patterns. StageNet has been used to perform the decompensation prediction for ICU patients in the MIMIC-III dataset and the mortality prediction of End-Stage Renal Disease (ESRD) patients in Peking University Third Hospital.
- **Dr. Agent**[23] augments RNN with 2 policy gradient agents. It learns a dynamic skip connection to focus on the relevant information over time. Dr. Agent has been used to perform the in-hospital mortality prediction, acute care phenotype classification, physiologic decompensation prediction and length of stay forecasting task on the MIMIC-III (Medical Information Mart for Intensive Care) dataset.
- **AdaCare**[24] employs a multi-scale adaptive dilated convolutional module to capture the long and short-term variations of biomarkers to depict the health status in multiple time scales. AdaCare has been used to perform decompensation prediction on the MIMIC-III dataset and the mortality prediction on the End-Stage Renal Disease (ESRD) dataset.
- **ConCare**[25] employs multi-channel embedding architecture and self-attention mechanism to model the relationship of feature sequences and build the health representation. ConCare has been used to perform the morality prediction on the MIMIC-III and ESRD datasets.
- **GRASP**[26] is a generic framework for healthcare models, which leverages the information extracted from patients with similar conditions to enhance the cohort representation learning results. GRASP has been used to perform the sepsis prediction on the cardiology dataset and mortality prediction on the ESRD dataset.

Supplemental Experimental Procedures 2
Experiment Environments and Model Hyperparameters

For DT, RF, GBDT models, we use the scikit-learn package (version 1.2.2), XGBoost (version 1.7.5), CatBoost (version 1.2) to construct and train the models. For deep learning models, we use the PyTorch (version 2.0.1) and PyTorch Lighting (version 2.0.2) to implement the models. We use the mini-batch gradient descent to train the models and the batch size is set to 64 for TJH dataset and 128 for CDSL dataset. The ζ and η are set to 0.1. For the metric calculations, E is

the maximum value at the 95% percentile of the length-of-stay of all patient in both CDSL and TJH datasets. In the hold-out set, E is 27.25 in the CDSL dataset and 21.44 in the TJH dataset. We use AdamW optimizer with tuned learning rate to train the models over 100 epochs. The model training process will be stopped if the score on the validation set do not improve over 10 consecutive iterations. We set the random seed to 0 in all computational runs.

Experiments are conducted on a server equipped with dual Intel Xeon Silver 4210R CPUs, each with 10 cores supporting 20 threads, two NVIDIA RTX 3090 GPUs and 64GB RAM. Notably, all experiments are executed solely on one of the GPUs for consistent results.

The model hyperparameters are shown in Table S1, Table S2, and S3. To perform the 4C calculations, we followed the guidelines and methodology provided at `https://isaric4c.net/risk/4c/`. Also, we have incorporated additional details about the 4C mortality score in the main text. The computation of the 4C mortality score is a straightforward process. Each patient in our study is evaluated based on a set of variables — four variables in the TJH dataset and five in the CDSL dataset. For each variable, a specific score is assigned, reflecting the patient's status in that variable. The total 4C mortality score for a patient is the sum of these individual scores. Each level of the accumulated score correlates with a distinct probability of mortality. We have presented the variables used for calculating the 4C mortality scores in both datasets in Table S4.

Table S1: *Hyperparameter settings of machine learning models.* All hyperparameters are obtained using grid-search on the validation set.

Dataset	Task	Model	Detail
TJH	outcome	RF	max depth 10, n_estimators 100, learning rate 0.1, criterion gini
	length of stay	RF	max depth 15, n_estimators 50, learning rate 1.0, criterion squared_error
	outcome	DT	max depth 10, min_samples_split 2, min_samples_leaf 1
	length of stay	DT	max depth 5, criterion squared_error, min_samples_split 2
	outcome	GBDT	max depth 5, n_estimators 100, learning rate 0.1, subsample 1.0
	length of stay	GBDT	max depth 5, n_estimators 100, learning rate 0.1, subsample 1.0
	outcome	XGBoost	max depth 5, n_estimators 100, learning rate 0.1, objective binary:logistic
	length of stay	XGBoost	max depth 5, n_estimators 100, learning rate 0.1, objective reg:squarederror
	outcome	CatBoost	max depth 5, n_estimators 100, learning rate 0.1, loss CrossEntropy
	length of stay	CatBoost	max depth 5, n_estimators 100, learning rate 0.1, loss RMSE
CDSL	outcome	RF	max depth 15, n_estimators 100, learning rate 1.0, criterion gini
	length of stay	RF	max depth 15, n_estimators 100, learning rate 1.0, criterion squared_error
	outcome	DT	max depth 5, min_samples_split 2, min_samples_leaf 1
	length of stay	DT	max depth 5, criterion squared_error, min_samples_split 2
	outcome	GBDT	max depth 5, n_estimators 100, learning rate 0.1, subsample 1.0
	length of stay	GBDT	max depth 5, n_estimators 100, learning rate 0.1, subsample 1.0
	outcome	XGBoost	max depth 5, n_estimators 50, learning rate 0.1, objective binary:logistic
	length of stay	XGBoost	max depth 10, n_estimators 50, learning rate 0.1, objective reg:squarederror
	outcome	CatBoost	max depth 5, n_estimators 50, learning rate 0.1, loss CrossEntropy
	length of stay	CatBoost	max depth 10, n_estimators 100, learning rate 0.1, loss RMSE

Supplemental Experimental Procedures 3
Evaluation of Prediction Fairness

To evaluate the models' fairness, we test the GRU model using fairness experiments. The fairness metrics include disparate impact (DI)[27], average odds difference (AOD)[28], equal opportunity difference (EOD)[28] and statistical parity difference (SPD)[29]. The results are shown in Table S5.

- **Disparate Impact (DI)[27]:** DI measures the ratio of the positive outcome rates between the unprivileged and privileged groups. The ideal value is 1.0, indicating equal positive outcome

Table S2: *Hyperparameter settings of deep learning models in the TJH dataset.* All hyperparameters are obtained using grid-search on the validation set.

Task	Model	Detail
outcome	MLP	hidden dim 32, learning rate 0.01, feed-forward dim 128
length of stay	MLP	hidden dim 128, learning rate 0.001, feed-forward dim 512
multi-task	MLP	hidden dim 32, learning rate 0.001, feed-forward dim 128
outcome	RNN	hidden dim 64, learning rate 0.001, layers 1
length of stay	RNN	hidden dim 64, learning rate 0.01, layers 1
multi-task	RNN	hidden dim 64, learning rate 0.01, layers 1
outcome	LSTM	hidden dim 128, learning rate 0.01, layers 1
length of stay	LSTM	hidden dim 128, learning rate 0.01, layers 1
multi-task	LSTM	hidden dim 128, learning rate 0.01, layers 1
outcome	GRU	hidden dim 32, learning rate 0.01, layers 1
length of stay	GRU	hidden dim 128, learning rate 0.01, layers 1
multi-task	GRU	hidden dim 32, learning rate 0.0001, layers 1
outcome	TCN	hidden dim 64, learning rate 0.0001, kernel size 2, dropout rate 0.2
length of stay	TCN	hidden dim 64, learning rate 0.01, kernel size 2, dropout rate 0.2
multi-task	TCN	hidden dim 128, learning rate 0.01, kernel size 2, dropout rate 0.2
outcome	Transformer	hidden dim 64, learning rate 0.0001, layers 1, heads 1
length of stay	Transformer	hidden dim 32, learning rate 0.001, layers 1, heads 1
multi-task	Transformer	hidden dim 32, learning rate 0.001, layers 1, heads 1
outcome	RETAIN	hidden dim 64, learning rate 0.001, dropout rate 0.1
length of stay	RETAIN	hidden dim 64, learning rate 0.001, dropout rate 0.1
multi-task	RETAIN	hidden dim 64, learning rate 0.001, dropout rate 0.1
outcome	StageNet	hidden dim 64, learning rate 0.0001, levels 3, chunk size 64
length of stay	StageNet	hidden dim 128, learning rate 0.01, levels 3, chunk size 128
multi-task	StageNet	hidden dim 32, learning rate 0.001, levels 3, chunk size 32
outcome	Dr. Agent	hidden dim 32, learning rate 0.01, actions 10, units 64
length of stay	Dr. Agent	hidden dim 64, learning rate 0.01, actions 10, units 64
multi-task	Dr. Agent	hidden dim 64, learning rate 0.01, actions 10, units 64
outcome	AdaCare	hidden dim 32, learning rate 0.0001, kernel size 2, kernel num 64, RNN type GRU
length of stay	AdaCare	hidden dim 64, learning rate 0.01, kernel size 2, kernel num 64, RNN type GRU
multi-task	AdaCare	hidden dim 64, learning rate 0.01, kernel size 2, kernel num 64, RNN type GRU
outcome	GRASP	hidden dim 128, learning rate 0.01, block GRU, clusters 12, dropout rate 0.5
length of stay	GRASP	hidden dim 64, learning rate 0.01, block GRU, clusters 12, dropout rate 0.5
multi-task	GRASP	hidden dim 32, learning rate 0.01, block GRU, clusters 12, dropout rate 0.5
outcome	ConCare	hidden dim 64, learning rate 0.001, GRU layers 1, attention None
length of stay	ConCare	hidden dim 64, learning rate 0.001, GRU layers 1, attention None
multi-task	ConCare	hidden dim 64, learning rate 0.001, GRU layers 1, attention None

Table S3: *Hyperparameter settings of deep learning models in the CDSL dataset.* All hyperparameters are obtained using grid-search on the validation set.

Task	Model	Detail
outcome	MLP	hidden dim 32, learning rate 0.001, feed-forward dim 128
length of stay	MLP	hidden dim 64, learning rate 0.001, feed-forward dim 256
multi-task	MLP	hidden dim 128, learning rate 0.001, feed-forward dim 512
outcome	RNN	hidden dim 128, learning rate 0.001, layers 1
length of stay	RNN	hidden dim 128, learning rate 0.01, layers 1
multi-task	RNN	hidden dim 32, learning rate 0.01, layers 1
outcome	LSTM	hidden dim 64, learning rate 0.001, layers 1
length of stay	LSTM	hidden dim 32, learning rate 0.0001, layers 1
multi-task	LSTM	hidden dim 32, learning rate 0.001, layers 1
outcome	GRU	hidden dim 64, learning rate 0.01, layers 1
length of stay	GRU	hidden dim 64, learning rate 0.01, layers 1
multi-task	GRU	hidden dim 32, learning rate 0.01, layers 1
outcome	TCN	hidden dim 128, learning rate 0.01, kernel size 2, dropout rate 0.2
length of stay	TCN	hidden dim 128, learning rate 0.001, kernel size 2, dropout rate 0.2
multi-task	TCN	hidden dim 128, learning rate 0.001, kernel size 2, dropout rate 0.2
outcome	Transformer	hidden dim 32, learning rate 0.01, layers 1, heads 1
length of stay	Transformer	hidden dim 128, learning rate 0.01, layers 1, heads 1
multi-task	Transformer	hidden dim 128, learning rate 0.01, layers 1, heads 1
outcome	RETAIN	hidden dim 64, learning rate 0.01, dropout rate 0.1
length of stay	RETAIN	hidden dim 128, learning rate 0.001, dropout rate 0.1
multi-task	RETAIN	hidden dim 128, learning rate 0.01, dropout rate 0.1
outcome	StageNet	hidden dim 32, learning rate 0.001, levels 3, chunk size 32
length of stay	StageNet	hidden dim 128, learning rate 0.001, levels 3, chunk size 128
multi-task	StageNet	hidden dim 32, learning rate 0.0001, levels 3, chunk size 32
outcome	Dr. Agent	hidden dim 64, learning rate 0.001, actions 10, units 64
length of stay	Dr. Agent	hidden dim 32, learning rate 0.01, actions 10, units 64
multi-task	Dr. Agent	hidden dim 32, learning rate 0.01, actions 10, units 64
outcome	AdaCare	hidden dim 32, learning rate 0.01, kernel size 2, kernel num 64, RNN type GRU
length of stay	AdaCare	hidden dim 128, learning rate 0.001, kernel size 2, kernel num 64, RNN type GRU
multi-task	AdaCare	hidden dim 64, learning rate 0.01, kernel size 2, kernel num 64, RNN type GRU
outcome	GRASP	hidden dim 32, learning rate 0.01, block GRU, clusters 12, dropout rate 0.5
length of stay	GRASP	hidden dim 32, learning rate 0.001, block GRU, clusters 12, dropout rate 0.5
multi-task	GRASP	hidden dim 128, learning rate 0.001, block GRU, clusters 12, dropout rate 0.5
outcome	ConCare	hidden dim 64, learning rate 0.001, GRU layers 1, attention None
length of stay	ConCare	hidden dim 64, learning rate 0.001, GRU layers 1, attention None
multi-task	ConCare	hidden dim 64, learning rate 0.001, GRU layers 1, attention None

rates for both groups. A value less than 1 indicates that the unprivileged group is less likely to receive the positive outcome than the privileged group. This metric is given by:

$$\text{DI} = \frac{P(Y = 1|D = \text{unprivileged})}{P(Y = 1|D = \text{privileged})}$$

Where: Y is the outcome variable (e.g., dead or alive) and D represents the group (privileged or unprivileged). For the purpose of our study, the privileged groups are identified as male and younger patients.

- **Average Odds Difference (AOD)[28]:** AOD measures the average difference of FPR and TPR for the unprivileged and privileged groups. The ideal value is 0, indicating both groups have equal FPR and TPR. The formula for AOD is:

$$\text{AOD} = \frac{1}{2}[(FPR_{D=\text{unprivileged}} - FPR_{D=\text{privileged}}) + (TPR_{D=\text{unprivileged}} - TPR_{D=\text{privileged}})]$$

Table S4: *Variables employed in the 4C mortality score calculation for both TJH and CDSL datasets.*

TJH	CDSL	4C Mortality Score Component
Sex	Sex	Sex at birth
Age	Age	Age
Urea	Urea	Urea
CRP	CRP	C reactive protein
-	SO2C	Peripheral oxygen saturation on room air

- **Equal Opportunity Difference (EOD)[28]:** EOD is similar to AOD but only considers the TPR. The ideal value is 0, indicating both groups have equal TPR. EOD is computed as:

$$\text{EOD} = TPR_{D=\text{unprivileged}} - TPR_{D=\text{privileged}}$$

- **Statistical Parity Difference (SPD)[29]:** SPD measures the difference in the probability of positive decisions between the unprivileged and privileged groups. The ideal value is 0, indicating both groups have equal probabilities of receiving a positive decision. SPD can be calculated using:

$$\text{SPD} = P(Y = 1|D = \text{unprivileged}) - P(Y = 1|D = \text{privileged})$$

The results in Table S5 show that most model predictions are more fair on the CDSL dataset than the TJH dataset, especially in terms of sex. This may be because the population size is limited in the TJH dataset and thus has induced population bias. Future work may include conducting a more comprehensive analysis to improve the bias and fairness of different predicting models.

Supplemental Experimental Procedures 4 Benchmarking Experiment under Hold-Out Data Split Settings

We employ a 10-fold cross-validation strategy to mitigate the bias associated with small sample sizes. Randomly splitting the test set can lead to unstable model performance due to its limited size. Cross-validation allows the model to be tested on all samples, thereby yielding more consistent performance conclusions. However, to evaluate the model's generalizability over time more comprehensively, we conduct a standard holdout experiment. We divide the dataset into training, validation, and test sets in a 7:1:2 ratio, based on admission times (using the latest 20% of patients as the holdout dataset, as recommended). We retrain all models five times with different random seeds and report the standard deviations. The model performances are detailed in Table S6 and Table S7.

For outcome-specific LOS prediction on the TJH dataset, AdaCare achieves the lowest MAE and MSE. On the CDSL dataset, TCN, AdaCare, and RNN exhibit the lowest MAE, MSE, and OSMAE. In the early mortality prediction task, the results align closely with those from the cross-validation setting. AdaCare and ConCare demonstrate superior performance in AUROC, AUPRC, and ES on the TJH dataset. Meanwhile, ConCare, StageNet, and GBDT achieve the best performance on the CDSL dataset. We observe significant variability in some model performances, particularly in MSE on the TJH dataset, attributable mainly to its smaller size and consequent less stable conclusions compared to the cross-validation setting. Furthermore, we note that

Table S5: *Comprehensive fairness assessment across deep learning models on TJH and CDSL datasets.* The table presents a detailed evaluation of fairness metrics, including Disparate Impact (DI), Average Odds Difference (AOD), Equal Opportunity Difference (EOD), and Statistical Parity Difference (SPD), for multiple deep learning models (e.g., MLP) across different privileged groups (Sex, Age).

Dataset		TJH				CDSL			
Metric		DI (↑)	AOD (↓)	EOD (↓)	SPD (↓)	DI (↑)	AOD (↓)	EOD (↓)	SPD (↓)
MLP	Sex	0.80±0.07	0.01±0.02	0.01±0.04	0.09±0.04	0.84±0.04	0.02±0.01	0.04±0.02	0.01±0.00
	Age	0.78±0.05	0.02±0.01	0.04±0.02	0.09±0.02	0.82±0.02	0.02±0.01	0.03±0.01	0.01±0.00
RNN	Sex	0.81±0.09	0.01±0.03	0.00±0.05	0.09±0.06	0.88±0.05	0.02±0.01	0.04±0.02	0.01±0.01
	Age	0.76±0.07	0.03±0.02	0.05±0.03	0.10±0.03	0.80±0.04	0.03±0.01	0.06±0.02	0.02±0.00
LSTM	Sex	0.82±0.09	0.00±0.03	0.02±0.03	0.09±0.06	0.89±0.06	0.02±0.01	0.03±0.03	0.01±0.01
	Age	0.79±0.06	0.02±0.02	0.03±0.02	0.09±0.02	0.81±0.03	0.03±0.01	0.06±0.02	0.02±0.00
GRU	Sex	0.81±0.10	0.01±0.04	0.01±0.06	0.10±0.07	0.89±0.06	0.01±0.01	0.02±0.03	0.01±0.01
	Age	0.76±0.07	0.03±0.02	0.05±0.03	0.10±0.02	0.82±0.05	0.03±0.01	0.06±0.02	0.02±0.00
TCN	Sex	0.81±0.09	0.00±0.03	0.00±0.05	0.09±0.06	0.88±0.07	0.02±0.01	0.05±0.03	0.01±0.01
	Age	0.76±0.06	0.03±0.01	0.06±0.03	0.10±0.02	0.79±0.03	0.03±0.01	0.06±0.02	0.02±0.00
Transformer	Sex	0.83±0.09	0.00±0.03	0.02±0.03	0.09±0.06	0.86±0.05	0.01±0.01	0.01±0.03	0.00±0.01
	Age	0.80±0.06	0.01±0.01	0.02±0.02	0.09±0.02	0.79±0.05	0.02±0.01	0.04±0.02	0.01±0.01
RETAIN	Sex	0.79±0.09	0.01±0.02	0.00±0.04	0.10±0.06	0.85±0.08	0.02±0.01	0.05±0.03	0.01±0.01
	Age	0.76±0.06	0.02±0.02	0.05±0.03	0.10±0.02	0.77±0.02	0.03±0.01	0.06±0.02	0.01±0.00
StageNet	Sex	0.80±0.10	0.01±0.03	0.00±0.06	0.10±0.07	0.89±0.06	0.02±0.01	0.03±0.02	0.01±0.01
	Age	0.76±0.07	0.03±0.02	0.06±0.03	0.10±0.03	0.81±0.04	0.03±0.01	0.06±0.02	0.02±0.01
Dr. Agent	Sex	0.80±0.09	0.01±0.02	0.01±0.03	0.10±0.06	0.86±0.06	0.02±0.02	0.03±0.04	0.01±0.01
	Age	0.79±0.05	0.01±0.01	0.03±0.01	0.09±0.02	0.82±0.06	0.03±0.01	0.06±0.02	0.02±0.01
AdaCare	Sex	0.79±0.10	0.01±0.02	0.01±0.04	0.10±0.06	0.89±0.06	0.02±0.01	0.04±0.02	0.01±0.01
	Age	0.77±0.06	0.02±0.01	0.03±0.03	0.09±0.03	0.83±0.03	0.03±0.01	0.05±0.02	0.01±0.00
GRASP	Sex	0.81±0.09	0.01±0.03	0.01±0.04	0.10±0.06	0.90±0.08	0.01±0.01	0.02±0.02	0.01±0.01
	Age	0.79±0.06	0.02±0.01	0.04±0.02	0.09±0.02	0.79±0.04	0.03±0.01	0.06±0.02	0.02±0.01
ConCare	Sex	0.79±0.08	0.01±0.02	0.00±0.04	0.10±0.05	0.88±0.06	0.03±0.01	0.05±0.03	0.01±0.01
	Age	0.77±0.06	0.02±0.01	0.04±0.02	0.10±0.02	0.78±0.05	0.03±0.01	0.06±0.02	0.02±0.00

overall model performance on the hold-out dataset is inferior to that in the cross-validation setting, suggesting a shift in dataset distribution over time and a corresponding decline in the models' predictive capabilities. Overall, AdaCare, Dr. Agent and StageNet are top-performing models for both tasks on both datasets. For a light-weight choice, GBDT outperformed other machine learning models and could be considered as the choice for machine learning models.

Supplemental Experimental Procedures 5 Benchmarking Experiment on MIMIC Datasets

To enhance the comprehensiveness and validity of the evaluation, we include a broader array of ICU datasets (MIMIC-III[30] and MIMIC-IV[31]). We conduct the in-hospital mortality prediction task on the two datasets. The label definition and data preprocessing are following previous benchmark works[32], but we provide a more comprehensive baseline comparison. The results are shown in Table S8.

We find that Dr. Agent and StageNet achieve better performance on the MIMIC-III dataset, while Dr. Agent and GRU achieve better performance on the MIMIC-IV dataset. This conclusion

Table S6: *Benchmarking performance of outcome-specific length-of-stay prediction on TJH and CDSL hold-out test sets.*. The reported score is in the form of $mean \pm std$. Subscript m signifies a multi-task learning strategy, while subscript t indicates a two-stage learning strategy. **Bold** denotes the best performance. Underline indicates that the multi-task setting outperforms the two-stage learning strategy. The asterisk * denotes that the performance improvement against the two-stage model is statistically significant (p-value < 0.05).

Dataset	TJH			CDSL		
Metric	MAE(↓)	MSE(↓)	OSMAE(↓)	MAE(↓)	MSE(↓)	OSMAE(↓)
RF$_t$	5.81 ± 0.18	45.89 ± 2.03	8.18 ± 0.19	4.21 ± 0.00	40.47 ± 0.05	4.24 ± 0.01
DT$_t$	5.00 ± 0.06	51.22 ± 1.83	9.34 ± 0.54	4.34 ± 0.00	41.82 ± 0.00	4.43 ± 0.00
GBDT$_t$	5.98 ± 0.06	50.39 ± 1.40	**7.21 ± 0.16**	4.24 ± 0.00	40.58 ± 0.02	4.27 ± 0.01
CatBoost$_t$	5.51 ± 0.14	41.25 ± 2.12	7.71 ± 0.33	4.22 ± 0.00	40.49 ± 0.08	4.24 ± 0.01
XGBoost$_t$	5.84 ± 0.00	46.86 ± 0.00	7.87 ± 0.00	4.22 ± 0.00	40.57 ± 0.00	4.23 ± 0.00
MLP$_t$	5.98 ± 0.27	51.09 ± 5.21	9.77 ± 1.18	4.22 ± 0.02	40.52 ± 0.04	4.20 ± 0.02
MLP$_m$	6.35 ± 0.24	71.81 ± 17.97	9.19 ± 0.27*	4.22 ± 0.04	40.14 ± 0.27*	4.19 ± 0.07
RNN$_t$	5.56 ± 0.11	41.27 ± 1.42	8.73 ± 0.45	4.08 ± 0.03	41.10 ± 1.42	**4.00 ± 0.05**
RNN$_m$	7.81 ± 0.33	87.99 ± 9.73	12.14 ± 0.78	4.11 ± 0.06	**39.25 ± 0.61***	4.15 ± 0.15
LSTM$_t$	5.28 ± 0.57	40.00 ± 11.06	8.65 ± 0.60	4.06 ± 0.02	39.71 ± 0.14	4.06 ± 0.04
LSTM$_m$	6.71 ± 0.48	70.72 ± 9.57	10.94 ± 1.12	4.18 ± 0.07	40.33 ± 0.60	4.25 ± 0.10
GRU$_t$	5.87 ± 0.31	47.79 ± 5.05	8.80 ± 0.70	4.08 ± 0.07	40.57 ± 2.20	4.03 ± 0.09
GRU$_m$	5.01 ± 0.29*	32.03 ± 3.06*	9.44 ± 1.47	4.17 ± 0.03	40.28 ± 1.63*	4.29 ± 0.09
TCN$_t$	6.29 ± 0.84	77.96 ± 34.06	9.53 ± 0.93	4.08 ± 0.06	40.38 ± 0.27	4.12 ± 0.24
TCN$_m$	7.50 ± 1.16	162.34 ± 108.99	11.93 ± 1.23	**4.04 ± 0.03***	39.39 ± 0.41*	4.03 ± 0.07*
Transformer$_t$	6.38 ± 0.20	62.65 ± 12.11	11.11 ± 0.91	4.14 ± 0.03	41.23 ± 0.55	4.60 ± 0.25
Transformer$_m$	6.46 ± 0.19	74.44 ± 26.43	11.27 ± 1.35	4.16 ± 0.06	43.27 ± 1.13	4.54 ± 0.27
RETAIN$_t$	5.36 ± 0.33	39.42 ± 5.26	12.60 ± 2.19	4.17 ± 0.08	40.87 ± 0.35	4.24 ± 0.09
RETAIN$_m$	6.30 ± 0.57	115.36 ± 72.40	12.63 ± 1.56	4.11 ± 0.02*	39.41 ± 0.49*	4.35 ± 0.15
StageNet$_t$	5.49 ± 0.85	47.51 ± 22.86	9.45 ± 0.46	4.08 ± 0.03	40.75 ± 1.04	4.14 ± 0.08
StageNet$_m$	5.10 ± 0.43*	33.51 ± 6.01*	15.09 ± 7.87	4.09 ± 0.02	40.40 ± 0.43	4.16 ± 0.02
Dr. Agent$_t$	6.36 ± 0.29	57.96 ± 3.57	9.69 ± 0.84	4.09 ± 0.03	41.17 ± 1.03	4.28 ± 0.20
Dr. Agent$_m$	7.06 ± 0.43	72.02 ± 2.90	10.15 ± 1.17	4.13 ± 0.09	39.83 ± 0.95*	4.22 ± 0.19
AdaCare$_t$	**3.58 ± 0.66**	**19.65 ± 5.81**	9.26 ± 1.58	**4.04 ± 0.01**	40.37 ± 0.86	4.10 ± 0.08
AdaCare$_m$	5.13 ± 0.76	34.44 ± 8.48	16.89 ± 8.79	4.12 ± 0.19	43.40 ± 4.03	4.11 ± 0.12
GRASP$_t$	5.40 ± 0.53	40.75 ± 10.74	8.94 ± 0.74	4.20 ± 0.07	41.00 ± 0.20	4.29 ± 0.10
GRASP$_m$	5.80 ± 0.90	47.69 ± 18.62	11.91 ± 4.34	4.18 ± 0.06	41.03 ± 0.93	4.31 ± 0.09
ConCare$_t$	4.79 ± 0.44	29.92 ± 5.63	8.15 ± 0.22	4.16 ± 0.02	41.12 ± 0.45	4.08 ± 0.03
ConCare$_m$	7.62 ± 0.20	78.77 ± 6.50	10.24 ± 0.43	4.13 ± 0.16	39.36 ± 0.62*	4.12 ± 0.23

Table S7: *Benchmarking performance on the task of early mortality prediction on TJH and CDSL hold-out test sets.* The reported score is of the form $mean \pm std$. 'TA' denotes the model trained with the time-aware loss. **Bold** denotes the best performance. Underline indicates that the model with time-aware loss outperforms the original model. The asterisk * denotes that the performance improvement against the model without TA version is statistically significant (p-value < 0.05). All three metrics are multiplied by 100 for readability purposes.

Dataset	TJH			CDSL		
Metric	AUPRC(↑)	AUROC(↑)	ES(↑)	AUPRC(↑)	AUROC(↑)	ES(↑)
RF	99.48 ± 0.04	98.13 ± 0.19	52.16 ± 2.32	52.16 ± 2.32	83.28 ± 0.21	-8.08 ± 0.30
DT	94.31 ± 0.80	85.68 ± 1.80	44.84 ± 3.33	27.71 ± 0.00	76.58 ± 0.00	-4.47 ± 0.00
GBDT	99.34 ± 0.03	97.57 ± 0.11	50.80 ± 0.62	41.91 ± 0.28	**84.71** ± **0.04**	3.55 ± 0.35
CatBoost	99.38 ± 0.11	97.74 ± 0.39	67.62 ± 6.16	40.00 ± 0.36	84.17 ± 0.23	-6.91 ± 0.78
XGBoost	99.46 ± 0.00	98.17 ± 0.00	39.92 ± 0.00	40.32 ± 0.00	84.44 ± 0.00	-1.22 ± 0.00
MLP	99.03 ± 0.82	97.63 ± 0.98	69.16 ± 8.37	39.63 ± 0.79	83.41 ± 0.36	-3.68 ± 1.24
MLP-TA	98.75 ± 0.74	96.94 ± 1.01	69.44 ± 6.61	40.46 ± 0.89*	83.49 ± 0.32	-2.26 ± 2.14*
RNN	99.69 ± 0.14	98.94 ± 0.46	75.56 ± 4.28	41.91 ± 1.20	83.36 ± 0.90	5.44 ± 3.40
RNN-TA	99.71 ± 0.13*	98.99 ± 0.44*	75.78 ± 4.04	42.65 ± 1.17*	83.35 ± 0.63	7.00 ± 5.62*
LSTM	99.14 ± 0.14	97.50 ± 0.24	73.70 ± 1.79	39.76 ± 1.40	81.73 ± 0.77	10.56 ± 1.76
LSTM-TA	99.16 ± 0.14	97.56 ± 0.23	74.15 ± 2.61*	40.77 ± 1.32*	82.39 ± 0.53*	13.03 ± 2.66*
GRU	99.43 ± 0.25	98.07 ± 0.78	75.74 ± 3.88	41.43 ± 1.93	83.28 ± 0.90	5.38 ± 5.13
GRU-TA	99.48 ± 0.20	98.21 ± 0.64*	76.42 ± 2.06*	42.18 ± 1.35*	83.64 ± 0.82*	9.01 ± 5.59*
TCN	98.71 ± 0.23	96.84 ± 0.25	74.38 ± 1.29	39.64 ± 1.48	83.50 ± 0.44	4.17 ± 8.71
TCN-TA	98.72 ± 0.20	96.84 ± 0.22	73.70 ± 1.79	40.61 ± 1.68*	83.46 ± 0.44	3.45 ± 3.26
Transformer	99.06 ± 0.86	97.84 ± 1.23	64.22 ± 6.73	31.18 ± 4.86	80.04 ± 1.15	-1.93 ± 12.86
Transformer-TA	99.53 ± 0.22	98.52 ± 0.56	64.22 ± 6.81	32.31 ± 2.44*	80.89 ± 1.22*	9.48 ± 6.30*
RETAIN	97.23 ± 1.21	92.70 ± 2.90	50.39 ± 15.40	37.65 ± 2.17	81.84 ± 1.33	1.92 ± 9.56
RETAIN-TA	97.50 ± 1.11	93.14 ± 2.82	52.66 ± 16.36	38.83 ± 2.30*	82.42 ± 1.65*	5.31 ± 9.79*
StageNet	99.35 ± 0.26	97.94 ± 0.64	70.07 ± 2.59	42.08 ± 0.53	83.09 ± 0.36	12.88 ± 3.08
StageNet-TA	99.38 ± 0.25*	98.06 ± 0.63*	70.98 ± 3.98*	42.51 ± 0.52*	83.17 ± 0.32	**14.06** ± **2.80***
Dr. Agent	99.35 ± 0.24	97.85 ± 0.57	72.57 ± 3.84	40.83 ± 1.18	82.98 ± 0.78	11.18 ± 6.92
Dr. Agent-TA	99.37 ± 0.24	97.92 ± 0.57	73.47 ± 2.93*	41.87 ± 1.45*	83.07 ± 1.12*	12.46 ± 6.58*
AdaCare	99.85 ± 0.13	99.49 ± 0.41	62.32 ± 10.08	40.78 ± 0.94	83.46 ± 0.12	8.27 ± 1.81
AdaCare-TA	**99.87** ± **0.09***	**99.53** ± **0.32***	69.35 ± 4.41*	41.90 ± 1.15*	83.72 ± 0.28*	9.70 ± 1.59*
GRASP	99.42 ± 0.16	98.19 ± 0.39	72.79 ± 4.63	33.13 ± 1.10	75.04 ± 0.74	-2.32 ± 6.00
GRASP-TA	99.49 ± 0.22	98.40 ± 0.57*	72.79 ± 4.90	34.57 ± 1.12*	75.43 ± 1.20	1.92 ± 2.39
ConCare	99.50 ± 0.20	98.24 ± 0.55	77.33 ± 2.82	43.12 ± 0.09	84.39 ± 0.08	-1.23 ± 5.16
ConCare-TA	99.49 ± 0.24	98.22 ± 0.66	**78.91** ± **1.72***	**43.47** ± **0.14***	84.28 ± 0.17	2.55 ± 3.38*

is similar to the CDSL and TJH dataset, which proves the generalizability of these models. Since the LOS information in the two MIMIC datasets has high variance, we do not report the OSMAE on these datasets. In future works, these metrics can be applied to more specific cohorts that have lower LOS variance.

Similar to the TJH and CDSL datasets, we also explore the early prediction setting on the MIMIC-III and MIMIC-IV datasets. As shown in Figure S1, the time-aware loss can effectively improve various models' prediction performance on more general datasets. The MIMIC dataset statistics used in our experiments are shown in Table S9 and S10.

Table S8: *Benchmarking performance on the task of early mortality prediction on MIMIC-III and MIMIC-IV hold-out test sets.* The reported score is of the form $mean \pm std$. 'TA' denotes the model trained with the time-aware loss. **Bold** denotes the best performance. Underline indicates that the model with time-aware loss outperforms the original model. The asterisk * denotes that the performance improvement against the model without TA version is statistically significant (p-value < 0.05). All three metrics are multiplied by 100 for readability purposes.

Dataset	MIMIC-III			MIMIC-IV		
Metric	AUPRC(↑)	AUROC(↑)	ES(↑)	AUPRC(↑)	AUROC(↑)	ES(↑)
RF	43.56 ± 0.24	81.94 ± 0.08	-1.58 ± 0.31	39.46 ± 0.21	80.58 ± 0.06	0.50 ± 0.23
DT	31.35 ± 0.16	70.65 ± 0.17	7.45 ± 0.20	29.56 ± 0.22	74.04 ± 0.22	9.49 ± 0.16
GBDT	45.73 ± 0.02	83.67 ± 0.00	11.80 ± 0.03	42.06 ± 0.04	81.80 ± 0.01	13.57 ± 0.05
CatBoost	45.44 ± 0.34	83.50 ± 0.08	6.72 ± 0.14	41.44 ± 0.11	81.66 ± 0.04	8.67 ± 0.27
XGBoost	45.73 ± 0.20	83.65 ± 0.04	10.68 ± 0.43	41.54 ± 0.00	81.76 ± 0.00	12.29 ± 0.00
MLP	45.01 ± 0.28	83.04 ± 0.07	12.73 ± 1.51	41.39 ± 0.54	81.35 ± 0.17	10.66 ± 1.29
MLP-TA	44.92 ± 0.57	82.86 ± 0.18	13.68 ± 1.70*	40.97 ± 0.35	81.18 ± 0.14	13.80 ± 1.22*
RNN	45.87 ± 0.61	83.34 ± 0.25	13.45 ± 0.96	44.08 ± 0.21	82.85 ± 0.10	15.93 ± 2.35
RNN-TA	45.38 ± 0.52	82.99 ± 0.20	13.69 ± 1.12	43.59 ± 0.34	82.59 ± 0.16	**17.88 ± 1.81***
LSTM	47.46 ± 0.34	83.66 ± 0.06	15.35 ± 1.44	44.52 ± 0.49	83.14 ± 0.21	15.27 ± 1.73
LSTM-TA	47.14 ± 0.35	83.29 ± 0.15	15.70 ± 1.32	44.34 ± 0.44	82.86 ± 0.15	17.37 ± 1.65*
GRU	47.19 ± 0.45	83.80 ± 0.18	14.05 ± 0.70	**45.58 ± 0.18**	83.50 ± 0.09	15.43 ± 1.60
GRU-TA	46.87 ± 0.45	83.39 ± 0.19	15.35 ± 2.62*	45.33 ± 0.22	83.18 ± 0.14	17.44 ± 2.03*
TCN	47.71 ± 0.46	84.17 ± 0.13	**18.72 ± 1.85**	45.00 ± 0.51	**83.53 ± 0.23**	16.42 ± 2.34
TCN-TA	47.73 ± 0.24	84.04 ± 0.13	18.10 ± 2.31	45.03 ± 0.59	83.47 ± 0.20	17.74 ± 1.75*
Transformer	42.51 ± 0.49	82.43 ± 0.16	8.55 ± 1.88	42.32 ± 0.24	82.54 ± 0.16	10.12 ± 3.95
Transformer-TA	42.38 ± 0.54	82.24 ± 0.18	9.68 ± 2.31*	42.24 ± 0.36	82.54 ± 0.14	13.77 ± 4.17*
RETAIN	46.72 ± 0.44	83.61 ± 0.23	6.72 ± 1.94	44.89 ± 0.54	83.34 ± 0.26	6.65 ± 0.59
RETAIN-TA	46.49 ± 0.35	83.21 ± 0.15	8.27 ± 1.86*	44.48 ± 0.25	82.68 ± 0.22	7.33 ± 1.19*
StageNet	47.33 ± 0.38	83.60 ± 0.16	16.66 ± 2.23	44.79 ± 0.26	83.21 ± 0.35	15.62 ± 0.77
StageNet-TA	46.81 ± 0.41	83.22 ± 0.19	16.49 ± 1.26	44.22 ± 0.44	82.70 ± 0.33	17.68 ± 3.12*
Dr. Agent	47.94 ± 0.33	**84.30 ± 0.10**	15.71 ± 2.32	44.53 ± 0.69	83.15 ± 0.24	13.54 ± 1.53
Dr. Agent-TA	**48.10 ± 0.23***	84.17 ± 0.13	16.04 ± 2.59*	44.00 ± 0.29	82.67 ± 0.15	13.84 ± 1.64
AdaCare	47.32 ± 0.37	83.62 ± 0.33	15.90 ± 1.16	44.41 ± 0.13	82.74 ± 0.13	15.33 ± 2.10
AdaCare-TA	47.29 ± 0.24	83.49 ± 0.23	15.45 ± 1.85	44.19 ± 0.16	82.42 ± 0.11	17.78 ± 2.22*
GRASP	46.96 ± 0.19	83.55 ± 0.11	13.95 ± 1.66	44.22 ± 0.31	82.87 ± 0.12	14.54 ± 0.81
GRASP-TA	46.64 ± 0.33	83.13 ± 0.18	14.13 ± 1.56	44.03 ± 0.41	82.49 ± 0.15	16.03 ± 1.33*
ConCare	46.40 ± 0.30	83.03 ± 0.10	15.30 ± 1.25	44.10 ± 0.47	82.70 ± 0.22	13.92 ± 3.04
ConCare-TA	45.91 ± 0.49	82.74 ± 0.26	15.29 ± 0.91	43.92 ± 0.46	82.48 ± 0.18	15.62 ± 1.55*

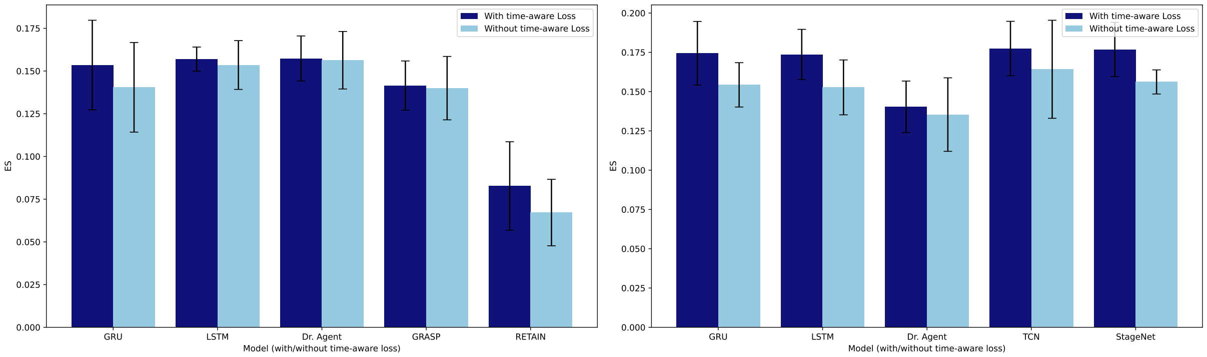

(a) MIMIC-III (b) MIMIC-IV

Figure S1: *Early prediction performance of 5 models with the highest ES on the MIMIC-III and MIMIC-IV datasets.* All models are trained using the first half of patient records. Error bars are standard deviations.

Table S9: *Statistics of the MIMIC-III dataset.* The reported statistics are of the form $Median[Q1, Q3]$.

Mortality Outcome	Total	Alive	Dead
# Patients	41517	37108 (89.38 %)	4409 (10.62 %)
# Records	3509005	2952672 (84.15 %)	556333 (15.85 %)
# Avg. records	46.0 [26.0, 88.0]	45.0 [26.0, 80.0]	66.0 [26.0, 154.0]
Age	65.5 [52.4, 77.9]	64.5 [51.6, 77.0]	74.6 [60.7, 83.3]
Age > Avg. (75.1)	12852(30.96 %)	10703(28.84 %)	2149(48.74 %)
Age $\leq$ Avg. (75.1)	28665(69.04 %)	26405(71.16 %)	2260(51.26 %)
Gender	55.9% Male	56.3% Male	52.8% Male
Male	23214(55.91 %)	20885(56.28 %)	2329(52.82 %)
Female	18303(44.09 %)	16223(43.72 %)	2080(47.18 %)
# Features		61	
Length of stay	46.9 [26.5, 88.5]	45.9 [26.3, 80.5]	68.6 [29.3, 158.8]

Table S10: *Statistics of the MIMIC-IV dataset.* The reported statistics are of the form $Median[Q1, Q3]$.

Mortality Outcome	Total	Alive	Dead
# Patients	56888	51451 (90.44 %)	5437 (9.56 %)
# Records	4055519	3466575 (85.48 %)	588944 (14.52 %)
# Avg. records	42.0 [24.0, 75.0]	41.0 [24.0, 72.0]	59.0 [24.0, 137.0]
Age	65.0 [53.0, 76.0]	64.0 [52.0, 75.0]	72.0 [60.0, 82.0]
Age > Avg. (63.1)	29950(52.65 %)	26232(50.98 %)	3718(68.38 %)
Age $\leq$ Avg. (63.1)	26938(47.35 %)	25219(49.02 %)	1719(31.62 %)
Gender	55.7% Male	55.8% Male	54.1% Male
Male	31669(55.67 %)	28726(55.83 %)	2943(54.13 %)
Female	25219(44.33 %)	22725(44.17 %)	2494(45.87 %)
# Features		61	
Length of stay	42.1 [24.0, 75.2]	41.0 [24.0, 71.9]	61.2[25.2, 138.6]

Supplemental Experimental Procedures 6
Model Embedding Visualization for Two-Stage and Multi-task Settings

To more effectively illustrate the learned embeddings from both model types, we employ t-SNE for visualizing the hidden states of patient embeddings. As depicted in Figure S2, the left figure presents the embeddings of the TCN model under a two-stage setting, while the right figure displays the embeddings from a multi-task setting. The embeddings derived under the multi-task setting appear more clustered, suggesting that the model is capable of learning more consistent embeddings in the latent space. This clustering is likely due to the model being tasked with optimizing for two objectives simultaneously.

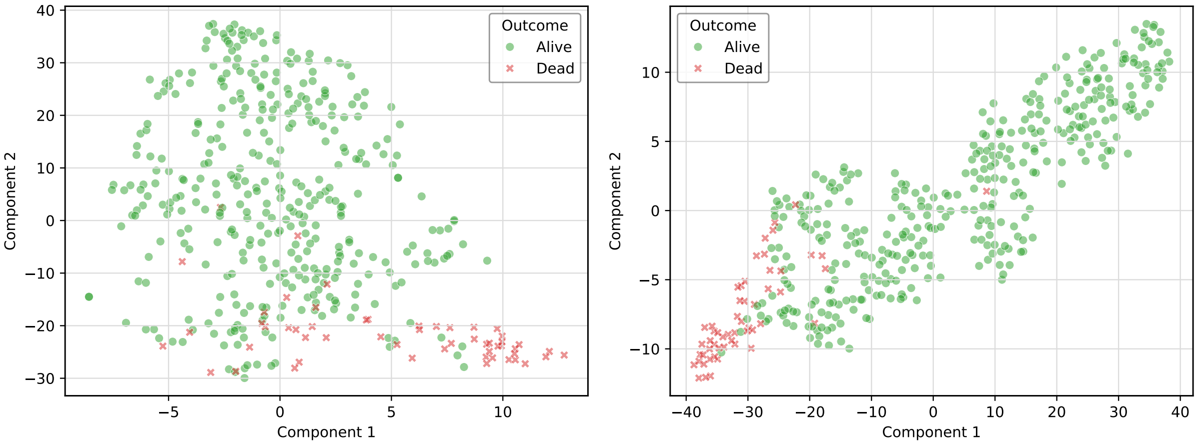

(a) TCN model's embedding under two-stage setting

(b) TCN model's embedding under multi-task setting

Figure S2: *Visualization of TCN model's embedding for LOS prediction under two settings using t-SNE.* The embeddings are extracted at each patient's last visit on the CDSL dataset.

Supplemental Experimental Procedures 7
# P-Values of T-Tests

To assess the statistical significance of our model's performance, we performed t-tests on all results. The bootstrap T-test[33] was employed to calculate p-values. We set the sample number for the bootstrap at 1000 and the number of sampling processes at 50, including all results from the 10-fold prediction process. The comparisons were made between different variations of the model — for instance, comparing the model with and without Time-Aware (TA) loss in the early mortality prediction task, and between two-stage and multi-task settings in the LOS prediction task. We report the p-value of AUPRC in the early mortality prediction task and MAE in the LOS prediction task in Table S11 and S12.

Table S11: *P-values of two-stage vs. multi-task performance in LOS predictions using t-test.* P-values are rounded to three decimal places; a value of 0.000 indicates a rounded p-value < 0.0005.

Dataset	TJH			CDSL		
Model	MAE	MSE	OSMAE	MAE	MSE	OSMAE
MLP	0.000	0.000	0.738	0.000	0.000	0.000
RNN	0.000	0.000	0.000	0.000	0.000	0.000
LSTM	0.000	0.000	0.000	0.000	0.000	0.000
GRU	0.000	0.011	0.000	0.000	0.000	0.000
TCN	0.000	0.000	0.000	0.000	0.000	0.000
Transformer	0.000	0.349	0.000	0.000	0.000	0.000
RETAIN	0.000	0.000	0.000	0.000	0.000	0.000
StageNet	0.000	0.000	0.000	0.000	0.000	0.000
Dr. Agent	0.000	0.000	0.000	0.000	0.000	0.000
AdaCare	0.000	0.000	0.000	0.000	0.000	0.000
GRASP	0.000	0.000	0.000	0.000	0.000	0.000
ConCare	0.000	0.000	0.000	0.000	0.000	0.000

Table S12: *P-values of naive models vs. models with the TA loss in early mortality predictions using t-test.* P-values are rounded to three decimal places; a value of 0.000 indicates a rounded p-value < 0.0005.

Dataset	TJH			CDSL		
Model	AUPRC	AUROC	ES	AUPRC	AUROC	ES
MLP	0.000	0.000	0.195	0.896	0.570	0.430
RNN	0.136	0.274	0.001	0.000	0.000	0.000
LSTM	0.000	0.000	0.042	1.000	0.378	1.000
GRU	0.584	0.176	0.000	0.000	0.000	0.000
TCN	0.132	0.002	0.092	0.972	0.540	0.005
Transformer	0.817	0.881	0.000	0.966	0.000	0.000
RETAIN	0.000	0.000	0.000	1.000	1.000	1.000
StageNet	0.000	0.000	0.003	0.010	0.010	1.000
Dr. Agent	0.030	0.000	0.236	0.000	0.000	0.000
AdaCare	0.586	0.118	0.000	0.700	0.964	0.007
GRASP	0.151	0.176	0.135	0.000	0.000	0.000
ConCare	0.380	0.953	0.001	0.815	0.755	1.000

Supplemental Experimental Procedures 8
Error Analysis

To more effectively analyze the sources of MAE, we plotted the MAE distributions for groups of patients who were alive and deceased. Additionally, we segmented each patient's record into two halves: the initial half and the latter half. As depicted in Figure S3, we observe that the MAE distributions for the first halves of the records are similar across both patient groups, with many records of MAE greater than 8. This is primarily because, in the initial stages of their hospital stay, patients' health statuses are often unstable, making it challenging for the model to accurately predict the remaining LOS. Conversely, during the latter half of the patients' stay, their health status tends to be more defined, resulting in a left-skewed MAE distribution for both groups. Notably, the MAE for deceased patients is predominantly less than 3, reflecting the more apparent nature of their health status and easier classification. In contrast, predicting LOS for alive patients is more complex, as their discharge from the ICU can be influenced by various external factors. Hence, their MAE values are larger (mostly clustered between 3 and 4).

These findings suggest that while the average MAE for the total cohort may be as large as the true LOS of patients, it can still effectively identify patients in critical condition, as indicated by the generally low MAE values for such cases. On the CDSL dataset, the similarity in MAE values is attributed to the fact that 87% of the patients are alive, with their MAE predominantly ranging between 2 and 4, leading to closely aligned average values.

Supplemental Experimental Procedures 9
Feature Statistics and Distributions

We provide statistics of all features which are used in the modeling process in Table S13 and S14. The data preprocessing details are shown in Figure S4. The length of stay distributions are shown in Figure S5. We plot the distributions of 16 features with the lowest missing rates in two datasets in Figure S6 and Figure S7.

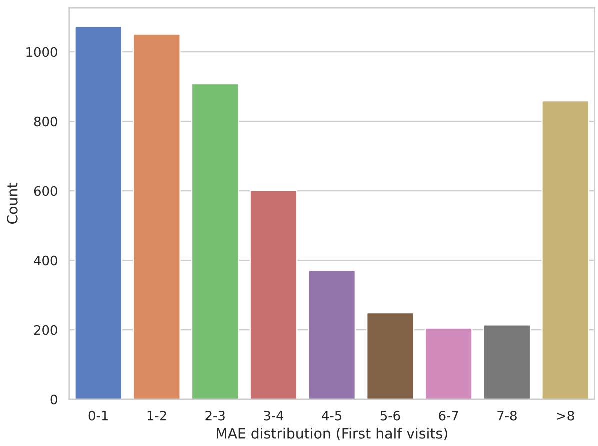

(a) The first half of records (alive patients)

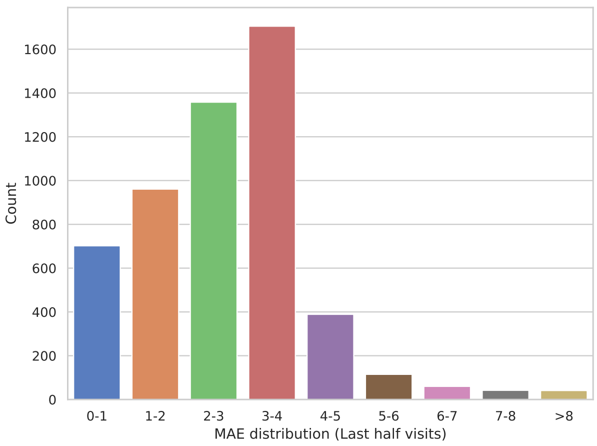

(b) The last half of records (alive patients)

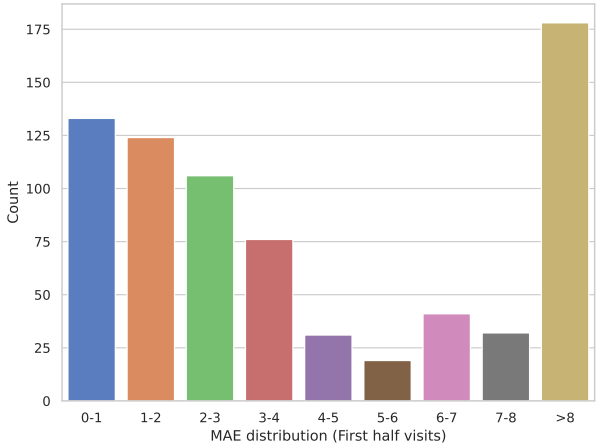

(c) The first half of records (deceased patients)

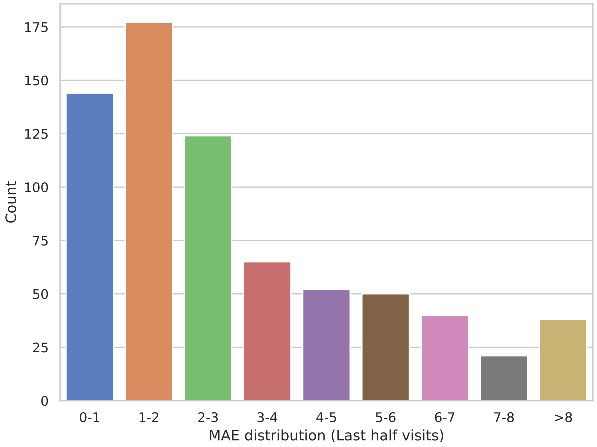

(d) The last half of records (deceased patients)

Figure S3: *MAE score distribution on the CDSL test set.* The figure compares the discrepancies in MAE performance distributions between the first and last half of patient records. The analysis utilizes the StageNet model with the multi-task setting.

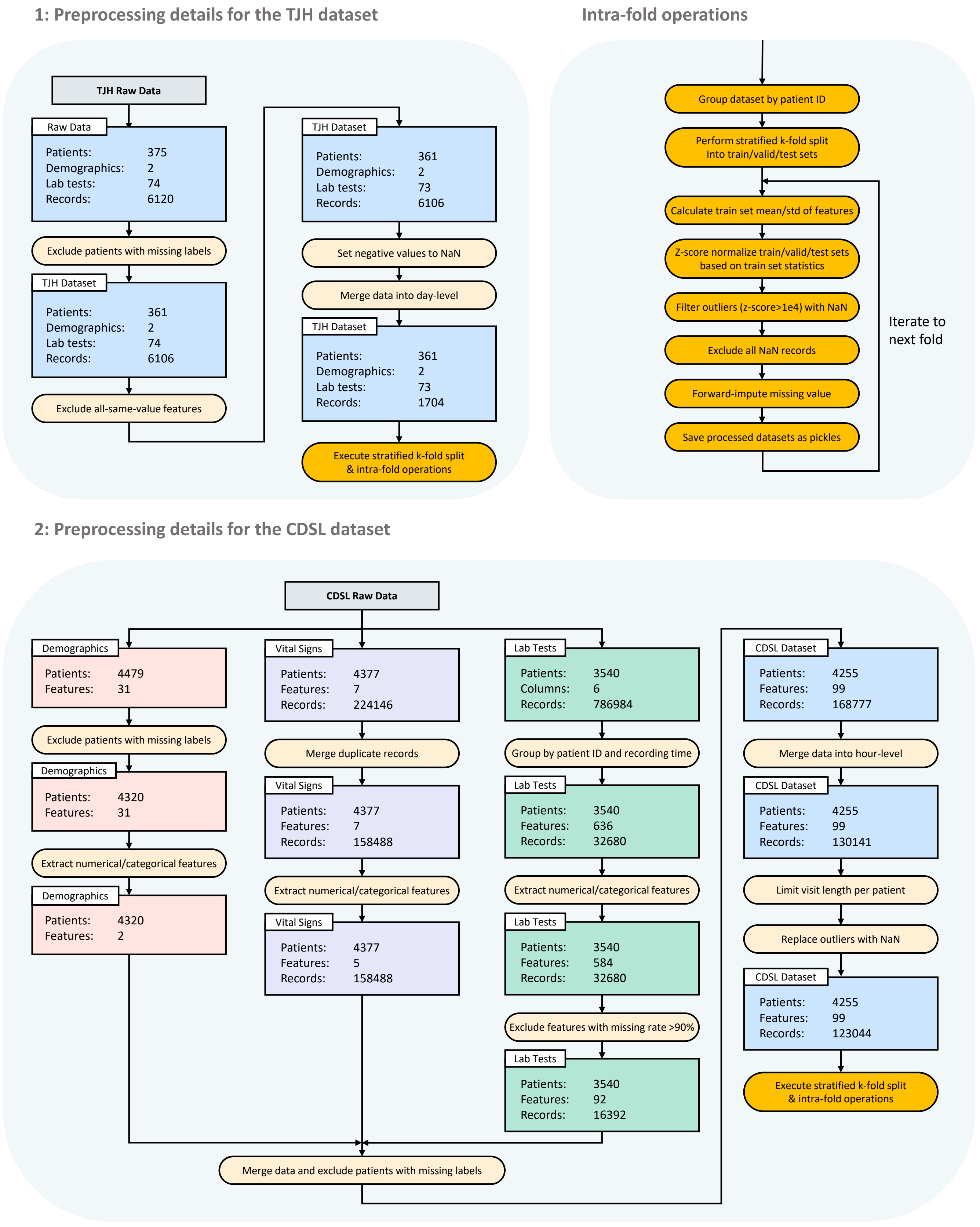

Figure S4: Data preprocessing details of two datasets.

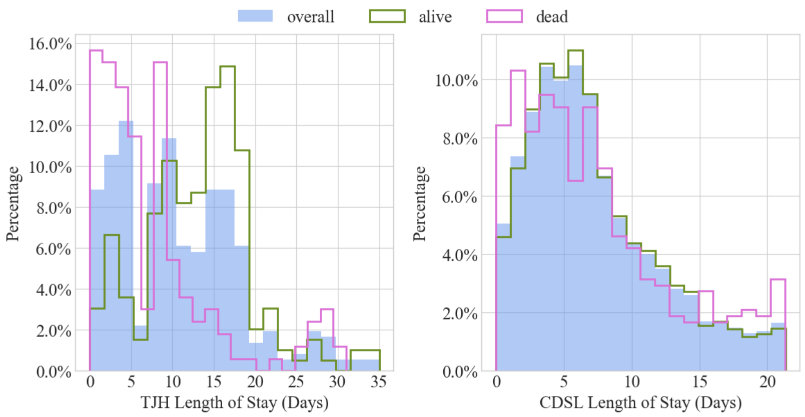

Figure S5: *Length of stay distributions in two datasets.* To keep the figure informative, we only show the statistics in the 0%-95% range for the CDSL dataset.

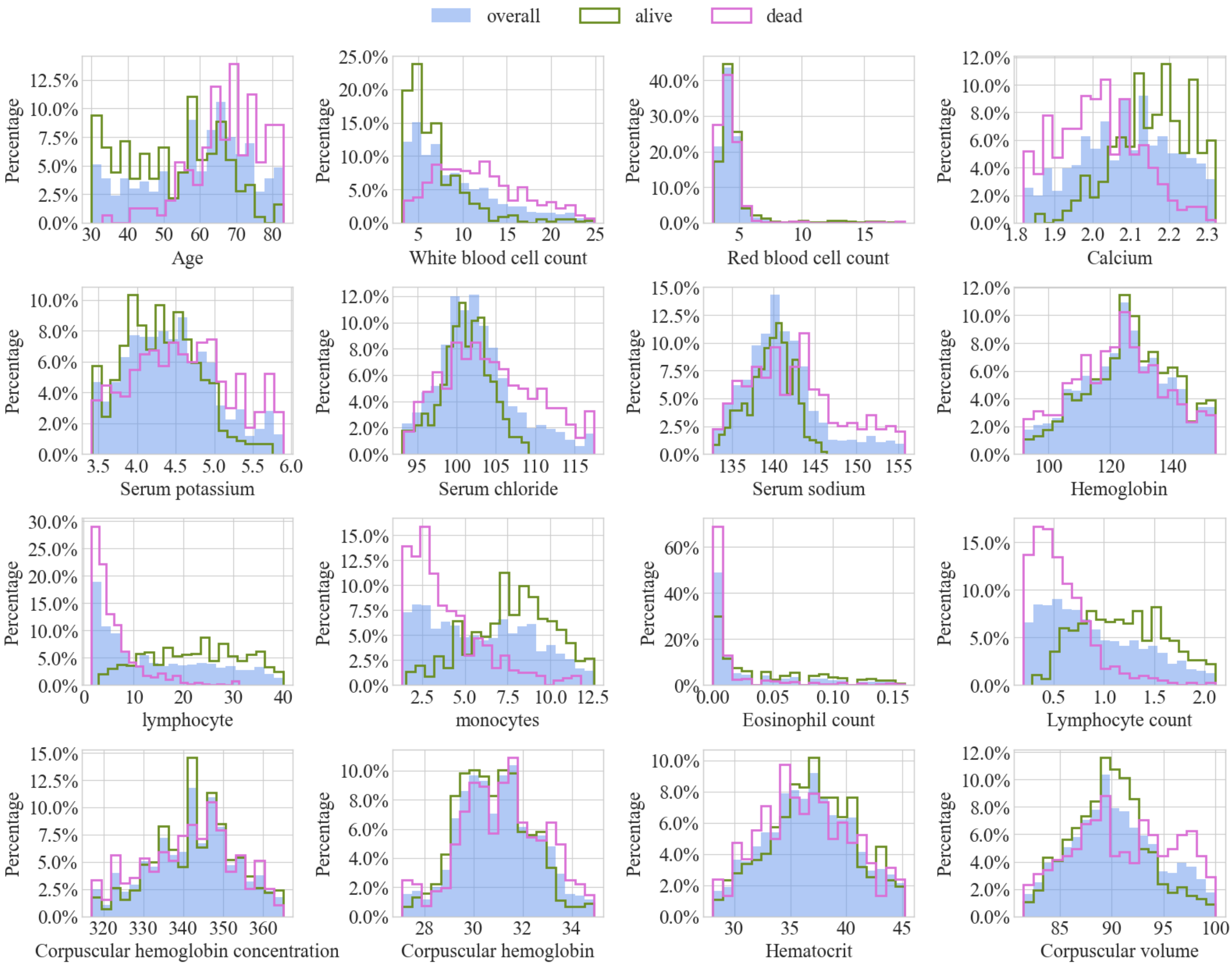

Figure S6: *Distributions of 16 features with the lowest missing rates in the TJH dataset.* We plot the data distributions of 16 features with the lowest missing rates for the overall, alive, and dead patients. The blue bars are distributions of total patients. The green and pink curves are distributions for alive and dead patients, respectively. To keep the figure informative, we only show the statistics in the 0%-95% range.

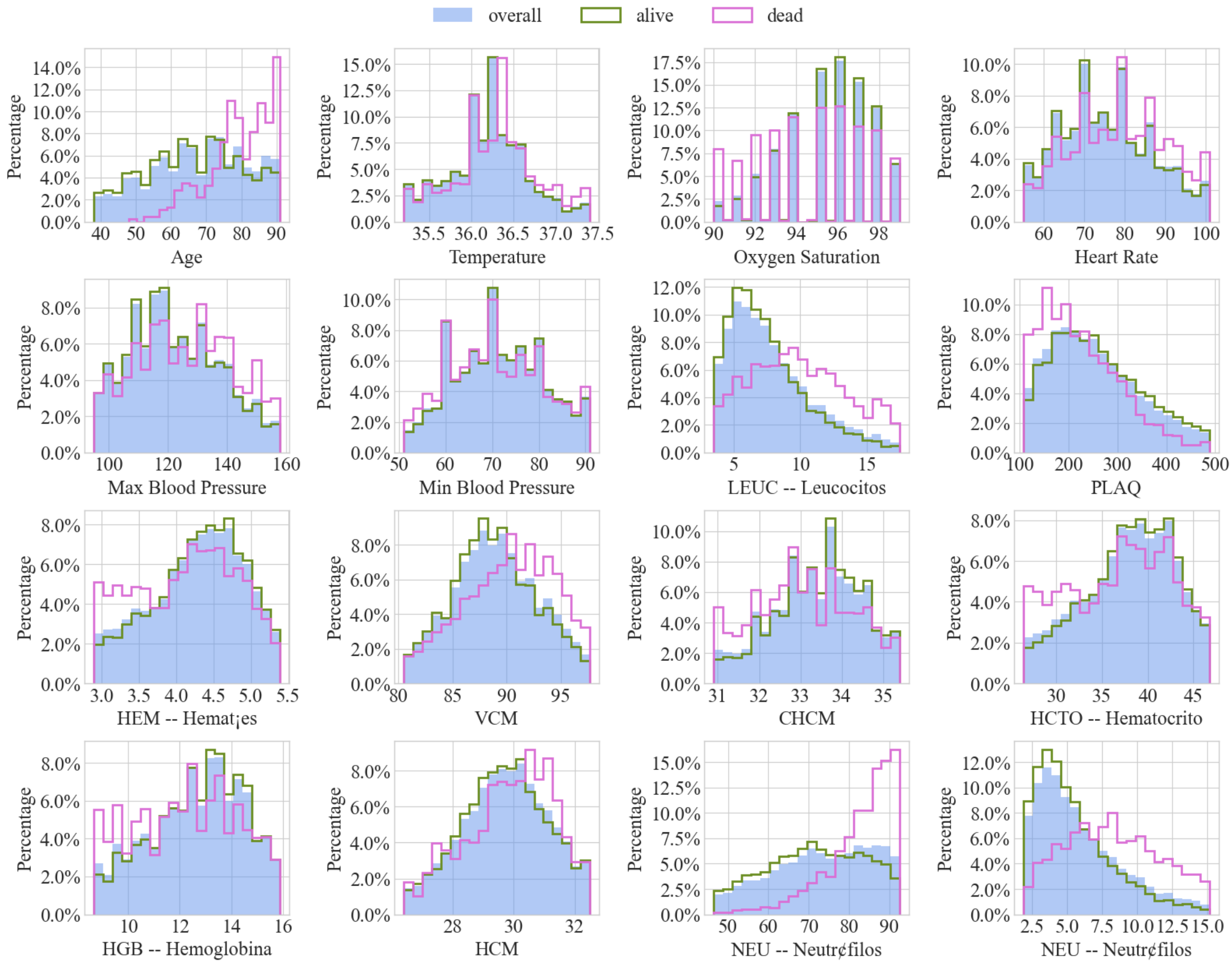

Figure S7: *Distributions of 16 features with the lowest missing rates in the CDSL dataset.* We plot the data distributions of 16 features with the lowest missing rates for the overall, alive, and dead patients. The blue bars are distributions of total patients. The green and pink curves are distributions for alive and dead patients, respectively. To keep the figure informative, we only show the statistics in the 0%-95% range.

Table S13: *Statistics of lab test features in the TJH dataset.* The reported statistics are of the form $Median[Q1, Q3]$.

Feature name	Statistics	Missing Rate
Hypersensitive cardiac troponinI	19.80 [4.20, 216.25]	29.23%
hemoglobin	125.00 [113.00, 137.00]	55.52%
Serum chloride	102.00 [98.95, 105.50]	55.58%
Prothrombin time	14.70 [13.60, 16.60]	38.44%
procalcitonin	0.10 [0.04, 0.40]	26.94%
eosinophils(%)	0.10 [0.00, 0.90]	55.40%
Interleukin 2 receptor	680.50 [460.00, 1169.75]	15.38%
Alkaline phosphatase	69.00 [54.00, 95.00]	53.81%
albumin	32.40 [27.50, 36.70]	53.99%
basophil(%)	0.20 [0.10, 0.30]	55.40%
Interleukin 10	5.80 [5.00, 12.50]	15.32%
Total bilirubin	10.60 [7.30, 16.70]	53.81%
Platelet count	181.00 [112.75, 250.25]	55.16%
monocytes(%)	5.70 [2.90, 8.70]	55.46%
antithrombin	86.00 [74.00, 97.00]	19.19%
Interleukin 8	15.95 [8.62, 33.58]	15.38%
indirect bilirubin	5.40 [3.80, 8.00]	52.46%
Red blood cell distribution width	12.60 [12.00, 13.60]	53.40%
neutrophils(%)	82.05 [65.07, 92.20]	55.40%
total protein	66.00 [61.10, 70.40]	53.81%
Treponema pallidum antibodies	0.05 [0.04, 0.07]	16.31%
Prothrombin activity	81.50 [65.00, 95.00]	38.26%
HBsAg	0.01 [0.00, 0.01]	16.31%
mean corpuscular volume	90.00 [86.90, 93.80]	55.40%
hematocrit	36.70 [33.50, 39.90]	55.40%
White blood cell count	7.60 [5.08, 12.52]	60.09%
Tumor necrosis factorα	8.60 [6.70, 11.58]	15.38%
corpuscular hemoglobin concentration	343.00 [333.75, 350.00]	55.40%
fibrinogen	4.12 [3.06, 5.51]	32.86%
Interleukin 1β	5.00 [5.00, 5.00]	15.38%
Urea	5.90 [4.00, 11.10]	53.99%
lymphocyte count	0.80 [0.47, 1.32]	55.40%
PH value	6.50 [6.00, 7.00]	21.01%
Red blood cell count	4.16 [3.67, 4.69]	60.09%
Eosinophil count	0.01 [0.00, 0.06]	55.40%
Corrected calcium	2.36 [2.27, 2.44]	52.82%
Serum potassium	4.41 [3.95, 4.86]	55.75%
glucose	6.98 [5.53, 10.15]	45.07%
neutrophils count	5.82 [3.08, 10.82]	55.40%
Direct bilirubin	4.80 [3.20, 8.00]	53.81%
Mean platelet volume	10.80 [10.10, 11.50]	50.12%
ferritin	711.60 [385.80, 1425.30]	16.49%
RBC distribution width SD	40.90 [38.50, 44.68]	53.40%
Thrombin time	16.80 [15.60, 18.30]	32.86%
lymphocyte(%)	11.70 [4.00, 25.00]	55.46%

Table S13: (Continued) Statistics of lab test features in the TJH dataset.

Feature name	Statistics	Missing Rate
HCV antibody quantification	0.06 [0.04, 0.09]	16.31%
D-D dimer	2.12 [0.60, 21.00]	36.74%
Total cholesterol	3.63 [3.01, 4.27]	53.87%
aspartate aminotransferase	27.00 [20.00, 42.00]	53.93%
Uric acid	244.00 [184.00, 332.60]	53.87%
HCO3-	23.50 [21.00, 25.90]	53.87%
calcium	2.09 [1.98, 2.19]	55.75%
NT-proBNP	571.50 [147.00, 2589.00]	27.58%
Lactate dehydrogenase	339.00 [217.00, 596.25]	53.87%
platelet large cell ratio	30.80 [25.50, 37.10]	50.12%
Interleukin 6	19.56 [4.66, 61.12]	15.61%
Fibrin degradation products	17.80 [4.00, 150.00]	19.19%
monocytes count	0.41 [0.27, 0.58]	55.40%
PLT distribution width	12.40 [11.10, 14.30]	50.12%
globulin	32.70 [29.70, 36.50]	53.81%
glutamyl transpeptidase	34.00 [22.00, 58.00]	53.81%
International standard ratio	1.14 [1.03, 1.33]	38.26%
basophil count(#)	0.01 [0.01, 0.02]	55.40%
mean corpuscular hemoglobin	30.90 [29.70, 32.20]	55.40%
Activation of partial thromboplastin time	39.20 [35.50, 44.10]	32.92%
Hypersensitive c-reactive protein	50.50 [5.35, 118.50]	42.66%
HIV antibody quantification	0.09 [0.07, 0.11]	16.26%
serum sodium	140.30 [137.70, 143.30]	55.58%
thrombocytocrit	0.21 [0.15, 0.27]	50.12%
ESR	28.00 [14.00, 45.50]	22.48%
glutamic-pyruvic transaminase	24.00 [16.00, 40.00]	53.87%
eGFR	88.10 [64.70, 104.20]	53.99%
creatinine	76.00 [58.00, 98.00]	53.99%

Table S14: *Statistics of lab test features in the CDSL dataset.* The reported statistics are of the form $Median[Q1, Q3]$.

Feature name	Statistics	Missing Rate
ADW – Coeficiente de anisocitosis	13.20 [12.10, 14.55]	8.34%
ADW – SISTEMATICO DE SANGRE	13.85 [12.30, 15.30]	2.68%
ALB – ALBUMINA	3.10 [2.70, 3.50]	1.27%
AMI – AMILASA	69.00 [46.92, 111.90]	1.25%
AP – ACTIVIDAD DE PROTROMBINA	79.00 [70.00, 87.00]	5.86%
APTT – TIEMPO DE CEFALINA (APTT)	30.80 [28.10, 33.70]	5.37%
AU – ACIDO URICO	4.70 [3.50, 6.50]	0.53%
BAS – Bas¢filos	0.02 [0.01, 0.04]	8.31%
BAS – SISTEMATICO DE SANGRE	0.02 [0.01, 0.04]	2.70%
BAS% – Bas¢filos %	0.30 [0.10, 0.50]	8.32%
BAS% – SISTEMATICO DE SANGRE	0.30 [0.10, 0.54]	2.70%
BD – BILIRRUBINA DIRECTA	0.26 [0.18, 0.40]	1.76%

Table S14: (Continued) Statistics of lab test features in the CDSL dataset.

Feature name	Statistics	Missing Rate
BE(b) – BE(b)	2.80 [0.00, 5.90]	2.57%
BE(b)V – BE (b)	1.90 [-0.60, 4.50]	1.00%
BEecf – BEecf	3.20 [-0.30, 6.78]	2.57%
BEecfV – BEecf	2.50 [-0.60, 5.40]	1.00%
BT – BILIRRUBINA TOTAL	0.40 [0.27, 0.57]	1.68%
BT – BILIRRUBINA TOTAL	0.52 [0.36, 0.77]	3.64%
CA – CALCIO	8.30 [8.00, 8.70]	0.77%
CA++ – Ca++ Gasometria	4.41 [4.21, 4.64]	2.30%
CHCM – Conc. Hemoglobina Corpuscular Media	33.40 [32.50, 34.20]	8.39%
CHCM – SISTEMATICO DE SANGRE	33.10 [32.10, 34.00]	2.70%
CK – CK (CREATINQUINASA)	66.10 [36.68, 140.00]	3.10%
CL – CLORO	102.30 [98.90, 106.43]	2.19%
CREA – CREATININA	0.82 [0.64, 1.06]	8.28%
DD – DIMERO D	985.00 [525.75, 2138.75]	6.31%
EOS – Eosin¢filos	0.03 [0.00, 0.13]	8.31%
EOS – SISTEMATICO DE SANGRE	0.10 [0.01, 0.23]	2.70%
EOS% – Eosin¢filos %	0.50 [0.00, 1.90]	8.32%
EOS% – SISTEMATICO DE SANGRE	1.30 [0.10, 3.20]	2.70%
FA – FOSFATASA ALCALINA	72.70 [55.50, 104.90]	3.29%
FER – FERRITINA	908.55 [445.92, 1645.00]	1.91%
FIB – FIBRINàGENO	562.00 [405.00, 724.00]	2.95%
FOS – FOSFORO	3.28 [2.70, 3.90]	2.15%
G-CORONAV (RT-PCR)	1.00 [0.00, 1.00]	0.49%
GGT – GGT	65.00 [30.00, 140.50]	5.53%
GLU – GLUCOSA	111.20 [94.00, 144.20]	7.67%
GOT – GOT (AST)	30.30 [20.80, 48.50]	7.42%
GPT – GPT (ALT)	32.60 [18.90, 61.00]	7.23%
HCM – Hemoglobina Corpuscular Media	29.80 [28.60, 30.80]	8.39%
HCM – SISTEMATICO DE SANGRE	29.90 [28.70, 30.90]	2.70%
HCO3 – HCO3-	27.40 [24.10, 31.10]	2.57%
HCO3V – HCO3-	27.20 [24.05, 29.90]	1.00%
HCTO – Hematocrito	38.30 [33.60, 42.00]	8.39%
HCTO – SISTEMATICO DE SANGRE	34.40 [29.60, 39.20]	2.70%
HEM – Hemat¡es	4.33 [3.75, 4.78]	8.39%
HEM – SISTEMATICO DE SANGRE	3.83 [3.25, 4.45]	2.70%
HGB – Hemoglobina	12.80 [11.10, 14.10]	8.39%
HGB – SISTEMATICO DE SANGRE	11.30 [9.70, 13.10]	2.70%
INR – INR	1.17 [1.09, 1.27]	5.87%
K – POTASIO	4.25 [3.86, 4.67]	8.10%
LAC – LACTATO	1.50 [1.10, 2.10]	2.46%
LDH – LDH	532.00 [410.05, 704.00]	7.29%
LEUC – Leucocitos	7.25 [5.34, 10.17]	8.39%
LEUC – SISTEMATICO DE SANGRE	7.70 [5.74, 10.85]	2.70%
LIN – Linfocitos	1.08 [0.72, 1.55]	8.39%
LIN – SISTEMATICO DE SANGRE	1.24 [0.79, 1.78]	2.70%
LIN% – Linfocitos %	15.50 [8.80, 24.50]	8.38%
LIN% – SISTEMATICO DE SANGRE	16.33 [9.06, 26.30]	2.70%

Table S14: (Continued) Statistics of lab test features in the CDSL dataset.

Feature name	Statistics	Missing Rate
MG – MAGNESIO	2.05 [1.81, 2.31]	2.40%
MONO – Monocitos	0.55 [0.36, 0.77]	8.32%
MONO – SISTEMATICO DE SANGRE	0.60 [0.42, 0.82]	2.70%
MONO% – Monocitos %	7.60 [4.80, 10.40]	8.32%
MONO% – SISTEMATICO DE SANGRE	7.80 [5.15, 10.30]	2.70%
NA – SODIO	138.00 [135.60, 140.70]	8.10%
NEU – Neutr¢filos	5.23 [3.46, 8.10]	8.39%
NEU – SISTEMATICO DE SANGRE	5.39 [3.53, 8.43]	2.70%
NEU% – Neutr¢filos %	74.10 [62.90, 84.60]	8.39%
NEU% – SISTEMATICO DE SANGRE	71.70 [59.20, 83.40]	2.70%
PCO2 – pCO2	41.50 [35.50, 48.50]	2.57%
PCO2V – pCO2	44.00 [38.00, 50.00]	1.00%
PCR – PROTEINA C REACTIVA	34.75 [8.72, 93.02]	8.03%
PH – pH	7.44 [7.39, 7.47]	2.57%
PHV – pH	7.40 [7.36, 7.43]	1.00%
PLAQ – Recuento de plaquetas	241.00 [176.00, 326.00]	8.39%
PLAQ – SISTEMATICO DE SANGRE	238.00 [174.00, 315.00]	2.70%
PO2 – pO2	82.80 [63.00, 106.00]	2.57%
PO2V – pO2	42.00 [27.00, 59.00]	1.00%
PROCAL – PROCALCITONINA	0.14 [0.08, 0.30]	0.77%
PT – PROTEINAS TOTALES	5.62 [5.10, 6.10]	0.84%
SO2C – sO2c (Saturaci¢n de ox¡geno)	95.67 [92.00, 97.67]	2.57%
SO2CV – sO2c (Saturaci¢n de ox¡geno)	76.00 [48.00, 90.00]	1.00%
TCO2 – tCO2(B)c	28.70 [25.20, 32.53]	2.57%
TCO2V – tCO2 (B)	28.60 [25.10, 31.49]	1.00%
TP – TIEMPO DE PROTROMBINA	13.00 [12.10, 14.10]	5.86%
TROPO – TROPONINA	15.16 [7.95, 34.92]	0.99%
U – UREA	41.60 [29.00, 61.10]	8.06%
VCM – SISTEMATICO DE SANGRE	90.00 [87.00, 93.40]	2.70%
VCM – Volumen Corpuscular Medio	88.90 [86.00, 92.30]	8.39%
VPM – SISTEMATICO DE SANGRE	10.50 [9.80, 11.30]	2.67%
VPM – Volumen plaquetar medio	10.40 [9.70, 11.10]	8.30%
VSG – VSG	47.50 [16.00, 75.00]	0.47%

References

1. Knight, S. R., Ho, A., Pius, R., Buchan, I., Carson, G., Drake, T. M., Dunning, J., Fairfield, C. J., Gamble, C., Green, C. A. et al. (2020). Risk stratification of patients admitted to hospital with covid-19 using the isaric who clinical characterisation protocol: development and validation of the 4c mortality score. bmj *370*.

2. Noh, J., Yoo, K. D., Bae, W., Lee, J. S., Kim, K., Cho, J.-H., Lee, H., Kim, D. K., Lim, C. S., Kang, S.-W. et al. (2020). Prediction of the mortality risk in peritoneal dialysis patients using machine learning models: a nation-wide prospective cohort in korea. Scientific reports *10*, 1–11.

3. Iwendi, C., Bashir, A. K., Peshkar, A., Sujatha, R., Chatterjee, J. M., Pasupuleti, S., Mishra, R., Pillai, S., and Jo, O. (2020). Covid-19 patient health prediction using boosted random forest algorithm. Frontiers in public health *8*, 357.

4. Li, S., Lin, Y., Zhu, T., Fan, M., Xu, S., Qiu, W., Chen, C., Li, L., Wang, Y., Yan, J. et al. (2021). Development and external evaluation of predictions models for mortality of covid-19 patients using machine learning method. Neural Computing and Applications (1–10).

5. Chen, T., and Guestrin, C. Xgboost: A scalable tree boosting system. In: *Proceedings of the 22nd acm sigkdd international conference on knowledge discovery and data mining* (2016):(785–794).

6. Yan, L., Zhang, H.-T., Goncalves, J., Xiao, Y., Wang, M., Guo, Y., Sun, C., Tang, X., Jing, L., Zhang, M. et al. (2020). An interpretable mortality prediction model for covid-19 patients. Nature machine intelligence *2*, 283–288.

7. Dorogush, A. V., Ershov, V., and Gulin, A. (2018). Catboost: gradient boosting with categorical features support. arXiv preprint arXiv:1810.11363. doi:`10.48550/arXiv.1810.11363`.

8. Kim, J. S. (2021). Covid-19 prediction and detection using machine learning algorithms: Catboost and linear regression. American Journal of Theoretical and Applied Statistics *10*, 208–215.

9. Tomašev, N., Glorot, X., Rae, J. W., Zielinski, M., Askham, H., Saraiva, A., Mottram, A., Meyer, C., Ravuri, S., Protsyuk, I. et al. (2019). A clinically applicable approach to continuous prediction of future acute kidney injury. Nature *572*, 116–119.

10. Rumelhart, D. E., Hinton, G. E., and Williams, R. J. (1986). Learning representations by back-propagating errors. nature *323*, 533–536.

11. Raket, L. L., Jaskolowski, J., Kinon, B. J., Brasen, J. C., Jönsson, L., Wehnert, A., and Fusar-Poli, P. (2020). Dynamic electronic health record detection (detect) of individuals at risk of a first episode of psychosis: a case-control development and validation study. The Lancet Digital Health *2*, e229—e239.

12. Choi, E., Bahadori, M. T., Schuetz, A., Stewart, W. F., and Sun, J. Doctor ai: Predicting clinical events via recurrent neural networks. In: *Machine learning for healthcare conference*. PMLR (2016):(301–318).

13. Hochreiter, S., and Schmidhuber, J. (1997). Long short-term memory. Neural computation *9*, 1735–1780.

14. Thorsen-Meyer, H.-C., Nielsen, A. B., Nielsen, A. P., Kaas-Hansen, B. S., Toft, P., Schierbeck, J., Strøm, T., Chmura, P. J., Heimann, M., Dybdahl, L. et al. (2020). Dynamic and explainable machine learning prediction of mortality in patients in the intensive care unit: a retrospective study of high-frequency data in electronic patient records. The Lancet Digital Health *2*, e179–e191.

15. Chung, J., Gulcehre, C., Cho, K., and Bengio, Y. (2014). Empirical evaluation of gated recurrent neural networks on sequence modeling. arXiv preprint arXiv:1412.3555. doi:`10.48550/arXiv.1412.3555`.

16. Meyer, A., Zverinski, D., Pfahringer, B., Kempfert, J., Kuehne, T., Sündermann, S. H., Stamm, C., Hofmann, T., Falk, V., and Eickhoff, C. (2018). Machine learning for real-time prediction of complications in critical care: a retrospective study. The Lancet Respiratory Medicine *6*, 905–914.

17. Bai, S., Kolter, J. Z., and Koltun, V. (2018). An empirical evaluation of generic convolutional and recurrent networks for sequence modeling. arXiv preprint arXiv:1803.01271. doi:`10.48550/arXiv.1803.01271`.

18. Zhang, J., Pathak, H. S., Snowdon, A., and Greiner, R. (2022). Learning models for forecasting hospital resource utilization for covid-19 patients in canada. Scientific reports *12*, 1–14.

19. Vaswani, A., Shazeer, N., Parmar, N., Uszkoreit, J., Jones, L., Gomez, A. N., Kaiser, Ł., and Polosukhin, I. (2017). Attention is all you need. Advances in neural information processing systems *30*.

20. Nitski, O., Azhie, A., Qazi-Arisar, F. A., Wang, X., Ma, S., Lilly, L., Watt, K. D., Levitsky, J., Asrani, S. K., Lee, D. S. et al. (2021). Long-term mortality risk stratification of liver transplant recipients: real-time application of deep learning algorithms on longitudinal data. The Lancet Digital Health *3*, e295–e305.

21. Choi, E., Bahadori, M. T., Sun, J., Kulas, J., Schuetz, A., and Stewart, W. (2016). Retain: An interpretable predictive model for healthcare using reverse time attention mechanism. Advances in neural information processing systems *29*.

22. Gao, J., Xiao, C., Wang, Y., Tang, W., Glass, L. M., and Sun, J. Stagenet: Stage-aware neural networks for health risk prediction. In: *Proceedings of The Web Conference 2020* (2020):(530–540).

23. Gao, J., Xiao, C., Glass, L. M., and Sun, J. (2020). Dr. agent: Clinical predictive model via mimicked second opinions. Journal of the American Medical Informatics Association *27*, 1084–1091.

24. Ma, L., Gao, J., Wang, Y., Zhang, C., Wang, J., Ruan, W., Tang, W., Gao, X., and Ma, X. (2020). Adacare: Explainable clinical health status representation learning via scale-adaptive feature extraction and recalibration. Proceedings of the AAAI Conference on Artificial Intelligence *34*, 825–832. URL: `https://ojs.aaai.org/index.php/AAAI/article/view/5427`. doi:`10.1609/aaai.v34i01.5427`.

25. Ma, L., Zhang, C., Wang, Y., Ruan, W., Wang, J., Tang, W., Ma, X., Gao, X., and Gao, J. (2020). Concare: Personalized clinical feature embedding via capturing the healthcare context. Proceedings of the AAAI Conference on Artificial Intelligence *34*, 833–840. URL: `https://ojs.aaai.org/index.php/AAAI/article/view/5428`. doi:`10.1609/aaai.v34i01.5428`.

26. Zhang, C., Gao, X., Ma, L., Wang, Y., Wang, J., and Tang, W. (2021). Grasp: Generic framework for health status representation learning based on incorporating knowledge from similar patients. Proceedings of the AAAI Conference on Artificial Intelligence *35*, 715–723. URL: https://ojs.aaai.org/index.php/AAAI/article/view/16152. doi:10.1609/aaai.v35i1.16152.

27. Feldman, M., Friedler, S. A., Moeller, J., Scheidegger, C., and Venkatasubramanian, S. Certifying and removing disparate impact. In: *proceedings of the 21th ACM SIGKDD international conference on knowledge discovery and data mining* (2015):(259–268).

28. Hardt, M., Price, E., and Srebro, N. (2016). Equality of opportunity in supervised learning. Advances in neural information processing systems *29*.

29. Calders, T., and Verwer, S. (2010). Three naive bayes approaches for discrimination-free classification. Data mining and knowledge discovery *21*, 277–292.

30. Johnson, A. E., Pollard, T. J., Shen, L., Lehman, L.-w. H., Feng, M., Ghassemi, M., Moody, B., Szolovits, P., Anthony Celi, L., and Mark, R. G. (2016). Mimic-iii, a freely accessible critical care database. Scientific data *3*, 1–9.

31. Johnson, A., Bulgarelli, L., Pollard, T., Horng, S., Celi, L. A., and Mark, R. (2020). Mimic-iv. PhysioNet. Available online at: https://physionet. org/content/mimiciv/1.0/(accessed August 23, 2021).

32. Harutyunyan, H., Khachatrian, H., Kale, D. C., Ver Steeg, G., and Galstyan, A. (2019). Multitask learning and benchmarking with clinical time series data. Scientific data *6*, 1–18.

33. Dwivedi, A. K., Mallawaarachchi, I., and Alvarado, L. A. (2017). Analysis of small sample size studies using nonparametric bootstrap test with pooled resampling method. Statistics in medicine *36*, 2187–2205.
