# Supplementary material for: A comprehensive benchmark for COVID-19 predictive modeling using electronic health records in intensive care
Source: Patterns (N Y). 2024 Mar 7;5(4):100951. doi: 10.1016/j.patter.2024.100951 (PMC11026964; doi:10.1016/j.patter.2024.100951)
Supplement: Document S2. Article plus supplemental information [file mmc2.pdf]

Article

Patterns

A comprehensive benchmark for COVID-19 predictive modeling using electronic health records in intensive care

Graphical abstract

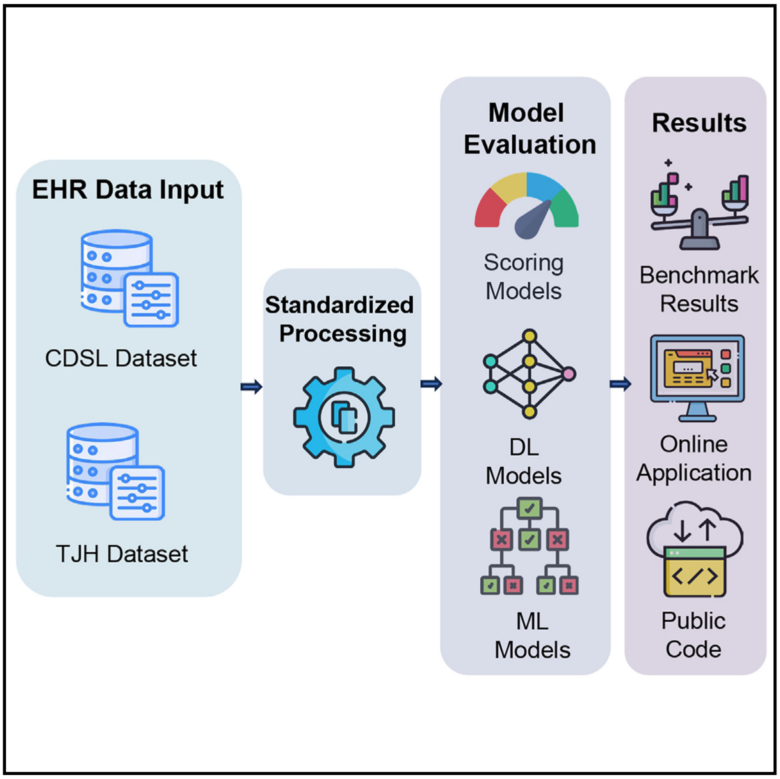

Highlights

- Introduced two predictive tasks for COVID-19 ICU care
- Benchmarking 18 models with real-world data for clinical use
- Open-source data preprocessing framework and online access to models and results

Authors

Junyi Gao, Yinghao Zhu, Wenqing Wang, ..., Yasha Wang, Ewen M. Harrison, Liantao Ma

Correspondence

malt@pku.edu.cn

In brief

In this study, researchers tackle the challenges of predicting health-care outcomes during pandemics like COVID-19. They introduce innovative prediction tasks specifically for intensive care patients, evaluating a wide range of models from traditional to advanced deep learning. The study not only benchmarks these models using real-world data but also makes the results and tools publicly available. This work is significant, as it advances the use of deep learning in health care, offering valuable resources for both clinicians and researchers.

Gao et al., 2024, Patterns *5*, 100951
April 12, 2024 © 2024 The Author(s).
https://doi.org/10.1016/j.patter.2024.100951

CellPress

Patterns

CellPress
OPEN ACCESS

Article

A comprehensive benchmark for COVID-19 predictive modeling using electronic health records in intensive care

Junyi Gao,[2,3,6] Yinghao Zhu,[1,6] Wenqing Wang,[1,6] Zixiang Wang,[1] Guiying Dong,[4] Wen Tang,[5] Hao Wang,[1] Yasha Wang,[1] Ewen M. Harrison,[2] and Liantao Ma[1,7,*]
[1]Peking University, Beijing 100871, China
[2]Centre for Medical Informatics, University of Edinburgh, EH16 4UX Edinburgh, UK
[3]Health Data Research UK, NW1 2BE London, UK
[4]Peking University People's Hospital, Beijing 100044, China
[5]Peking University Third Hospital, Beijing 100191, China
[6]These authors contributed equally
[7]Lead contact
*Correspondence: malt@pku.edu.cn
https://doi.org/10.1016/j.patter.2024.100951

THE BIGGER PICTURE Predictive learning models aim to understand data and retrieve information about future outcomes. In medical settings, these models have enormous potential to predict relevant information, for example, the length of hospital stays, that can in turn help medical providers establish adequate treatment and patient care strategies. The COVID-19 pandemic revealed the importance of applying predictive models in health care. Studies evaluating multiple models and tasks that reflect the reality of clinical settings with real-world health data are critical for developing better and more precise frameworks for research in patient-level pandemic predictive modeling.

SUMMARY

The COVID-19 pandemic highlighted the need for predictive deep-learning models in health care. However, practical prediction task design, fair comparison, and model selection for clinical applications remain a challenge. To address this, we introduce and evaluate two new prediction tasks—outcome-specific length-of-stay and early-mortality prediction for COVID-19 patients in intensive care—which better reflect clinical realities. We developed evaluation metrics, model adaptation designs, and open-source data preprocessing pipelines for these tasks while also evaluating 18 predictive models, including clinical scoring methods and traditional machine-learning, basic deep-learning, and advanced deep-learning models, tailored for electronic health record (EHR) data. Benchmarking results from two real-world COVID-19 EHR datasets are provided, and all results and trained models have been released on an online platform for use by clinicians and researchers. Our efforts contribute to the advancement of deep-learning and machine-learning research in pandemic predictive modeling.

INTRODUCTION

The COVID-19 pandemic has undoubtedly left an indelible impact on the global community. Although recent studies suggest that new COVID-19 variants are less lethal, their heightened transmissibility contributes to a continual surge in cases worldwide.[1] Given these circumstances, the critical need for early risk prediction and disease progression estimation, especially for COVID-19 patients in intensive care units (ICUs), is essential to optimally allocate medical resources and alleviate the strain on our health-care system in the post-pandemic era.

Despite the commendable performance of existing COVID-19 predictive modeling works on specific datasets,[2–10] researchers and clinicians often encounter difficulties when attempting to apply state-of-the-art prediction models to new data. Questions arise regarding whether to employ deep-learning (DL) models or traditional machine-learning (ML) models and how different models compare in terms of prediction performance. Although there are a few descriptive review works,[11,12] there is still a

This is an open access article under the CC BY license (http://creativecommons.org/licenses/by/4.0/).

lack of a fair quantitative comparative framework for existing models. Various models have been applied to different datasets, many of which either are not publicly available or entail strict access restrictions. Direct comparison of their results is therefore problematic, and model selection for new data is equally challenging, as re-implementing all existing models on new data is resource intensive, particularly for clinicians. This limitation curtails the practical usage of these models and hinders further research, underlining the necessity for a benchmark for COVID-19 predictive modeling that facilitates model comparison using identical data and evaluation strategies.

Existing electronic health record (EHR) prediction benchmarks[13–16] predominantly rely on publicly available ICU datasets, such as MIMIC-III[17] and MIMIC-II.[18] These benchmarks compare various machine-learning and deep-learning models across multiple standardized prediction tasks, including mortality prediction, patient phenotyping, length-of-stay (LOS) prediction, etc. However, these efforts primarily evaluate the performance of basic machine-learning and deep-learning models like recurrent neural networks (RNNs) or long-short-term memory networks (LSTM), despite the presence of numerous advanced deep-learning models specifically designed for EHR data and clinical tasks. Notably, comprehensive benchmarking results for machine-learning, deep-learning, and more advanced models on publicly available datasets remain absent for COVID-19 predictive tasks.

Another concern lies in the fact that current COVID-19 prediction works largely mirror the task settings of previous EHR data-mining endeavors. Risk or mortality prediction models, for instance, typically output the predicted risk score at the final time step of available EHR sequences.[2,10] This methodology can be problematic as it may be too late to initiate life-saving treatments for high-risk COVID-19 patients (especially ICU patients) by the time their status at the last time step is identified as critical.[19,20] Simultaneously, the LOS prediction task is commonly formulated as a regression task,[15] with the model outputting the predicted remaining LOS at each time step. This approach presents a notable issue where high-risk and low-risk patients may exhibit the same LOS value, as both death and ICU discharge are defined as the end of the stay in previous benchmarking works.[14,15,21,22] Although the model can theoretically learn non-linearity and map these statuses to distinct locations in the latent embedding space, the practical application may prove challenging for clinicians. Adaptations are thus required for both tasks to cater to the unique intensive care setting associated with COVID-19. Consequently, it is imperative to compare existing deep-learning and machine-learning models using more clinically practical prediction tasks, identical publicly available data, and consistent evaluation settings.

In this study, we aim to bridge these gaps by proposing a standardized and comprehensive benchmark for COVID-19 prediction tasks in ICUs. This benchmark enables comparison among machine-learning, basic deep-learning, and state-of-the-art EHR-specific models. Our contributions are 3-fold:

(1) Task, metric, and model designing. We introduce two tasks based on the clinical practice for COVID-19 patients in ICUs: outcome-specific LOS prediction and early-mortality prediction. Distinct from existing LOS-prediction frameworks, the outcome-specific LOS prediction is formulated as a multitarget task. This task simultaneously outputs the patient outcome and the corresponding LOS, enabling clinicians to distinguish progression status between low-risk and high-risk patients. The early-mortality prediction task is designed to alert clinicians to high-risk patients as early as possible, thereby preempting potential treatment delays. We design specific evaluation metrics for both tasks, namely the early-prediction score (ES) and the outcome-specific mean absolute error (OSMAE), to assess model performance. We have also designed a multitask training architecture for the LOS-prediction task and a time-aware (TA) loss term that can significantly improve deep-learning models' early-prediction performance.

(2) Data preparation and preprocessing pipelines. We have established data preprocessing pipelines that include cleaning, filtering, missing-value imputation, and cohort construction for two real-world COVID-19 EHR datasets. Both datasets consist of heterogeneous longitudinal EHR data from ICUs. Features include lab tests, vital signs, diagnoses, and static demographic information.

(3) Modeling and benchmarking. We have implemented and evaluated 18 state-of-the-art predictive models across the two tasks, including one clinical scoring method, five machine-learning models, six basic deep-learning models, and six deep-learning predictive models specifically designed for multivariate time-series EHR data. We conducted fair and reproducible comparisons and provide detailed benchmarking results to foster further research in this field.

We have made our code publicly available, enabling others to build complete benchmarks and reproduce all results. Our well-structured data preprocessing and modeling modules can also be easily applied to generate customized tasks and results. The benchmark code and documentations can be accessed at Zenodo: https://doi.org/10.5281/zenodo.10573567.[23] Moreover, we have released all the benchmark experiment results and trained models on an online platform, which includes model performances with all hyperparameter combinations for both tasks and makes the results easy to query and download. The platform can be accessed at https://pyehr.netlify.app.[24].

RESULTS

Dataset description and problem formulation

The datasets used in this study and the proposed prediction tasks are as follows.

EHR datasets for COVID-19 patients in intensive care

In this work, we utilize two COVID-19 EHR datasets to conduct benchmark experiments. After performing an exhaustive search for publicly available COVID-19 EHR datasets worldwide, we selected two datasets based on ease of access and absence of regional restrictions:

Dataset 1 (Tongji Hospital COVID-19 dataset [TJH])[2] comprises anonymized EHR data from 485 COVID-19 patients admitted to Tongji Hospital, China, between January 10 and

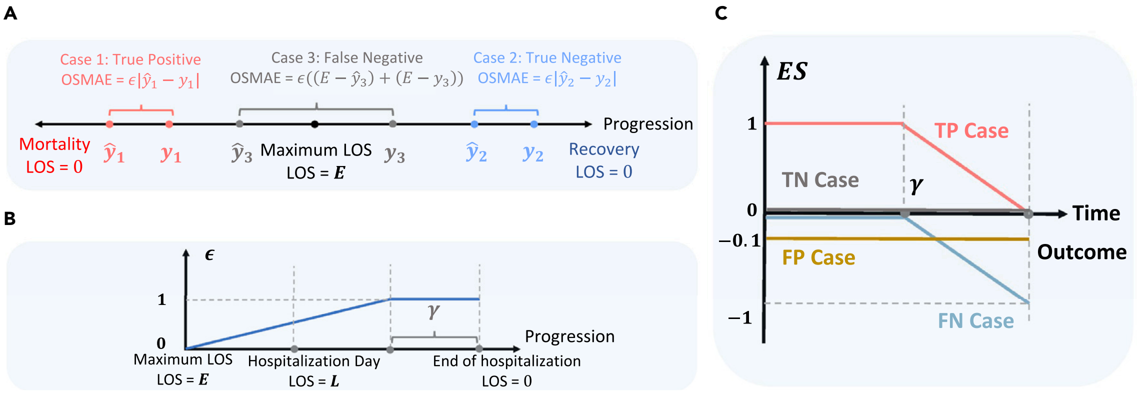

Figure 1. Illustrations of the proposed metrics

(A) Illustration of the outcome-specific mean absolute error (OSMAE).

(B) Illustration of the penalty term ϵ.

(C) Illustration of the early-prediction score (ES).

February 24, 2020. The dataset includes 74 lab tests and vital signs, all of which are numerical features, as well as two demographic features (age and gender). This dataset is publicly accessible via GitHub (https://github.com/HAIRLAB/Pre_Surv_COVID_19). The dataset download script is included in our benchmark code.

Dataset 2 (HM Hospitales COVID Data Save Lives [CDSL])[25] originates from the HM Hospitales EHR system and contains anonymized records of 4,479 patients admitted with a diagnosis of COVID-19 or suspected COVID-19 infection. The dataset includes heterogeneous medical features such as detailed information on diagnoses, treatments, admissions, ICU admissions, diagnostic imaging tests, laboratory results, and patient discharge or death status. The dataset is open to global researchers and can be accessed upon request (https://www.hmhospitales.com/prensa/notas-de-prensa/comunicado-covid-data-save-lives). Prospective users must complete a request form.

These datasets have been utilized in several mortality prediction studies.[2,26] However, we found no existing well-organized data preprocessing pipeline code or benchmarking results for them, which prompted us to provide a comprehensive benchmark analysis and data-processing suite for these publicly available datasets, thereby facilitating related research.

Problem formulation and evaluation metrics

In this study, we formulate two tasks—outcome-specific LOS prediction and early-mortality prediction—to evaluate and compare the performances of various machine-learning and deep-learning models. These tasks, which are adaptations of common LOS- and mortality-prediction models, are tailored to suit the requirements of COVID-19 intensive care settings.

Problem 1 (outcome-specific length-of-stay prediction). LOS prediction is an important task in clinical practice, which better facilitates clinical resource management. We define outcome-specific LOS prediction as a multitarget prediction task, which encompasses a binary outcome classification task and an LOS regression task. At each time step t, the model generates two outputs: a predicted outcome $\hat{y}_{m,t} \in \{0,1\}$ (i.e., 1 for mortality and 0 for survival) and a predicted LOS $\hat{y}_{l,t} \geq 0$ indicating the remaining days corresponding to the predicted outcome or the end of the ICU stay. Compared with traditional LOS-prediction models that output only $\hat{y}_{l,t}$, our model provides a more comprehensive evaluation of a patient's progression, allowing clinicians to differentiate LOS according to different outcomes. The model's learning process may also benefit from this setting, as patients with varying health statuses are explicitly modeled in the latent space.

We use mean absolute error (MAE) and mean squared error (MSE) as evaluation metrics. Furthermore, to evaluate this multitarget task comprehensively, we propose a new metric, OSMAE, formulated as follows:

$$OSMAE = \begin{cases} \epsilon(max(E - \hat{y}_l, 0) + max(E - y_l, 0)) & , \hat{y}_m \neq y_m \\ \epsilon|\hat{y}_l - y_l| & , \hat{y}_m = y_m \end{cases}$$

$$\epsilon = \begin{cases} \dfrac{t}{E - L + \gamma}, & 0 < t \leq L - \gamma \\ 1, & t > L - \gamma \end{cases}.$$

Here, t represents the time step, L is the total LOS, and E is the maximum value at the 95th percentile of the LOS of all patients. ϵ is a penalty term represented by a piecewise function. A lower OSMAE indicates better model performance. Excluding the penalty term ϵ, OSMAE is equivalent to the conventional MAE for true-positive and true-negative predictions, i.e., $\hat{y}_m = y_m$. However, for false-positive and false-negative patients, OSMAE is significantly higher than the original MAE, since the model's prediction for the patient's outcome deviates completely from the actual outcome. We introduce the penalty term to prevent the model from incurring a high OSMAE during the early time steps, as a patient's status may be uncertain during the initial ICU-stay phase. γ serves as a penalty threshold, indicating that we expect the model to make accurate predictions within the final γ days. The default value of γ is $0.5 * \bar{y}_{l,0}$, where $\bar{y}_{l,0}$ is the average value of all patients' total LOS. We provide a sensitivity analysis for γ in the discussion. Figure 1 illustrates the calculation of OSMAE and the penalty term using three examples: true positive, false negative, and true negative.

Problem 2 (early-mortality prediction). Early-mortality prediction is defined as a binary sequential prediction task. For each

Table 1. Statistics of the TJH dataset

Mortality outcome	Total	Alive	Dead
No. of patients	361	195 (54.02%)	166 (45.98%)
No. of records	1,704	1,050 (61.62%)	654 (38.38%)
Average number of records	5.0 [3, 6]	5.0 [4, 7]	3.0 [2, 5]
Age (years)	62.0 [46.0, 70.0]	51.0 [37.0, 62.0]	69.0 [62.25, 77.0]
>Average (58)	205 (56.79%)	68 (34.87%)	137 (82.53%)
≤Average (58)	156 (43.21%)	127 (65.13%)	29 (17.47%)
Gender	58.7% male	47.2% male	72.3% male
Male	212 (58.73%)	92 (47.18%)	120 (72.29%)
Female	149 (41.27%)	103 (52.82%)	46 (27.71%)
No. of features	75	–	–
Length of stay (days)	10.0 [4.0, 15.0]	14.0 [9.0, 17.0]	5.0 [3.0, 10.0]

The reported statistics are of the form median [Q1, Q3].

time step in the longitudinal EHR input sequence, the model will predict the mortality risk $\hat{y}_r \in \{0, 1\}$ for this ICU stay, signifying whether the patient will succumb by the end of the ICU stay. An optimal early-prediction model should raise an alert as soon as possible during the stay. Many existing mortality-prediction models feed the entire EHR sequence into the model and predict the mortality risk within a short future time window (e.g., 24 or 12 h).[15,16,27,28] However, this approach restricts the clinical applicability of these models. Particularly for COVID-19 patients in the ICU, their health status may have severely deteriorated by the final time step, making the risks evident to clinicians. At this advanced stage, an alarm indicating high risk may be too late to initiate life-saving treatments or procedures. To address this limitation, we propose to assess early-prediction performance using weighted metrics.

Table 2. Statistics of the CDSL dataset

Mortality outcome	Total	Alive	Dead
No of. patients	4,255	3,715 (87.31%)	540 (12.69%)
No. of records	123,044	108,142 (87.89%)	14,902 (12.11%)
Average no. of records	24.0 [15, 39]	25.0 [15, 39]	22.5 [11, 37]
Age (years)	67.2 [56.0, 80.0]	65.1 [54.0, 77.0]	81.6 [75.0, 89.0]
>Average (67)	2,228 (52.36%)	1,748 (47.05%)	480 (88.89%)
≤Average (58)	2,027 (47.64%)	1,967 (52.95%)	60 (11.11%)
Gender	59.1% male	58.5% male	63.3% male
Male	2,515 (59.11%)	2,173 (58.49%)	342 (63.33%)
Female	1,740 (40.89%)	1,542 (41.51%)	198 (36.67%)
No. of features	99	–	–
Length of stay (days)	6.4 [4.0, 11.0]	6.1 [4.0, 11.0]	6.0 [3.0, 10.0]

The reported statistics are of the form median [Q1, Q3].

To evaluate models' early-prediction performance, we not only utilize traditional measures such as area under the receiver operating characteristic (AUROC) and area under the precision-recall curve (AUPRC), but we also introduce the early-prediction score (ES) to specifically assess the early-prediction performance of the models. Figure 1C illustrates the ES. Drawing from previous studies,[20] the idea is to assign a full score to early true-positive predictions while penalizing late false-negative predictions, i.e., the model fails to predict patients' outcomes accurately even in the final moments. We also give false alarms a small penalty (−0.1). Different penalty scores will not affect the relative comparison between different models. We provide details in the discussion. We normalize the total ES for one patient as follows:

$$ES_{normalized} = \max\left(ES_{total} / ES_{optimal}, -1\right).$$

This normalization ensures that the ground truth (i.e., the highest possible score) receives a normalized score of 1 and the worst algorithm, which outputs only negative predictions, receives a normalized score of −1.

Pipeline design

In this section, we introduce our benchmarking pipeline design, including data preprocessing, baseline selection, and training and evaluation strategies.

Data preprocessing

Our benchmark data processing pipeline comprises four stages: data cleaning, merging, normalization, and imputation. Although both datasets are well-formatted data tables, they still contain artifacts that necessitate preprocessing. Previous machine-learning and deep-learning works on COVID-19 predictive modeling lack a uniform setting for these preprocessing details, leading to subtle performance differences. In this study, we propose a reproducible and reliable preprocessing pipeline to establish a fair comparison base for various models.

Data cleaning and merging

Our first step in preprocessing the dataset into feature matrices is to structure the EHR data format for use in machine-learning or deep-learning models. Each patient's longitudinal EHR records are represented as a matrix, with each column representing a specific feature or label and each row representing a record. We also extract demographic information for each patient as static data. During this step, we calculate ground-truth labels, such as LOS and mortality outcome.

For error-record cleaning, we first compute each feature's statistics, including the mean, standard deviation, minimum, maximum, median, and missing rate. For instance, in the TJH dataset, we observed that all entries for "2019-nCoV nucleic acid detection" are identical, and some other feature values are negative. We removed these clear error records. For example, in the CDSL dataset, we removed obvious errors such as oxygen saturation values above 100 and maximum blood pressure exceeding 220. All filtered values are replaced with NaN (not a number). Detailed feature statistics can be found in supplemental experimental procedures 9.

The raw datasets have record-level missing rates of 84.25% for the TJH dataset and 98.38% for the CDSL dataset. High missing rates may impair models' prediction performance.

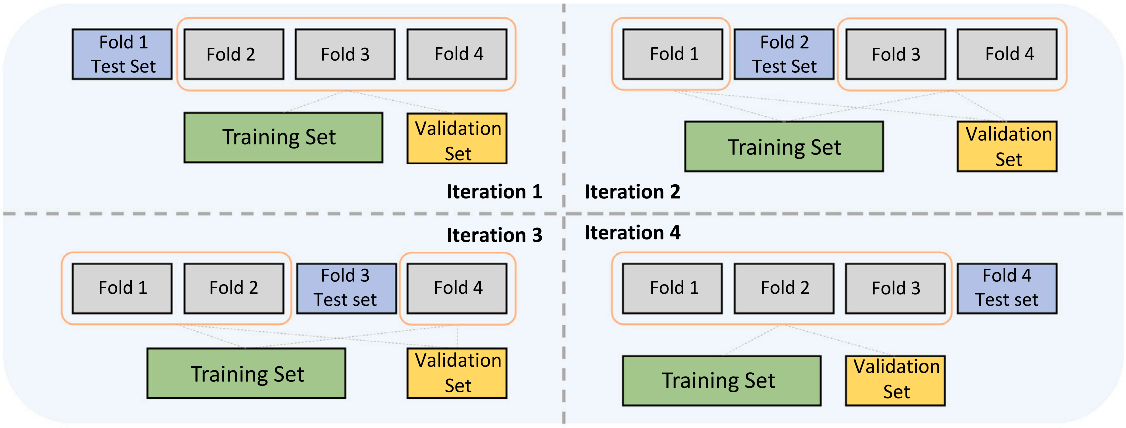

Figure 2. The K-fold cross-validation strategy
We take 4-fold as an example. We use a stratified shuffle split to ensure that the proportions of alive and dead patients on all folds are the same as in the total cohort.

Hence, following the previous benchmark preprocessing settings,[15] we merged data into day level for the TJH dataset and hour level for the CDSL dataset, which reduces the missing rate of recorded physiological characteristics. It is worth noting that for the day/hour that does not have any record, we do not duplicate records from previous time steps. This data merging will not result in significant information loss since most records (over 95%) do not record different values for the same feature on the same day or at the same hour. If there were multiple records for the same feature in the same time slot, we recorded their mean value. For the CDSL dataset, the feature dimensions are much larger and features are sparser than in the TJH dataset: nearly 85% of the features have a missing rate beyond 90%, signifying that over 90% of patients never have records for these features. Therefore, we removed features with a missing rate higher than 90% among all patients.

Table 3. Model parameters and training time

Dataset	TJH		CDSL	
Model	Number of parameters	Runtime (1 epoch)	Number of parameters	Runtime (1 epoch)
RF	–	1.71 s	–	26.69 s
DT	–	1.30 s	–	8.10 s
GBDT	–	4.04 s	–	128.55 s
CatBoost	–	1.54 s	–	7.40 s
XGBoost	–	1.50 s	–	9.40 s
MLP	38.0k	1.91 s	39.5k	6.27 s
RNN	13.9k	1.91 s	17.0k	6.10 s
LSTM	41.0k	1.89 s	48.6k	6.30 s
GRU	31.9k	1.85 s	38.1k	6.84 s
TCN	55.8k	3.31 s	60.4k	31.60 s
Transformer	73.0k	2.29 s	124k	17.37 s
RETAIN	79.0k	2.38 s	135k	60.30 s
StageNet	603k	2.31 s	622k	17.81 s
Dr. Agent	41.9k	2.89 s	48.1k	15.81 s
AdaCare	114k	3.05 s	130k	21.38 s
GRASP	35.3k	2.41 s	39.9k	37.65 s
ConCare	66.4k	2.59 s	89.1k	11.98 s

Data normalization and imputation

We applied Z score normalization to all demographic features, vital signs, lab test features, and LOSs. Here, we calculated the mean and standard deviation based on the data in the 5%–95% quantile range. Preventing future information leakage is crucial for maintaining the fairness of a benchmark. To address this, we implemented stringent strategies: (1) for missing values, we employed a forward-filling imputation method. Specifically, we replaced missing values with the most recent ones. If a patient lacked a prior record for a specific feature, we filled the missing data with the median value from all patients in the training set. (2) We utilized the means and standard deviations from the training set to normalize the entire dataset. This practice ensures no leakage regarding test set data distribution, a detail often overlooked in many previous benchmarking studies.

The data statistics of the two processed datasets are shown in Tables 1 and 2.

Benchmarking experiment settings

To provide a comprehensive benchmark comparison between existing models, we categorized the baseline models into four categories: clinical scoring models, machine-learning models, basic deep-learning models, and EHR-specific predictive models. Machine-learning and basic deep-learning models are popular for general classification and regression tasks. EHR-specific predictive models are designed explicitly for clinical predictive tasks with EHR data. We provide a list of baseline models below, and detailed descriptions of these models can be found in supplemental experimental procedures 1.

(1) Clinical scoring model: 4C mortality score.[29]
(2) Machine-learning models: decision tree (DT), random forest (RF), gradient boosting decision tree (GBDT), XGBoost,[30] CatBoost.[31]
(3) Basic deep-learning models: multilayer perceptron (MLP), RNN,[32] LSTM,[33] gated recurrent units (GRU),[34] temporal convolutional network (TCN),[35] Transformer.[36]

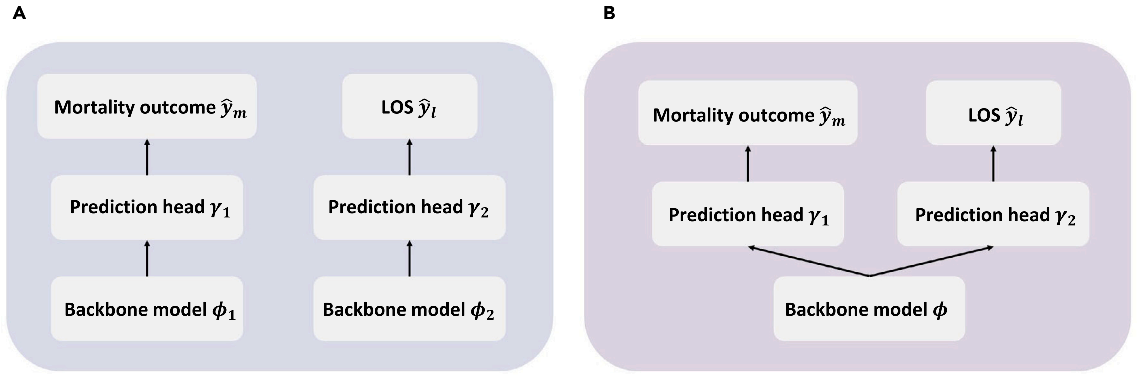

Figure 3. Illustrations of two training settings
(A) The two-stage training setting.
(B) The multitask training setting.

(4) EHR-specific predictive models: RETAIN,[37] StageNet,[38] Dr. Agent,[22] AdaCare,[27] ConCare,[28] GRASP.[39]

Machine-learning models and some deep-learning models (e.g., MLP) cannot handle sequential data as input. When training these models, we use the feature values at the current time step as input and predict the target. The clinical scoring method is applicable only to the mortality-prediction task. To minimize the randomness of the test-sample selection process, we employed the stratified 10-fold cross-validation strategy to train and evaluate the models. We randomly divided patients into 10 groups or "folds." Then, we repeated the training and evaluation process 10 times. In the i-th iteration, we selected the i-th fold as the test set and divided the remaining folds into training and validation sets at an 8:1 ratio. An illustration of this process is provided in Figure 2. Finally, we report the means and standard deviations of the model performances on all test sets from the 10 iterations. Moreover, to evaluate the model's generalizability over time more comprehensively, we have also conducted a standard holdout experiment. We divided the dataset into training, validation, and test sets in a 7:1:2 ratio, based on admission time (using the latest 20% of patients as the holdout dataset). We retrained all models five times with different random seeds and report the standard deviations. The results for the holdout performance are shown in supplemental experimental procedures 4.

Experiments are conducted on a server equipped with dual Intel Xeon Silver 4210R CPUs, each with 10 cores supporting 20 threads, two NVIDIA RTX 3090 GPUs, and 64 GB of RAM. Notably, all experiments are executed solely on one of the GPUs for consistent results. The model parameters and average training time are shown in Table 3.

Hyperparameters for the models are determined using a grid-search strategy on the validation set for each task. Hyperparameter settings can be found in supplemental experimental procedures 2, and the grid search results are available on our online platform. All deep-learning models are trained on the training set for 100 epochs, using an early stopping strategy. During each epoch, we assess the model's performance on the validation set of each fold. After training, we load the model parameters that yield the best performance on the validation set and then evaluate the model on the test set. For the outcome prediction task, we select models based on the AUPRC score. For the LOS-prediction task, we use the MAE score as the criterion. All metrics are computed on a per-record basis.

Model adjustments for the proposed tasks

This research additionally introduces model modifications tailored to the intended tasks in terms of both the loss function and the model architecture.

Outcome-specific length-of-stay prediction

The task of outcome-specific LOS prediction is framed as a multitarget prediction task, for which we employ two distinct strategies. The first is end-to-end multitask learning, whereby a singular model backbone and two MLP prediction heads are trained, one for outcome prediction and the other for LOS prediction. The backbone and prediction heads are co-trained in an end-to-end fashion. The second approach is the two-stage training, in which two separate models, each with identical structures, are trained to predict the outcome and LOS independently. Depictions of these two training scenarios can be found in Figure 3. We include performance metrics for both approaches. It is important to note that traditional machine-learning models accommodate only two-stage settings; hence, we present only the performance metrics of the two-stage training approach for such models.

Early-mortality prediction

Given the nature of the early-mortality prediction task, we devised a straightforward yet potent loss function to guide deep-learning models about the decision time threshold. The suggested TA loss is formulated as follows:

$$\mathcal{L}_{TA} = \frac{1}{T}\sum_{t}^{T}(\mathcal{L}_{CE} * \exp(-\zeta l_t)) - \eta r$$

$$r = \frac{1}{T}\sum_{t}^{T}(l_t * |\hat{y}_t - y_t|).$$

In this formula, $\mathcal{L}_{CE}$ is the cross-entropy loss, l_t is the time to outcome at the t-th time step, ζ denotes the decay rate, η denotes the reward factor, and r denotes the reward term. The second part confers rewards on earlier correct predictions and

Table 4. Benchmarking performance of outcome-specific length-of-stay prediction

Dataset	TJH			CDSL		
Metric	MAE (↓)	MSE (↓)	OSMAE (↓)	MAE (↓)	MSE (↓)	OSMAE (↓)
RF$_t$	4.83 ± 0.53	40.94 ± 8.77	6.14 ± 0.99	4.05 ± 0.13	31.30 ± 4.42	4.90 ± 0.16
DT$_t$	5.07 ± 0.53	50.16 ± 12.06	7.02 ± 1.33	4.11 ± 0.15	32.23 ± 4.81	4.99 ± 0.14
GBDT$_t$	4.79 ± 0.53	42.03 ± 9.58	5.92 ± 0.82	4.01 ± 0.14	30.74 ± 4.55	4.82 ± 0.19
CatBoost$_t$	4.71 ± 0.53	39.72 ± 9.23	5.70 ± 0.85	4.01 ± 0.14	30.62 ± 4.61	4.79 ± 0.17
XGBoost$_t$	4.78 ± 0.55	41.76 ± 9.47	5.77 ± 0.92	4.02 ± 0.13	31.10 ± 4.42	4.81 ± 0.15
MLP$_t$	5.08 ± 0.48	47.21 ± 11.10	6.34 ± 0.97	4.05 ± 0.15	32.08 ± 4.96	4.80 ± 0.17
MLP$_m$	4.90 ± 0.52*#	44.35 ± 14.00*#	7.26 ± 2.81	4.08 ± 0.16	31.54 ± 4.53*#	4.86 ± 0.21
RNN$_t$	4.57 ± 0.48	39.82 ± 10.02	5.54 ± 1.30	3.90 ± 0.22	30.66 ± 5.58	4.37 ± 0.26
RNN$_m$	4.68 ± 0.49	40.21 ± 9.29	5.46 ± 1.43*#	3.87 ± 0.14*#	29.52 ± 4.98*#	4.43 ± 0.18
LSTM$_t$	4.49 ± 0.63	37.38 ± 12.64	4.88 ± 1.30	3.81 ± 0.16	29.29 ± 4.88	4.34 ± 0.23
LSTM$_m$	4.46 ± 0.60*#	39.24 ± 10.30	5.53 ± 1.86	3.85 ± 0.13	30.13 ± 4.22	4.40 ± 0.19
GRU$_t$	4.33 ± 0.49†	35.10 ± 9.61†	5.16 ± 1.31	3.75 ± 0.18	28.90 ± 4.68	4.34 ± 0.32
GRU$_m$	4.80 ± 0.48	41.78 ± 9.49	5.38 ± 1.09	3.98 ± 0.18	32.14 ± 4.31	4.55 ± 0.22
TCN$_t$	4.69 ± 0.60	43.29 ± 13.27	5.77 ± 0.92	3.75 ± 0.17	29.48 ± 4.78	4.31 ± 0.15
TCN$_m$	4.79 ± 0.55	41.56 ± 9.73*#	5.52 ± 0.85*#	3.80 ± 0.16	29.45 ± 4.47*#	4.22 ± 0.18*#†
Transformer$_t$	5.04 ± 0.43	43.88 ± 7.22	5.96 ± 1.62	3.98 ± 0.20	32.35 ± 5.61	5.19 ± 0.19
Transformer$_m$	5.06 ± 0.46	46.02 ± 11.63	6.61 ± 1.57	4.00 ± 0.20	32.02 ± 4.68*#	5.13 ± 0.19*#
RETAIN$_t$	4.64 ± 0.61	41.22 ± 14.45	4.77 ± 1.18	3.83 ± 0.16	30.56 ± 5.99	4.41 ± 0.26
RETAIN$_m$	4.62 ± 0.55*#	40.06 ± 10.34*#	5.03 ± 0.96	3.84 ± 0.20	29.92 ± 4.48*#	4.41 ± 0.30
StageNet$_t$	4.60 ± 0.76	41.70 ± 14.21	5.59 ± 1.33	3.72 ± 0.15†	28.81 ± 4.57†	4.33 ± 0.22
StageNet$_m$	4.49 ± 0.42*#	39.55 ± 8.52*#	7.10 ± 1.74	3.78 ± 0.18	29.93 ± 4.50	4.28 ± 0.19*#
Dr. Agent$_t$	4.61 ± 0.58	40.85 ± 12.12	4.93 ± 1.14	3.80 ± 0.18	29.70 ± 4.96	4.46 ± 0.27
Dr. Agent$_m$	4.41 ± 0.58*#	37.55 ± 11.38*#	4.75 ± 1.11*#†	3.82 ± 0.19	29.45 ± 4.70*#	4.49 ± 0.28
AdaCare$_t$	4.55 ± 0.61	39.67 ± 11.35	5.08 ± 0.62	3.80 ± 0.15	29.07 ± 4.57	4.42 ± 0.15
AdaCare$_m$	4.41 ± 0.63*#	38.86 ± 10.75*#	5.24 ± 0.98	3.81 ± 0.16	32.05 ± 4.27	4.43 ± 0.18
GRASP$_t$	4.57 ± 0.43	40.65 ± 11.46	5.51 ± 1.13	3.84 ± 0.16	29.95 ± 5.06	4.43 ± 0.25
GRASP$_m$	4.44 ± 0.50*#	37.79 ± 10.27*#	5.31 ± 1.31*#	3.89 ± 0.12	30.01 ± 4.33	4.49 ± 0.28
ConCare$_t$	4.69 ± 0.52	40.26 ± 11.13	5.29 ± 1.31	3.81 ± 0.17	30.16 ± 5.03	4.29 ± 0.23
ConCare$_m$	4.67 ± 0.56*#	40.42 ± 10.77	5.27 ± 1.35*#	3.85 ± 0.11	29.15 ± 3.90*#	4.46 ± 0.24

The reported score is in the form of mean ± SD. Subscript m signifies a multitask learning strategy, while subscript t indicates a two-stage learning strategy. †The best performance. #The multitask setting outperforms the two-stage learning strategy. *The performance improvement against the two-stage model is statistically significant ($p < 0.05$).

penalizes late incorrect predictions by either decreasing or increasing the loss values. The first part of the proposed loss function works to prevent early incorrect predictions from incurring excessive penalties through the application of an exponential decay function. This proposed TA loss function can be readily integrated into any deep-learning model, thereby improving early-mortality prediction performance. It should be noted that as the loss term is employed only during the training phase, the l_t term is not required for the inference stage. Consequently, this ensures that there is no future information leakage at the inference stage.

In this section, we present comprehensive benchmarking results of all predictive models across all datasets and tasks. To assess the statistical significance of our model's performance, we performed t tests on all results. The bootstrap T test[40] was employed to calculate p values. We set the sample number for the bootstrap at 1,000 and the number of sampling processes at 50. The comparisons were made between different variations of the model, for instance, comparing the model with and without TA loss in the early-mortality prediction task and between two-stage and multitask settings in the LOS-prediction task. The p values are reported in supplemental experimental procedures 7.

Benchmarking performance of outcome-specific length-of-stay prediction

The benchmarking performances for the task of outcome-specific LOS prediction, under both multitask and two-stage settings, are presented in Table 4. On the TJH dataset, the GRU model with two-stage learning achieves the lowest MAE and MSE. Dr. Agent with multitask learning demonstrates the lowest OSMAE. On the CDSL dataset, StageNet with two-stage learning achieves the lowest MAE and MSE, while the TCN model with multitask learning achieves the lowest OSMAE.

We observe that the multitask strategy generally outperforms the two-stage strategy on the TJH dataset. On the

Table 5. Benchmarking performance on the task of early-mortality prediction

Dataset	TJH			CDSL		
Metric	AUPRC(↑)	AUROC(↑)	ES(↑)	AUPRC(↑)	AUROC(↑)	ES(↑)
4C	89.84 ± 4.46	94.16 ± 2.57	–	23.93 ± 2.99	76.15 ± 4.06	–
RF	95.56 ± 2.69	96.58 ± 2.20	72.63 ± 9.15	49.48 ± 3.79	84.35 ± 2.66	−10.54 ± 3.57
DT	80.48 ± 7.26	87.41 ± 3.99	67.86 ± 11.24	38.27 ± 5.21	79.67 ± 4.61	−8.23 ± 4.17
GBDT	95.13 ± 3.51	96.41 ± 2.35	76.45 ± 7.13	50.32 ± 5.15	85.15 ± 2.83	2.25 ± 4.86
CatBoost	95.99 ± 2.61	97.14 ± 1.81	74.91 ± 9.00	50.86 ± 4.34	85.09 ± 2.86	−3.94 ± 3.89
XGBoost	95.70 ± 2.98	96.84 ± 2.09	76.41 ± 9.90	49.70 ± 5.06	84.59 ± 3.09	−3.12 ± 4.44
MLP	93.78 ± 2.80	95.95 ± 1.62	72.87 ± 8.22	48.60 ± 3.57	84.15 ± 2.72	−0.76 ± 3.95
MLP-TA	93.21 ± 2.78	95.65 ± 1.85	73.94 ± 8.96[#]	48.67 ± 3.07[#]	84.24 ± 2.56[#]	0.55 ± 3.71[#]
RNN	96.03 ± 3.42	97.41 ± 2.19	78.33 ± 11.35	57.57 ± 6.55	87.81 ± 2.55	19.66 ± 8.86
RNN-TA	95.97 ± 3.65	97.38 ± 2.29	79.79 ± 11.06[*#]	58.21 ± 6.34[*#]	88.01 ± 2.36[*#]	20.15 ± 11.25[*#]
LSTM	94.82 ± 7.65	97.08 ± 3.65	84.80 ± 10.68	55.53 ± 8.54	87.02 ± 3.01	19.63 ± 11.41
LSTM-TA	95.40 ± 5.53[*#]	97.10 ± 3.05[*#]	83.78 ± 8.30	56.89 ± 7.34[#]	87.76 ± 2.50[#]	20.92 ± 13.10[#]
GRU	96.33 ± 3.18	97.59 ± 2.13	78.33 ± 15.45	56.78 ± 8.02	87.93 ± 2.89	21.66 ± 8.86
GRU-TA	96.50 ± 3.04[#]	97.70 ± 2.06[#]	80.93 ± 13.84[*#]	57.75 ± 5.36[*#]	87.98 ± 2.62[*#]	23.54 ± 8.10[*#]
TCN	93.13 ± 5.10	96.39 ± 1.89	74.66 ± 13.03	57.09 ± 5.84	87.40 ± 2.67	13.48 ± 12.04
TCN-TA	93.31 ± 4.97[#]	96.74 ± 1.87[*#]	76.47 ± 12.27[#]	57.71 ± 5.72[#]	87.59 ± 2.60[#]	21.59 ± 12.68[*#]
Transformer	93.47 ± 6.73	96.86 ± 2.13	79.21 ± 13.05	38.34 ± 5.07	81.32 ± 3.46	−3.96 ± 13.23
Transformer-TA	93.86 ± 6.42[#]	97.01 ± 2.01[#]	80.73 ± 12.47[*#]	40.18 ± 5.00[#]	82.36 ± 3.61[*#]	−0.78 ± 11.42[*#]
RETAIN	96.49 ± 2.05	97.84 ± 1.44	82.59 ± 12.29	54.54 ± 7.97	85.02 ± 3.93	7.32 ± 10.67
RETAIN-TA	95.99 ± 2.42	97.82 ± 1.51	82.85 ± 11.36[*#]	54.30 ± 6.50	85.43 ± 2.81[#]	9.00 ± 7.66[#]
StageNet	95.71 ± 3.77	97.27 ± 2.38	77.44 ± 12.52	56.57 ± 6.82	87.09 ± 3.54	23.93 ± 9.26
StageNet-TA	95.79 ± 3.86[*#]	97.32 ± 2.41[*#]	78.88 ± 12.29[*#]	58.19 ± 6.48[*#]	88.18 ± 2.82[*#†]	24.55 ± 9.32[#†]
Dr. Agent	97.22 ± 2.44	98.00 ± 1.75	85.80 ± 7.97	54.17 ± 8.92	86.76 ± 3.64	17.53 ± 11.25
Dr. Agent-TA	97.16 ± 2.56	98.00 ± 1.97	86.01 ± 7.66[#†]	53.06 ± 7.34	87.06 ± 3.30[*#]	19.35 ± 9.60[*#]
AdaCare	97.86 ± 1.09	98.53 ± 0.77	78.39 ± 7.51	55.32 ± 4.45	86.59 ± 1.99	17.46 ± 8.66
AdaCare-TA	98.11 ± 1.13[#†]	98.64 ± 0.89[#†]	84.51 ± 8.17[*#]	55.62 ± 5.44[#]	87.00 ± 2.18[#]	20.09 ± 9.86[*#]
GRASP	96.04 ± 3.15	97.30 ± 2.30	81.63 ± 9.71	53.95 ± 8.37	84.44 ± 4.40	15.27 ± 15.17
GRASP-TA	96.16 ± 3.17[#]	97.40 ± 2.21[#]	82.82 ± 9.08[#]	53.50 ± 7.89	84.91 ± 3.73[*#]	15.61 ± 13.76[*#]
ConCare	97.01 ± 2.46	97.89 ± 1.62	80.97 ± 13.66	58.59 ± 6.49	87.76 ± 3.02	17.58 ± 12.34
ConCare-TA	97.04 ± 2.51[#]	97.94 ± 1.62[#]	82.31 ± 13.08[*#]	58.68 ± 7.04[#†]	87.96 ± 3.23[#]	20.90 ± 10.43[#]

The reported score is of the form mean ± SD. "TA" denotes that the model trained with the time-aware loss. [†]The best performance. [#]The model with time-aware loss outperforms the original model. *The performance improvement against the model without the TA version is statistically significant ($p < 0.05$). All three metrics are multiplied by 100 for readability purposes.

TJH dataset, we find that EHR-specific models can better benefit from multitask learning settings, suggesting that the multitask setting may better facilitate these models' ability to map patients' diverse statuses in the latent space. We visualize the learned embeddings in the latent space using t-distributed stochastic neighbor embedding (t-SNE) in supplemental experimental procedures 6. In addition, it is important to note the discrepancy between OSMAE and MAE. Despite both metrics assessing absolute error, the OSMAE values are notably larger than the MAE values on both datasets. On average, the OSMAE is 22% higher than the MAE on the TJH dataset and 17% higher on the CDSL dataset. This suggests that MAE might potentially distort the evaluation of model performance, as it cannot measure the error for false-negative and false-positive predictions, despite its widespread use in previous LOS-prediction studies. We also conduct the error analysis for the MAE in supplemental experimental procedures 8. In our current setting, we utilize two identical model structures for two-stage training. However, future work could potentially involve the use of distinct models for the two tasks. Further research could also focus on designing a more sophisticated multitask architecture or incorporating the principles of transfer learning.

Benchmarking performance of early mortality prediction

The comparative performances of the models in early-mortality prediction are presented in Table 5. We also introduced the TA loss, labeling these adjusted models with a "TA" suffix for all deep-learning models. For the TJH dataset, the AdaCare-TA model delivered the highest AUPRC and AUROC, while Dr. Agent provided the highest ES. In contrast, for the CDSL dataset, ConCare-TA achieved the highest AUPRC, while StageNet-TA produced the highest AUROC and ES. Among

CellPress
OPEN ACCESS

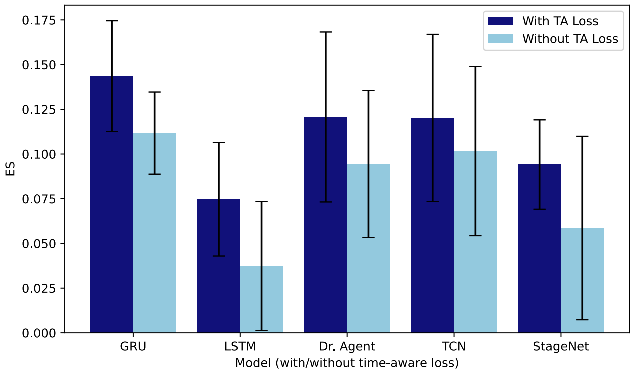

Figure 4. Early-prediction performance of five models with the highest ES on the CDSL dataset

All models were trained using the first half of patient records. Error bars are standard deviations. All performance improvements are statistically significant ($p < 0.05$).

traditional machine-learning and basic deep-learning models, CatBoost and GRU showed better performance on both datasets.

The results indicate that the proposed TA loss term can enhance the performance of most models, particularly in terms of the ES. The average improvement of the ES is 1.93% for the TJH dataset and 33.58% for the CDSL dataset. This underscores that this simplistic loss term can effectively aid models in making correct early decisions. It is worth noting that our goal is to evaluate the direct effectiveness of the proposed TA loss term, so we did not specifically tune the hyperparameters of all TA models. So even under this adverse condition, most TA models can still outperform the original version. In addition, we observe that for the TJH dataset, the performance of all models was higher, especially for the ES, suggesting that the task is more straightforward on this dataset. Patients' initial status was also more severe in the TJH dataset, resulting in a much higher ES than in the CDSL dataset. This might be attributable to the fact that the TJH data were collected during the initial months of the pandemic, when the virus was significantly more lethal. The performance of EHR-specific models generally surpasses that of basic deep-learning models, which in turn outperform traditional machine-learning models. This is to be expected, as most EHR-specific models are better equipped to utilize characteristics present in EHR data, such as disease progression, while compared with deep-learning models, traditional machine-learning models are unable to leverage temporal relationships in sequential data.

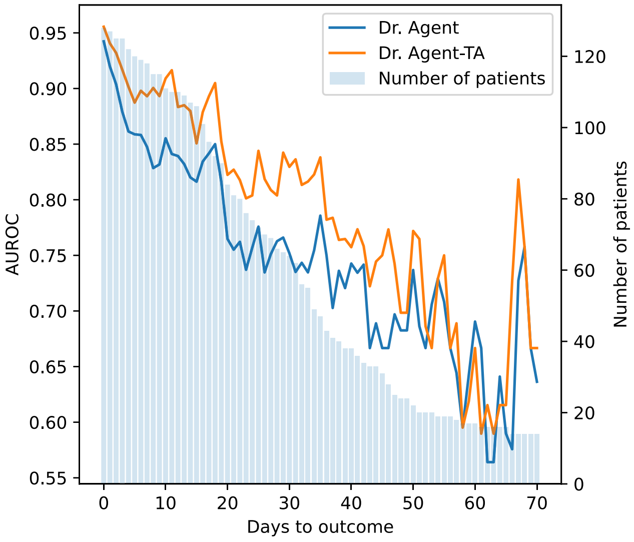

Figure 5. AUROC of Dr. Agent and Dr. Agent-TA at each visit

We also plot the distribution of the number of patients on different days to outcome groups.

DISCUSSION

In this section, we further explore the models' performance in terms of the proposed metrics ES and OSMAE. These metrics show the models' performance on the early and outcome-specific perspectives of two tasks. We also discuss the limitations and future directions of this work.

Analysis of early-prediction performance

By enhancing early-prediction performance, models can discern patients' health risks in the early stages. This can address the "cold-start" issue of predictive models, which is particularly beneficial during the early phases of a pandemic, when predictions must rely on sparse data. To assess the efficacy of our proposed early-prediction loss term in improving early-stage predictions, we simulated a scenario where the model generates forecasts using only the first half of a patient's visit records. The ESs for the top five best-performing models (both with and without the TA loss) on the CDSL dataset are illustrated in Figure 4. The findings demonstrate that models incorporating the TA loss consistently achieve superior ESs in the early stages, which directly assesses the true-positive rate of predictions. This underscores the adaptability of our proposed TA loss in enhancing various models' early-prediction capabilities.

To offer a more granular view of the performance gains attributed to the TA loss, we have charted the AUROC at different visits (days to outcome) for the Dr. Agent model on the CDSL dataset as a case study in Figure 5. The figure shows that most patients' LOS is less than 30 days, and the AUROC of the model with the TA loss is significantly higher. This suggests that, for most patients, the TA loss is more effective in identifying early health risks.

Case study of prediction metrics

The proposed ES and OSMAE metrics are inherently interpretable, as they are based solely on straightforward score calculations, independent of the models. This makes the calculation process transparent and easy to understand. To further elucidate our metrics, we have included a case study here, illustrated in Figure 6.

In Figure 6A, we plot the predicted risk probability over time alongside the ES at each time step for a patient whose actual outcome was mortality. Initially, the model accurately identifies early risk by predicting a risk probability above 0.5, earning a full ES (1.0) for these early correct predictions. However, over

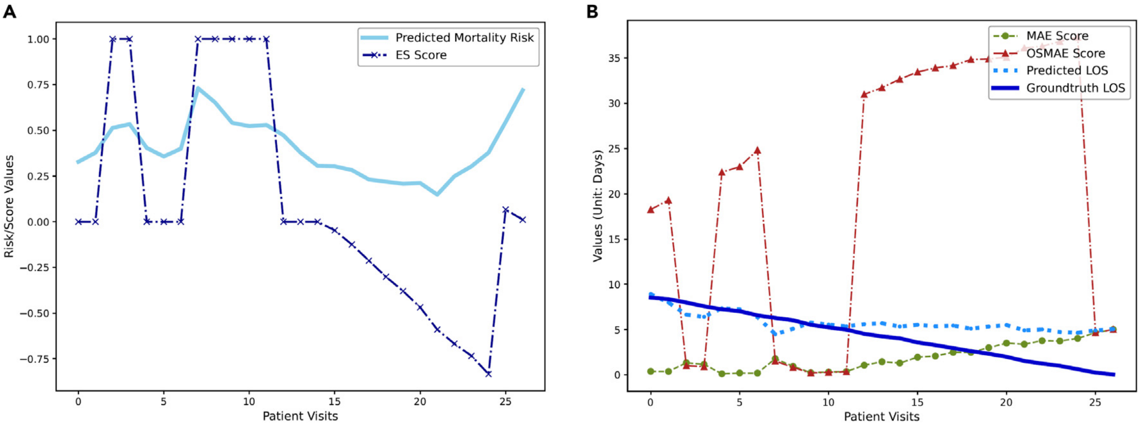

Figure 6. Case prediction plot on the CDSL dataset

(A) ES and risk curve.

(B) OSMAE, MAE, predicted LOS, and ground-truth LOS curve. We use the TCN model with the multitask setting to generate the prediction results.

time, as the model incorrectly assesses the patient's condition as improving, it receives progressively lower ESs. This decline reflects the model's erroneous predictions as the patient approaches the final outcome. Near the end, even though the model predicts high risks again, the rewards for these correct predictions are lower due to their proximity to the final outcome.

In Figure 6B, we show the predicted LOS, the actual LOS, the MAE, and the OSMAE for the same patient. At first glance, the predicted LOS (dotted blue line) closely matches the actual LOS (solid blue line), resulting in a consistently low MAE (dotted green line). However, the higher OSMAE (red line) indicates that the model is making incorrect outcome predictions, suggesting a recovery when the patient's condition is actually deteriorating. When outcomes are predicted accurately, OSMAE aligns with MAE. Using traditional MAE as a sole metric could lead to biased decisions, as it may not reflect the patient's actual health trajectory.

This case study demonstrates that our proposed ES and OSMAE metrics can more accurately represent a model's ability to assess early patient risk and understand disease progression, aspects often overlooked in traditional classification and regression metrics.

Outcome-specific prediction performance

Another primary objective of our study is to determine how the proposed outcome-specific LOS-prediction task can enhance clinical decision-making. In Table 6, we display the prediction discrepancies for the top five models with the highest ΔMAE (OSMAE − MAE). To demonstrate how ΔMAE can retain information about the correctness of outcome predictions while computing the MAE, we also include the number and percentage of patients with incorrect outcome predictions.

Table 6. Prediction discrepancy of five models on the CDSL dataset

Model	Type	ΔMAE	Outcome wrong (n)	Outcome wrong (%)
Transformer	Two-stage	1.21	1,469	11.89%
Transformer	Multitask	1.13	1,389	11.24%
MLP	Multitask	0.78	1,322	10.70%
MLP	Two-stage	0.75	1,295	10.48%
Dr. Agent	Multitask	0.67	1,108	8.97%

The results indicate that as ΔMAE increases, the percentage of incorrect outcome predictions also rises. This suggests that an increase in outcome prediction error contributes to the discrepancy between our new OSMAE metric and the traditional MAE. This conclusion further implies that in traditional LOS-prediction tasks, relying solely on MAE to assess prediction accuracy can lead to erroneous conclusions and potentially compromise clinical decision-making. Consequently, refining the OSMAE metric—rather than solely concentrating on the conventional MAE—is crucial for future predictive modeling endeavors, possibly through a more judicious multitask learning framework or regularizer.

Sensitivity analysis of OSMAE and ES

We investigate the influence of the threshold parameter γ on the OSMAE and the ES. The OSMAE and ES values for various γ thresholds, using the same GRU multitask model on the CDSL dataset, are plotted in Figure 7. Evidently, as γ increases, OSMAE increases and ES decreases. This occurs because a larger γ requires the model to accurately predict the target earlier, thereby increasing the task's difficulty. In practical clinical settings, clinicians can choose an appropriate threshold based on their specific requirements. Another advantage of this γ setting is making both metrics invariant to the record length, which means patients with too many visits bring less bias to the proposed metrics compared with traditional AUROC and AUPRC. This is because both metrics are normalized by the visit time, ensuring that the length of a patient's record does not disproportionately influence the metric values, regardless of the duration of the patient record. No matter how long the patient record is, the visits before the penalty time γ are regularized to have less effect on the metric values.

We also aim to explore the effect of penalty score value in ES for false-negative and false-positive cases. We selected −0.1 as

CellPress
OPEN ACCESS

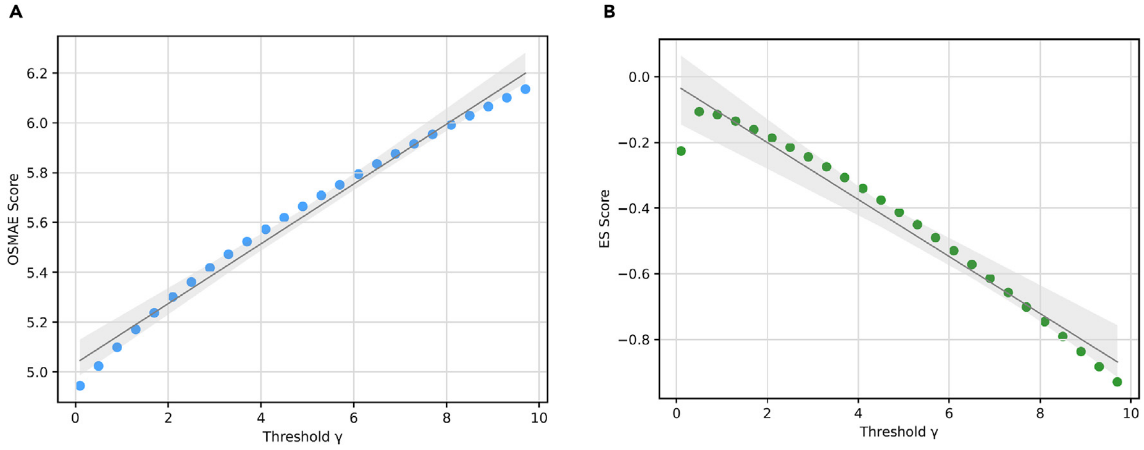

Figure 7. OSMAE and ES values with different γ

(A) OSMAE with γ from 0 to 10.

(B) ES with γ from 0 to 10. The line indicates the fitted regression line and the shadow indicates 95% confidence intervals. Scores were calculated using the same TCN multitask model on the CDSL dataset.

the penalty score for false-positive cases to mirror scenarios in real-world clinical settings, where the costs associated with false-negative predictions are typically higher than those for false positives, particularly in intensive care environments.[41,42] Thus, a lower penalty score is assigned to false-positive predictions. However, clinicians may adjust this penalty score to suit their specific needs in various applications. For example, in a less critical clinical setting, clinicians may set higher penalty scores to select the model that produces fewer false alarms. Since the early-prediction score (ES) functions solely as a metric, it does not influence model performance. Figure 8 illustrates how the penalty score affects the ES value. As demonstrated in the figure, while the penalty score influences the absolute value of ES, it does not alter the underlying trend. True-negative cases receive a score of zero, aligning with the ES metric's aim to evaluate models for early risk detection in patients. Since true-negative predictions do not trigger alarms, the model is neither rewarded nor penalized for these outcomes. It is important to note that the number of true-positive predictions is 649, compared with 10,381 true-negative predictions. Awarding points for true-negative predictions would introduce significant bias into the final score.

Model interpretability, limitations, and future works

Interpretability is an important aspect of models, especially in clinical settings. With model interpretability, the clinicians can understand the model's prediction process and make reliable clinical decisions. Many models benchmarked in this work claim they have different levels of interpretability. We group them into three groups: (1) visit level, (2) feature level, (3) others. Visit-level interpretability indicates that the model can identify the most important visit from longitudinal EHR sequences, and

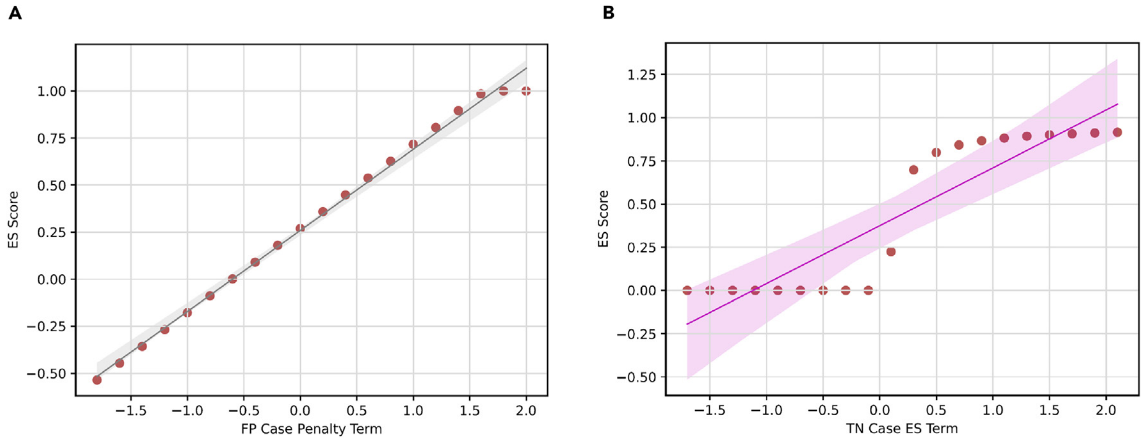

Figure 8. ES values with different penalty terms for a true-negative (TN) case and a false-positive (FP) case ranging from –2 to 2

(A) FP case penalty term.

(B) TN case penalty term. The line indicates the fitted regression line and the shadow indicates 95% confidence intervals. Scores are calculated using the same TCN multitask model on the CDSL dataset.

Table 7. Interpretability characteristics of all interpretable models

Model	Visit level	Feature level	Others
4C	–	✓	–
RF	–	✓	–
DT	–	✓	–
GBDT	–	✓	–
CatBoost	–	✓	–
XGBoost	–	✓	–
RETAIN	✓	✓	–
StageNet	–	–	disease progression score
Dr. Agent	✓	–	–
AdaCare	–	✓	–
GRASP	–	–	patient clusters
ConCare	–	✓	feature correlations

feature-level interpretability means that the model can identify the most important risk factors from single-visit data. Some models can provide other information to aid clinical decisions such as disease progression score. We summarize the characteristics of all interpretable models in Table 7.

We find that most machine-learning models are interpretable, as they are tree-based models. These models can provide only feature-level interpretability (i.e., feature-importance score) because they cannot process temporal dependencies. All naive deep-learning models are black-box models, and they are not interpretable. However, all EHR-specific deep-learning models studied in this work are designed to provide some interpretability. RETAIN can provide both visit-level and feature-level interpretabilities. StageNet, GRASP, and ConCare can provide additional information, including disease progression score, patient clustering information, and feature correlations.

We aim to establish a comprehensive benchmark for COVID-19 predictive modeling. However, our study has certain limitations. First, within our two-stage approach, the models predicting mortality and LOS might have distinct architectures. For comparison purposes, due to the inherent complexity, we assessed only scenarios where both models are identical. In addition, the multitask setting may benefit from more intricate multitask learning structures. Exploring various model combinations and learning structures is a promising avenue for future work. Second, during data preprocessing, we imputed missing values using the most recent measurement. While this method has been adopted in numerous studies,[15,27,28] it is essential to investigate how different imputation techniques influence the outcomes in a clinical context. Last, when employing the OSMAE metric, other consequential clinical outcomes, like re-admission, in addition to mortality and recovery, should be considered. Future studies should account for these competing factors by devising more inclusive tasks and metrics. Our proposed tasks align with clinical practice requirements. Beyond task and metric design, the introduced early-prediction loss and multitask learning framework are innovative approaches yielding encouraging results for the tasks presented. We anticipate further refinements in future research endeavors.

We envision that our proposed benchmark analysis pipeline and coding framework can be extended to a wider range of clinical tasks and datasets. In this work, to bolster the comprehensiveness and validity of our evaluation, we incorporated an expansive selection of ICU datasets, namely MIMIC-III[17,43] and MIMIC-IV.[44,45] We undertook the in-hospital-mortality-prediction task using these two datasets, with results detailed in supplemental experimental procedures 5. While our efforts remain exploratory in nature, we aspire for our proposed tasks to be integrated across diverse clinical datasets, thereby enhancing the quality of health-care delivery. Another

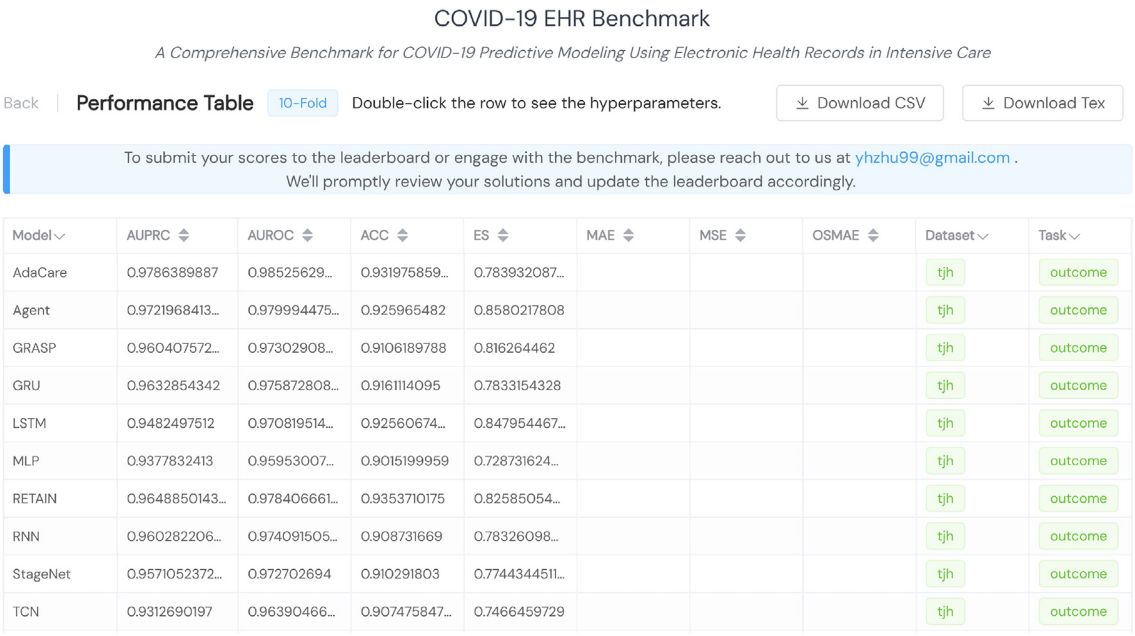

Figure 9. Screenshot of the online benchmark results visualization system

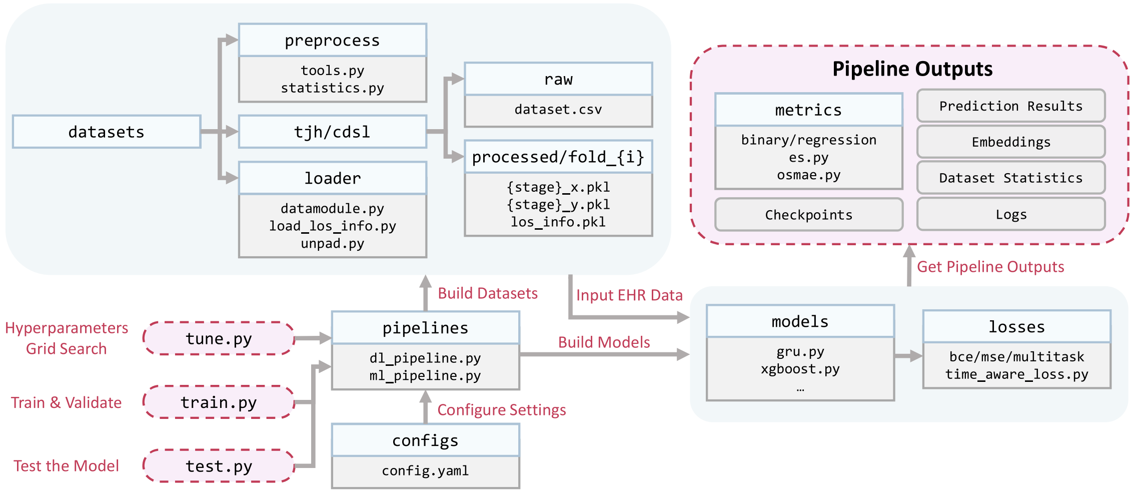

Figure 10. Benchmark code structure

line for future research pertains to benchmarking the fairness of model predictions. We have undertaken preliminary experiments using the GRU model with four fairness metrics, with results detailed in supplemental experimental procedures 3. Subsequent efforts might focus on a thorough analysis aimed at minimizing bias and enhancing fairness across various predictive models.

Conclusion

This paper presents a fair, comprehensive, and open-source benchmark for predictive modeling of COVID-19, using EHR data from ICUs. We introduce two innovative clinical prediction tasks—early-mortality prediction and outcome-specific LOS prediction—based on the clinical practice of COVID-19 prediction scenarios on two real-world datasets. Our approach ensures fairness and reproducibility through the implementation of meticulous data processing and model training pipelines. We provide benchmarking performances for a total of 17 predictive models, including 5 traditional machine-learning models, 6 basic deep-learning models, and 6 state-of-the-art EHR-specific deep-learning models. To improve accessibility, we have made our benchmarking results and trained models readily available online. This not only facilitates quick access to prediction results using our trained models, but also grants clinicians and researchers the ability to obtain swift prediction results using our trained models. Our endeavors aim to spur continuous development and advancement in the field of deep learning and machine learning, particularly in the context of pandemic predictive modeling.

EXPERIMENTAL PROCEDURES

Resource availability

Lead contact

Liantao Ma is the lead contact of this study and can be reached via e-mail (malt@pku.edu.cn).

Materials availability

This study did not generate new unique reagents.

Data and code availability

- This research did not involve the collection of new patient EHR data. The TJH EHR dataset[2] utilized in this study is publicly available on GitHub (https://github.com/HAIRLAB/Pre_Surv_COVID_19). The CDSL dataset[25] is open to global researchers and can be accessed on request (https://hmhospitales.com/prensa/notas-de-prensa/comunicado-covid-data-save-lives). To gain access, applicants should fill out the request form. We use these datasets under their respective licenses.
- Furthermore, we have made our benchmarking system available online at https://pyehr.netlify.app, as illustrated in Figure 9. All model performances with their associated hyperparameter combinations on both tasks are accessible in the system. These results were used to generate all the performance analysis figures presented in this work. The tables are designed for easy querying, comparison, and sorting. Users can select specific rows and download the CSV files or LaTeX codes. In addition, all trained models are also released online, enabling clinicians and researchers to conveniently use these models to obtain prediction results with their own test samples. The accession number for the source code of this online system reported in this paper is Zenodo: https://doi.org/10.5281/zenodo.10573647[24]. Researchers can easily deploy an offline version.
- The accession number for the benchmarking code reported in this paper is Zenodo: https://doi.org/10.5281/zenodo.10573567.[23] The code structure of this benchmarking pipeline is depicted in Figure 10. The diagram is essentially self-explanatory. All runtime configurations for model parameters are stored in configs/directory. All deep-learning and machine-learning models are implemented with various modules. This clearly structured and modular design allows users to effortlessly extend the existing models, design new models and tasks, or even introduce new datasets by adding corresponding components without having an impact on the downstream benchmark prediction and evaluation process. The analytical files generated in this work are also publicly available online. These data analysis files are housed in the datasets/ folder of the code repository.

SUPPLEMENTAL INFORMATION

Supplemental information can be found online at https://doi.org/10.1016/j.patter.2024.100951.

ACKNOWLEDGMENTS

This work was supported by the National Natural Science Foundation of China (82241052). J.G. acknowledges the receipt of studentship awards from the Health Data Research UK-The Alan Turing Institute Wellcome PhD Programme in Health Data Science (grant 218529/Z/19/Z). L.M.

acknowledges the Xuzhou Scientific and Technological Projects (KC23143). We acknowledge Yan et al. and HM Hospitales for their contributions to making the EHR datasets publicly available. Their dedication to open science has been instrumental in advancing our research and the broader field of health-care analytics.

AUTHOR CONTRIBUTIONS

Conceptualization, J.G. and L.M.; methodology, J.G., Y.Z., and W.W.; software, Y.Z., W.W., and Z.W.; validation, J.G., Y.Z., and W.W.; data curation, Y.Z.; writing – original draft, J.G., Y.Z., and W.W.; writing – review & editing, G.D., W.T., H.W., Y.W., E.M.H., and L.M.; visualization, J.G., Y.Z., W.W., and Z.W.; supervision, J.G., E.M.H., and L.M.

DECLARATION OF INTERESTS

The authors declare no competing interests.

Received: September 21, 2023
Revised: February 12, 2024
Accepted: February 13, 2024
Published: March 7, 2024

REFERENCES

1. Callaway, E., and Ledford, H. (2021). How bad is omicron? what scientists know so far. Nature *600*, 197–199.
2. Yan, L., Zhang, H.-T., Goncalves, J., Xiao, Y., Wang, M., Guo, Y., Sun, C., Tang, X., Jing, L., Zhang, M., et al. (2020). An interpretable mortality prediction model for covid-19 patients. Nat. Mach. Intell. *2*, 283–288.
3. Jamshidi, E., Asgary, A., Tavakoli, N., Zali, A., Setareh, S., Esmaily, H., Jamaldini, S.H., Daaee, A., Babajani, A., Sendani Kashi, M.A., et al. (2021). Using machine learning to predict mortality for covid-19 patients on day 0 in the icu. Front. Digit. Health *3*, 681608. https://doi.org/10.3389/fdgth.2021.681608.
4. Martin, B., DeWitt, P.E., Russell, S., Anand, A., Bradwell, K.R., Bremer, C., Gabriel, D., Girvin, A.T., Hajagos, J.G., McMurry, J.A., et al. (2022). Characteristics, outcomes, and severity risk factors associated with sars-cov-2 infection among children in the us national covid cohort collaborative. JAMA Netw. Open *5*, e2143151.
5. Nachega, J.B., Sam-Agudu, N.A., Machekano, R.N., Rabie, H., van der Zalm, M.M., Redfern, A., Dramowski, A., O'Connell, N., Pipo, M.T., Tshilanda, M.B., et al. (2022). Assessment of clinical outcomes among children and adolescents hospitalized with covid-19 in 6 sub-saharan african countries. JAMA Pediatr. *176*, e216436.
6. Domínguez-Rodríguez, S., Villaverde, S., Sanz-Santaeufemia, F.J., Grasa, C., Soriano-Arandes, A., Saavedra-Lozano, J., Fumadó, V., Epalza, C., Serna-Pascual, M., Alonso-Cadenas, J.A., et al. (2021). A bayesian model to predict covid-19 severity in children. Pediatr. Infect. Dis. J. *40*, e287–e293.
7. Oliveira, E.A., Simões E Silva, A.C., Oliveira, M.C.L., Colosimo, E.A., Mak, R.H., Vasconcelos, M.A., Miranda, D.M., Martelli, D.B., Silva, L.R., Pinhati, C.C., and Martelli-Júnior, H. (2022). Comparison of the first and second waves of the coronavirus disease 2019 pandemic in children and adolescents in a middle-income country: Clinical impact associated with severe acute respiratory syndrome coronavirus 2 gamma lineage. J. Pediatr. *244*, 178–185.e3.
8. Bennett, T.D., Moffitt, R.A., Hajagos, J.G., Amor, B., Anand, A., Bissell, M.M., Bradwell, K.R., Bremer, C., Byrd, J.B., Denham, A., et al. (2021). Clinical characterization and prediction of clinical severity of sars-cov-2 infection among us adults using data from the us national covid cohort collaborative. JAMA Netw. Open *4*, e2116901.
9. Elliott, J., Bodinier, B., Whitaker, M., Delpierre, C., Vermeulen, R., Tzoulaki, I., Elliott, P., and Chadeau-Hyam, M. (2021). Covid-19 mortality in the uk biobank cohort: revisiting and evaluating risk factors. Eur. J. Epidemiol. *36*, 299–309.
10. Gao, J., Yang, C., Heintz, J., Barrows, S., Albers, E., Stapel, M., Warfield, S., Cross, A., and Sun, J.; N3C consortium (2022). Medml: Fusing medical knowledge and machine learning models for early pediatric covid-19 hospitalization and severity prediction. iScience *25*, 104970. https://doi.org/10.1016/j.isci.2022.104970.
11. Wynants, L., Van Calster, B., Collins, G.S., Riley, R.D., Heinze, G., Schuit, E., Bonten, M.M.J., Dahly, D.L., Damen, J.A.A., Debray, T.P.A., et al. (2020). Prediction models for diagnosis and prognosis of covid-19: systematic review and critical appraisal. BMJ *369*, m1328. https://doi.org/10.1136/bmj.m1328.
12. Shakeel, S.M., Kumar, N.S., Madalli, P.P., Srinivasaiah, R., and Swamy, D.R. (2021). Covid-19 prediction models: A systematic literature review. Osong Public Health Res. Perspect. *12*, 215–229.
13. Pirracchio, R. (2016). Mortality prediction in the icu based on mimic-ii results from the super icu learner algorithm (sicula) project. Secondary Analysis of Electronic Health Records *295*.
14. Purushotham, S., Meng, C., Che, Z., and Liu, Y. (2018). Benchmarking deep learning models on large healthcare datasets. J. Biomed. Inf. *83*, 112–134.
15. Harutyunyan, H., Khachatrian, H., Kale, D.C., Ver Steeg, G., and Galstyan, A. (2019). Multitask learning and benchmarking with clinical time series data. Sci. Data *6*. 96–18.
16. Yèche, H., Kuznetsova, R., Zimmermann, M., Hüser, M., Lyu, X., Faltys, M., and Rätsch, G. (2021). Hirid-icu-benchmark–a comprehensive machine learning benchmark on high-resolution icu data. Preprint at arXiv. https://doi.org/10.48550/arXiv.2111.08536.
17. Johnson, A.E., Pollard, T.J., Shen, L., Lehman, L.-w. H., Feng, M., Ghassemi, M., Moody, B., Szolovits, P., Anthony Celi, L., and Mark, R.G. (2016). Mimic-iii, a freely accessible critical care database. Sci. Data *3*, 160035–160039.
18. Lee, J., Scott, D.J., Villarroel, M., Clifford, G.D., Saeed, M., and Mark, R.G. (2011). Open-access mimic-ii database for intensive care research. In 2011 Annual International Conference of the IEEE Engineering in Medicine and Biology Society (IEEE), pp. 8315–8318.
19. Randl, K., Armengol, N.L., Mondrejevski, L., and Miliou, I. (2023). Early prediction of the risk of icu mortality with deep federated learning. In 2023 IEEE 36th International Symposium on Computer-Based Medical Systems (CBMS) (IEEE), pp. 706–711.
20. Reyna, M.A., Josef, C.S., Jeter, R., Shashikumar, S.P., Westover, M.B., Nemati, S., Clifford, G.D., and Sharma, A. (2020). Early prediction of sepsis from clinical data: the physionet/computing in cardiology challenge 2019. Crit. Care Med. *48*, 210–217.
21. Ma, L., Ma, X., Gao, J., Zhang, C., Yu, Z., Jiao, X., Ruan, W., Wang, Y., Tang, W., and Wang, J. (2020). Covidcare: Transferring knowledge from existing emr to emerging epidemic for interpretable prognosis. Preprint at arXiv. https://doi.org/10.48550/arXiv.2007.08848.
22. Gao, J., Xiao, C., Glass, L.M., and Sun, J. (2020). Dr. agent: Clinical predictive model via mimicked second opinions. J. Am. Med. Inf. Assoc. *27*, 1084–1091.
23. Zhu, Y., Wang, W., Gao, J., and Ma, L.P.E.H.R. (2024). A Predictive Modeling Toolkit for Electronic Health Records. https://doi.org/10.5281/zenodo.10573567.
24. Zhu, Y., Wang, W., Gao, J., and Ma, L. (2024). Covid-19 ehr benchmark - online platform. https://doi.org/10.5281/zenodo.10573647.
25. Hospitales, H. (2020). Covid Data Save Lives. https://www.hmhospitales.com/prensa/notas-de-prensa/comunicado-covid-data-save-lives.
26. Ma, L., Ma, X., Gao, J., Jiao, X., Yu, Z., Zhang, C., Ruan, W., Wang, Y., Tang, W., and Wang, J. (2021). Distilling knowledge from publicly available online emr data to emerging epidemic for prognosis. In Proceedings of the Web Conference 2021, pp. 3558–3568.
27. Ma, L., Gao, J., Wang, Y., Zhang, C., Wang, J., Ruan, W., Tang, W., Gao, X., and Ma, X. (2020). Adacare: Explainable clinical health status representation learning via scale-adaptive feature extraction and recalibration.

CellPress
OPEN ACCESS

Proc. AAAI Conf. Artif. Intell. *34*, 825–832. https://doi.org/10.1609/aaai.v34i01.5427.

28. Ma, L., Zhang, C., Wang, Y., Ruan, W., Wang, J., Tang, W., Ma, X., Gao, X., and Gao, J. (2020). Concare: Personalized clinical feature embedding via capturing the healthcare context. Proc. AAAI Conf. Artif. Intell. *34*, 833–840. https://doi.org/10.1609/aaai.v34i01.5428.
29. Knight, S.R., Ho, A., Pius, R., Buchan, I., Carson, G., Drake, T.M., Dunning, J., Fairfield, C.J., Gamble, C., Green, C.A., et al. (2020). Risk stratification of patients admitted to hospital with covid-19 using the isaric who clinical characterisation protocol: development and validation of the 4c mortality score. bmj *370*, m3339. https://doi.org/10.1136/bmj.m3339.
30. Chen, T., and Guestrin, C.X. (2016). A scalable tree boosting system. In Proceedings of the 22nd acm sigkdd international conference on knowledge discovery and data mining, pp. 785–794.
31. Dorogush, A.V., Ershov, V., and Gulin, A. (2018). Catboost: gradient boosting with categorical features support. Preprint at arXiv. https://doi.org/10.48550/arXiv.1810.11363.
32. Rumelhart, D.E., Hinton, G.E., and Williams, R.J. (1986). Learning representations by back-propagating errors. Nature *323*, 533–536.
33. Hochreiter, S., and Schmidhuber, J. (1997). Long short-term memory. Neural Comput. *9*, 1735–1780.
34. Chung, J., Gulcehre, C., Cho, K., and Bengio, Y. (2014). Empirical evaluation of gated recurrent neural networks on sequence modeling. Preprint at arXiv. https://doi.org/10.48550/arXiv.1412.3555.
35. Bai, S., Kolter, J.Z., and Koltun, V. (2018). An empirical evaluation of generic convolutional and recurrent networks for sequence modeling. Preprint at arXiv. https://doi.org/10.48550/arXiv.1803.01271.
36. Vaswani, A., Shazeer, N., Parmar, N., Uszkoreit, J., Jones, L., Gomez, A.N., Kaiser, L., and Polosukhin, I. (2017). Attention is all you need. In *Proceedings of the 31st International Conference on Neural Information Processing Systems*. NIPS'17 Red Hook (Curran Associates Inc.), pp. 6000–6010.
37. Choi, E., Bahadori, M.T., Kulas, J.A., Schuetz, A., Stewart, W.F., and Sun, J. (2016). Retain: an interpretable predictive model for healthcare using reverse time attention mechanism. In *Proceedings of the 30th International Conference on Neural Information Processing Systems*. NIPS'16 Red Hook (Curran Associates Inc.), pp. 3512–3520.
38. Gao, J., Xiao, C., Wang, Y., Tang, W., Glass, L.M., and Sun, J.S. (2020). Stage-aware neural networks for health risk prediction. Proceedings of The Web Conference *2020*, 530–540.
39. Zhang, C., Gao, X., Ma, L., Wang, Y., Wang, J., and Tang, W. (2021). Grasp: Generic framework for health status representation learning based on incorporating knowledge from similar patients. Proc. AAAI Conf. Artif. Intell. *35*, 715–723. https://doi.org/10.1609/aaai.v35i1.16152.
40. Dwivedi, A.K., Mallawaarachchi, I., and Alvarado, L.A. (2017). Analysis of small sample size studies using nonparametric bootstrap test with pooled resampling method. Stat. Med. *36*, 2187–2205.
41. Mallett, S., Halligan, S., Thompson, M., Collins, G.S., and Altman, D.G. (2012). Interpreting diagnostic accuracy studies for patient care. BMJ *345*, e3999. https://doi.org/10.1136/bmj.e3999.
42. Clifford, G.D., Silva, I., Moody, B., and Mark, R. (2015). Reducing false arrhythmia alarms in the icu: The physionet/computing in cardiology challenge 2015. Comput. Cardiol. *42*, 273–276.
43. Johnson, A., Pollard, T., and Mark, R. (2016). Mimic-iii Clinical Database. PhysioNet. URL:. https://doi.org/10.13026/C2XW26 version 1.4.
44. Johnson, A., Bulgarelli, L., Pollard, T., Horng, S., Celi, L.A., and Mark, R. (2023). Mimic-iv. PhysioNet. URL:. https://doi.org/10.13026/s6n6-xd98 version 2.2.
45. Johnson, A.E.W., Bulgarelli, L., Shen, L., Gayles, A., Shammout, A., Horng, S., Pollard, T.J., Hao, S., Moody, B., Gow, B., et al. (2023). Mimic-iv, a freely accessible electronic health record dataset. Sci. Data *10*, 1.

Patterns, Volume *5*

Supplemental information

A comprehensive benchmark for COVID-19 predictive modeling using electronic health records in intensive care

Junyi Gao, Yinghao Zhu, Wenqing Wang, Zixiang Wang, Guiying Dong, Wen Tang, Hao Wang, Yasha Wang, Ewen M. Harrison, and Liantao Ma

Supplemental Experimental Procedures 1
Baseline Models Descriptions

Clinical Scoring model

- **4C mortality score**[1] is a risk stratification tool that predict in-hospital mortality or in-hospital clinical deterioration (defined as any requirement of ventilatory support or critical care, or death) for hospitalised COVID-19 patients. They are designed to require only parameters that are commonly available at hospital presentation.

Machine learning models

- **Decision tree (DT)** is a non-parametric supervised learning algorithm with a hierarchical tree structure. DT has been widely applied in many clinical predictive tasks, such as mortality prediction for peritoneal dialysis patients[2].

- **Random forest (RF)** is a supervised ensemble learning method for classification, regression and other tasks. It constructs a multitude of decision trees during the training period. RF has been used to predict the patients' severity of the COVID-19 case and possible mortality outcome, recovery or death[3].

- **Gradient Boosting Decision Tree (GBDT)** is a widely-used algorithm for solving prediction problems in both classification and regression tasks. GBDT takes decision trees as the base learner and determines final prediction results based on a series of DTs' outputs. GBDT exhibits good performance in performing COVID-19 mortality prediction tasks with structured clinical data[4].

- **XGBoost**[5] is a recursive tree-based supervised machine learning classifier. XGBoost has been used to predict the mortality for COVID-19 infected patients[6].

- **CatBoost**[7] is also a gradient boost machine learning framework. It builds a symmetric tree and uses an ordered boosting strategy with a faster training and prediction speed compared to XGBoost. Catboost has been used to predict and detect the number of confirmed and death cases of COVID-19[8].

Basic deep learning models

- **Multi-layer perceptron (MLP)** is the feed-forward-based neural network. MLP has been used to predict acute kidney injury[9].

- **Recurrent neural network (RNN)**[10] is the most popular framework to learn the abstract embedding of variable-length time series. RNN has been widely used to predict the risk of the first episode of psychosis or heart failure[11,12].

- **Long-short term memory network (LSTM)**[13] is a variant of the Recurrent Neural Network (RNN), capable of learning long-term dependencies. LSTM has been used to perform the 90-day all-cause mortality in the intensive care unit (ICU), based on the concatenated static features and dynamic features[14].

- **Gated recurrent units (GRU)**[15] embeds the time series as the input to perform the target prediction. It is a widely applied variant of the Recurrent Neural Network (RNN), which improves the capability to maintain historical memories and reduces parameters in the update and reset gates. GRU has been used to predict several severe complications (mortality, renal failure with a need for renal replacement therapy, and postoperative bleeding leading to operative revision) in post-cardiosurgical care in real-time[16].

- **Temporal convolutional networks (TCN)** [17] is a generic temporal convolutional network architecture for sequence modeling. TCN has been used to forecast hospital resource utilization (i.e., the number of hospital beds and ventilators) for COVID-19 patients[18].
- **Transformer**[19] in this experiment is the encoder part of the original Transformer, which comprises the positional encoding module and the self-attention module. The transformer has been used to perform the mortality risk analysis for liver transplant recipients[20].

EHR-specific predictive models

- **RETAIN**[21] is the deep-based Reverse Time Attention model for analyzing EHR data. It utilizes a two-level neural attention module to attend important clinical visits and features. RETAIN has been used to predict heart failure by taking previous diagnoses (categorical variables) as features. In our benchmark, we modify the input layer of RETAIN with multi-layer perceptron (MLP) to make it capable of using numerical variables as features.
- **StageNet**[22] is the Stage-aware neural Network, which extracts disease stage information from EHR and integrates it into risk prediction. This model comprises a stage-aware LSTM module that extracts health stage variations unsupervisedly and a stage-adaptive convolutional module that incorporates stage-related progression patterns. StageNet has been used to perform the decompensation prediction for ICU patients in the MIMIC-III dataset and the mortality prediction of End-Stage Renal Disease (ESRD) patients in Peking University Third Hospital.
- **Dr. Agent**[23] augments RNN with 2 policy gradient agents. It learns a dynamic skip connection to focus on the relevant information over time. Dr. Agent has been used to perform the in-hospital mortality prediction, acute care phenotype classification, physiologic decompensation prediction and length of stay forecasting task on the MIMIC-III (Medical Information Mart for Intensive Care) dataset.
- **AdaCare**[24] employs a multi-scale adaptive dilated convolutional module to capture the long and short-term variations of biomarkers to depict the health status in multiple time scales. AdaCare has been used to perform decompensation prediction on the MIMIC-III dataset and the mortality prediction on the End-Stage Renal Disease (ESRD) dataset.
- **ConCare**[25] employs multi-channel embedding architecture and self-attention mechanism to model the relationship of feature sequences and build the health representation. ConCare has been used to perform the morality prediction on the MIMIC-III and ESRD datasets.
- **GRASP**[26] is a generic framework for healthcare models, which leverages the information extracted from patients with similar conditions to enhance the cohort representation learning results. GRASP has been used to perform the sepsis prediction on the cardiology dataset and mortality prediction on the ESRD dataset.

Supplemental Experimental Procedures 2
Experiment Environments and Model Hyperparameters

For DT, RF, GBDT models, we use the scikit-learn package (version 1.2.2), XGBoost (version 1.7.5), CatBoost (version 1.2) to construct and train the models. For deep learning models, we use the PyTorch (version 2.0.1) and PyTorch Lighting (version 2.0.2) to implement the models. We use the mini-batch gradient descent to train the models and the batch size is set to 64 for TJH dataset and 128 for CDSL dataset. The ζ and η are set to 0.1. For the metric calculations, E is

the maximum value at the 95% percentile of the length-of-stay of all patient in both CDSL and TJH datasets. In the hold-out set, E is 27.25 in the CDSL dataset and 21.44 in the TJH dataset. We use AdamW optimizer with tuned learning rate to train the models over 100 epochs. The model training process will be stopped if the score on the validation set do not improve over 10 consecutive iterations. We set the random seed to 0 in all computational runs.

Experiments are conducted on a server equipped with dual Intel Xeon Silver 4210R CPUs, each with 10 cores supporting 20 threads, two NVIDIA RTX 3090 GPUs and 64GB RAM. Notably, all experiments are executed solely on one of the GPUs for consistent results.

The model hyperparameters are shown in Table S1, Table S2, and S3. To perform the 4C calculations, we followed the guidelines and methodology provided at `https://isaric4c.net/risk/4c/`. Also, we have incorporated additional details about the 4C mortality score in the main text. The computation of the 4C mortality score is a straightforward process. Each patient in our study is evaluated based on a set of variables — four variables in the TJH dataset and five in the CDSL dataset. For each variable, a specific score is assigned, reflecting the patient's status in that variable. The total 4C mortality score for a patient is the sum of these individual scores. Each level of the accumulated score correlates with a distinct probability of mortality. We have presented the variables used for calculating the 4C mortality scores in both datasets in Table S4.

Table S1: *Hyperparameter settings of machine learning models.* All hyperparameters are obtained using grid-search on the validation set.

Dataset	Task	Model	Detail
TJH	outcome	RF	max depth 10, n_estimators 100, learning rate 0.1, criterion gini
	length of stay	RF	max depth 15, n_estimators 50, learning rate 1.0, criterion squared_error
	outcome	DT	max depth 10, min_samples_split 2, min_samples_leaf 1
	length of stay	DT	max depth 5, criterion squared_error, min_samples_split 2
	outcome	GBDT	max depth 5, n_estimators 100, learning rate 0.1, subsample 1.0
	length of stay	GBDT	max depth 5, n_estimators 100, learning rate 0.1, subsample 1.0
	outcome	XGBoost	max depth 5, n_estimators 100, learning rate 0.1, objective binary:logistic
	length of stay	XGBoost	max depth 5, n_estimators 100, learning rate 0.1, objective reg:squarederror
	outcome	CatBoost	max depth 5, n_estimators 100, learning rate 0.1, loss CrossEntropy
	length of stay	CatBoost	max depth 5, n_estimators 100, learning rate 0.1, loss RMSE
CDSL	outcome	RF	max depth 15, n_estimators 100, learning rate 1.0, criterion gini
	length of stay	RF	max depth 15, n_estimators 100, learning rate 1.0, criterion squared_error
	outcome	DT	max depth 5, min_samples_split 2, min_samples_leaf 1
	length of stay	DT	max depth 5, criterion squared_error, min_samples_split 2
	outcome	GBDT	max depth 5, n_estimators 100, learning rate 0.1, subsample 1.0
	length of stay	GBDT	max depth 5, n_estimators 100, learning rate 0.1, subsample 1.0
	outcome	XGBoost	max depth 5, n_estimators 50, learning rate 0.1, objective binary:logistic
	length of stay	XGBoost	max depth 10, n_estimators 50, learning rate 0.1, objective reg:squarederror
	outcome	CatBoost	max depth 5, n_estimators 50, learning rate 0.1, loss CrossEntropy
	length of stay	CatBoost	max depth 10, n_estimators 100, learning rate 0.1, loss RMSE

Supplemental Experimental Procedures 3
Evaluation of Prediction Fairness

To evaluate the models' fairness, we test the GRU model using fairness experiments. The fairness metrics include disparate impact (DI)[27], average odds difference (AOD)[28], equal opportunity difference (EOD)[28] and statistical parity difference (SPD)[29]. The results are shown in Table S5.

- **Disparate Impact (DI)[27]:** DI measures the ratio of the positive outcome rates between the unprivileged and privileged groups. The ideal value is 1.0, indicating equal positive outcome

Table S2: *Hyperparameter settings of deep learning models in the TJH dataset.* All hyperparameters are obtained using grid-search on the validation set.

Task	Model	Detail
outcome	MLP	hidden dim 32, learning rate 0.01, feed-forward dim 128
length of stay	MLP	hidden dim 128, learning rate 0.001, feed-forward dim 512
multi-task	MLP	hidden dim 32, learning rate 0.001, feed-forward dim 128
outcome	RNN	hidden dim 64, learning rate 0.001, layers 1
length of stay	RNN	hidden dim 64, learning rate 0.01, layers 1
multi-task	RNN	hidden dim 64, learning rate 0.01, layers 1
outcome	LSTM	hidden dim 128, learning rate 0.01, layers 1
length of stay	LSTM	hidden dim 128, learning rate 0.01, layers 1
multi-task	LSTM	hidden dim 128, learning rate 0.01, layers 1
outcome	GRU	hidden dim 32, learning rate 0.01, layers 1
length of stay	GRU	hidden dim 128, learning rate 0.01, layers 1
multi-task	GRU	hidden dim 32, learning rate 0.0001, layers 1
outcome	TCN	hidden dim 64, learning rate 0.0001, kernel size 2, dropout rate 0.2
length of stay	TCN	hidden dim 64, learning rate 0.01, kernel size 2, dropout rate 0.2
multi-task	TCN	hidden dim 128, learning rate 0.01, kernel size 2, dropout rate 0.2
outcome	Transformer	hidden dim 64, learning rate 0.0001, layers 1, heads 1
length of stay	Transformer	hidden dim 32, learning rate 0.001, layers 1, heads 1
multi-task	Transformer	hidden dim 32, learning rate 0.001, layers 1, heads 1
outcome	RETAIN	hidden dim 64, learning rate 0.001, dropout rate 0.1
length of stay	RETAIN	hidden dim 64, learning rate 0.001, dropout rate 0.1
multi-task	RETAIN	hidden dim 64, learning rate 0.001, dropout rate 0.1
outcome	StageNet	hidden dim 64, learning rate 0.0001, levels 3, chunk size 64
length of stay	StageNet	hidden dim 128, learning rate 0.01, levels 3, chunk size 128
multi-task	StageNet	hidden dim 32, learning rate 0.001, levels 3, chunk size 32
outcome	Dr. Agent	hidden dim 32, learning rate 0.01, actions 10, units 64
length of stay	Dr. Agent	hidden dim 64, learning rate 0.01, actions 10, units 64
multi-task	Dr. Agent	hidden dim 64, learning rate 0.01, actions 10, units 64
outcome	AdaCare	hidden dim 32, learning rate 0.0001, kernel size 2, kernel num 64, RNN type GRU
length of stay	AdaCare	hidden dim 64, learning rate 0.01, kernel size 2, kernel num 64, RNN type GRU
multi-task	AdaCare	hidden dim 64, learning rate 0.01, kernel size 2, kernel num 64, RNN type GRU
outcome	GRASP	hidden dim 128, learning rate 0.01, block GRU, clusters 12, dropout rate 0.5
length of stay	GRASP	hidden dim 64, learning rate 0.01, block GRU, clusters 12, dropout rate 0.5
multi-task	GRASP	hidden dim 32, learning rate 0.01, block GRU, clusters 12, dropout rate 0.5
outcome	ConCare	hidden dim 64, learning rate 0.001, GRU layers 1, attention None
length of stay	ConCare	hidden dim 64, learning rate 0.001, GRU layers 1, attention None
multi-task	ConCare	hidden dim 64, learning rate 0.001, GRU layers 1, attention None

Table S3: *Hyperparameter settings of deep learning models in the CDSL dataset.* All hyperparameters are obtained using grid-search on the validation set.

Task	Model	Detail
outcome	MLP	hidden dim 32, learning rate 0.001, feed-forward dim 128
length of stay	MLP	hidden dim 64, learning rate 0.001, feed-forward dim 256
multi-task	MLP	hidden dim 128, learning rate 0.001, feed-forward dim 512
outcome	RNN	hidden dim 128, learning rate 0.001, layers 1
length of stay	RNN	hidden dim 128, learning rate 0.01, layers 1
multi-task	RNN	hidden dim 32, learning rate 0.01, layers 1
outcome	LSTM	hidden dim 64, learning rate 0.001, layers 1
length of stay	LSTM	hidden dim 32, learning rate 0.0001, layers 1
multi-task	LSTM	hidden dim 32, learning rate 0.001, layers 1
outcome	GRU	hidden dim 64, learning rate 0.01, layers 1
length of stay	GRU	hidden dim 64, learning rate 0.01, layers 1
multi-task	GRU	hidden dim 32, learning rate 0.01, layers 1
outcome	TCN	hidden dim 128, learning rate 0.01, kernel size 2, dropout rate 0.2
length of stay	TCN	hidden dim 128, learning rate 0.001, kernel size 2, dropout rate 0.2
multi-task	TCN	hidden dim 128, learning rate 0.001, kernel size 2, dropout rate 0.2
outcome	Transformer	hidden dim 32, learning rate 0.01, layers 1, heads 1
length of stay	Transformer	hidden dim 128, learning rate 0.01, layers 1, heads 1
multi-task	Transformer	hidden dim 128, learning rate 0.01, layers 1, heads 1
outcome	RETAIN	hidden dim 64, learning rate 0.01, dropout rate 0.1
length of stay	RETAIN	hidden dim 128, learning rate 0.001, dropout rate 0.1
multi-task	RETAIN	hidden dim 128, learning rate 0.01, dropout rate 0.1
outcome	StageNet	hidden dim 32, learning rate 0.001, levels 3, chunk size 32
length of stay	StageNet	hidden dim 128, learning rate 0.001, levels 3, chunk size 128
multi-task	StageNet	hidden dim 32, learning rate 0.0001, levels 3, chunk size 32
outcome	Dr. Agent	hidden dim 64, learning rate 0.001, actions 10, units 64
length of stay	Dr. Agent	hidden dim 32, learning rate 0.01, actions 10, units 64
multi-task	Dr. Agent	hidden dim 32, learning rate 0.01, actions 10, units 64
outcome	AdaCare	hidden dim 32, learning rate 0.01, kernel size 2, kernel num 64, RNN type GRU
length of stay	AdaCare	hidden dim 128, learning rate 0.001, kernel size 2, kernel num 64, RNN type GRU
multi-task	AdaCare	hidden dim 64, learning rate 0.01, kernel size 2, kernel num 64, RNN type GRU
outcome	GRASP	hidden dim 32, learning rate 0.01, block GRU, clusters 12, dropout rate 0.5
length of stay	GRASP	hidden dim 32, learning rate 0.001, block GRU, clusters 12, dropout rate 0.5
multi-task	GRASP	hidden dim 128, learning rate 0.001, block GRU, clusters 12, dropout rate 0.5
outcome	ConCare	hidden dim 64, learning rate 0.001, GRU layers 1, attention None
length of stay	ConCare	hidden dim 64, learning rate 0.001, GRU layers 1, attention None
multi-task	ConCare	hidden dim 64, learning rate 0.001, GRU layers 1, attention None

rates for both groups. A value less than 1 indicates that the unprivileged group is less likely to receive the positive outcome than the privileged group. This metric is given by:

$$\text{DI} = \frac{P(Y = 1|D = \text{unprivileged})}{P(Y = 1|D = \text{privileged})}$$

Where: Y is the outcome variable (e.g., dead or alive) and D represents the group (privileged or unprivileged). For the purpose of our study, the privileged groups are identified as male and younger patients.

- **Average Odds Difference (AOD)[28]:** AOD measures the average difference of FPR and TPR for the unprivileged and privileged groups. The ideal value is 0, indicating both groups have equal FPR and TPR. The formula for AOD is:

$$\text{AOD} = \frac{1}{2}[(FPR_{D=\text{unprivileged}} - FPR_{D=\text{privileged}}) + (TPR_{D=\text{unprivileged}} - TPR_{D=\text{privileged}})]$$

Table S4: *Variables employed in the 4C mortality score calculation for both TJH and CDSL datasets.*

TJH	CDSL	4C Mortality Score Component
Sex	Sex	Sex at birth
Age	Age	Age
Urea	Urea	Urea
CRP	CRP	C reactive protein
-	SO2C	Peripheral oxygen saturation on room air

- **Equal Opportunity Difference (EOD)[28]:** EOD is similar to AOD but only considers the TPR. The ideal value is 0, indicating both groups have equal TPR. EOD is computed as:

$$\text{EOD} = TPR_{D=\text{unprivileged}} - TPR_{D=\text{privileged}}$$

- **Statistical Parity Difference (SPD)[29]:** SPD measures the difference in the probability of positive decisions between the unprivileged and privileged groups. The ideal value is 0, indicating both groups have equal probabilities of receiving a positive decision. SPD can be calculated using:

$$\text{SPD} = P(Y = 1|D = \text{unprivileged}) - P(Y = 1|D = \text{privileged})$$

The results in Table S5 show that most model predictions are more fair on the CDSL dataset than the TJH dataset, especially in terms of sex. This may be because the population size is limited in the TJH dataset and thus has induced population bias. Future work may include conducting a more comprehensive analysis to improve the bias and fairness of different predicting models.

Supplemental Experimental Procedures 4 Benchmarking Experiment under Hold-Out Data Split Settings

We employ a 10-fold cross-validation strategy to mitigate the bias associated with small sample sizes. Randomly splitting the test set can lead to unstable model performance due to its limited size. Cross-validation allows the model to be tested on all samples, thereby yielding more consistent performance conclusions. However, to evaluate the model's generalizability over time more comprehensively, we conduct a standard holdout experiment. We divide the dataset into training, validation, and test sets in a 7:1:2 ratio, based on admission times (using the latest 20% of patients as the holdout dataset, as recommended). We retrain all models five times with different random seeds and report the standard deviations. The model performances are detailed in Table S6 and Table S7.

For outcome-specific LOS prediction on the TJH dataset, AdaCare achieves the lowest MAE and MSE. On the CDSL dataset, TCN, AdaCare, and RNN exhibit the lowest MAE, MSE, and OSMAE. In the early mortality prediction task, the results align closely with those from the cross-validation setting. AdaCare and ConCare demonstrate superior performance in AUROC, AUPRC, and ES on the TJH dataset. Meanwhile, ConCare, StageNet, and GBDT achieve the best performance on the CDSL dataset. We observe significant variability in some model performances, particularly in MSE on the TJH dataset, attributable mainly to its smaller size and consequent less stable conclusions compared to the cross-validation setting. Furthermore, we note that

Table S5: *Comprehensive fairness assessment across deep learning models on TJH and CDSL datasets.* The table presents a detailed evaluation of fairness metrics, including Disparate Impact (DI), Average Odds Difference (AOD), Equal Opportunity Difference (EOD), and Statistical Parity Difference (SPD), for multiple deep learning models (e.g., MLP) across different privileged groups (Sex, Age).

Dataset		TJH				CDSL			
Metric		DI (↑)	AOD (↓)	EOD (↓)	SPD (↓)	DI (↑)	AOD (↓)	EOD (↓)	SPD (↓)
MLP	Sex	0.80±0.07	0.01±0.02	0.01±0.04	0.09±0.04	0.84±0.04	0.02±0.01	0.04±0.02	0.01±0.00
	Age	0.78±0.05	0.02±0.01	0.04±0.02	0.09±0.02	0.82±0.02	0.02±0.01	0.03±0.01	0.01±0.00
RNN	Sex	0.81±0.09	0.01±0.03	0.00±0.05	0.09±0.06	0.88±0.05	0.02±0.01	0.04±0.02	0.01±0.01
	Age	0.76±0.07	0.03±0.02	0.05±0.03	0.10±0.03	0.80±0.04	0.03±0.01	0.06±0.02	0.02±0.00
LSTM	Sex	0.82±0.09	0.00±0.03	0.02±0.03	0.09±0.06	0.89±0.06	0.02±0.01	0.03±0.03	0.01±0.01
	Age	0.79±0.06	0.02±0.02	0.03±0.02	0.09±0.02	0.81±0.03	0.03±0.01	0.06±0.02	0.02±0.00
GRU	Sex	0.81±0.10	0.01±0.04	0.01±0.06	0.10±0.07	0.89±0.06	0.01±0.01	0.02±0.03	0.01±0.01
	Age	0.76±0.07	0.03±0.02	0.05±0.03	0.10±0.02	0.82±0.05	0.03±0.01	0.06±0.02	0.02±0.00
TCN	Sex	0.81±0.09	0.00±0.03	0.00±0.05	0.09±0.06	0.88±0.07	0.02±0.01	0.05±0.03	0.01±0.01
	Age	0.76±0.06	0.03±0.01	0.06±0.03	0.10±0.02	0.79±0.03	0.03±0.01	0.06±0.02	0.02±0.00
Transformer	Sex	0.83±0.09	0.00±0.03	0.02±0.03	0.09±0.06	0.86±0.05	0.01±0.01	0.01±0.03	0.00±0.01
	Age	0.80±0.06	0.01±0.01	0.02±0.02	0.09±0.02	0.79±0.05	0.02±0.01	0.04±0.02	0.01±0.01
RETAIN	Sex	0.79±0.09	0.01±0.02	0.00±0.04	0.10±0.06	0.85±0.08	0.02±0.01	0.05±0.03	0.01±0.01
	Age	0.76±0.06	0.02±0.02	0.05±0.03	0.10±0.02	0.77±0.02	0.03±0.01	0.06±0.02	0.01±0.00
StageNet	Sex	0.80±0.10	0.01±0.03	0.00±0.06	0.10±0.07	0.89±0.06	0.02±0.01	0.03±0.02	0.01±0.01
	Age	0.76±0.07	0.03±0.02	0.06±0.03	0.10±0.03	0.81±0.04	0.03±0.01	0.06±0.02	0.02±0.01
Dr. Agent	Sex	0.80±0.09	0.01±0.02	0.01±0.03	0.10±0.06	0.86±0.06	0.02±0.02	0.03±0.04	0.01±0.01
	Age	0.79±0.05	0.01±0.01	0.03±0.01	0.09±0.02	0.82±0.06	0.03±0.01	0.06±0.02	0.02±0.01
AdaCare	Sex	0.79±0.10	0.01±0.02	0.01±0.04	0.10±0.06	0.89±0.06	0.02±0.01	0.04±0.02	0.01±0.01
	Age	0.77±0.06	0.02±0.01	0.03±0.03	0.09±0.03	0.83±0.03	0.03±0.01	0.05±0.02	0.01±0.00
GRASP	Sex	0.81±0.09	0.01±0.03	0.01±0.04	0.10±0.06	0.90±0.08	0.01±0.01	0.02±0.02	0.01±0.01
	Age	0.79±0.06	0.02±0.01	0.04±0.02	0.09±0.02	0.79±0.04	0.03±0.01	0.06±0.02	0.02±0.01
ConCare	Sex	0.79±0.08	0.01±0.02	0.00±0.04	0.10±0.05	0.88±0.06	0.03±0.01	0.05±0.03	0.01±0.01
	Age	0.77±0.06	0.02±0.01	0.04±0.02	0.10±0.02	0.78±0.05	0.03±0.01	0.06±0.02	0.02±0.00

overall model performance on the hold-out dataset is inferior to that in the cross-validation setting, suggesting a shift in dataset distribution over time and a corresponding decline in the models' predictive capabilities. Overall, AdaCare, Dr. Agent and StageNet are top-performing models for both tasks on both datasets. For a light-weight choice, GBDT outperformed other machine learning models and could be considered as the choice for machine learning models.

Supplemental Experimental Procedures 5 Benchmarking Experiment on MIMIC Datasets

To enhance the comprehensiveness and validity of the evaluation, we include a broader array of ICU datasets (MIMIC-III[30] and MIMIC-IV[31]). We conduct the in-hospital mortality prediction task on the two datasets. The label definition and data preprocessing are following previous benchmark works[32], but we provide a more comprehensive baseline comparison. The results are shown in Table S8.

We find that Dr. Agent and StageNet achieve better performance on the MIMIC-III dataset, while Dr. Agent and GRU achieve better performance on the MIMIC-IV dataset. This conclusion

Table S6: *Benchmarking performance of outcome-specific length-of-stay prediction on TJH and CDSL hold-out test sets.*. The reported score is in the form of $mean \pm std$. Subscript m signifies a multi-task learning strategy, while subscript t indicates a two-stage learning strategy. **Bold** denotes the best performance. Underline indicates that the multi-task setting outperforms the two-stage learning strategy. The asterisk * denotes that the performance improvement against the two-stage model is statistically significant (p-value < 0.05).

Dataset	TJH			CDSL		
Metric	MAE(↓)	MSE(↓)	OSMAE(↓)	MAE(↓)	MSE(↓)	OSMAE(↓)
RF_t	5.81 ± 0.18	45.89 ± 2.03	8.18 ± 0.19	4.21 ± 0.00	40.47 ± 0.05	4.24 ± 0.01
DT_t	5.00 ± 0.06	51.22 ± 1.83	9.34 ± 0.54	4.34 ± 0.00	41.82 ± 0.00	4.43 ± 0.00
$GBDT_t$	5.98 ± 0.06	50.39 ± 1.40	**7.21 ± 0.16**	4.24 ± 0.00	40.58 ± 0.02	4.27 ± 0.01
$CatBoost_t$	5.51 ± 0.14	41.25 ± 2.12	7.71 ± 0.33	4.22 ± 0.00	40.49 ± 0.08	4.24 ± 0.01
$XGBoost_t$	5.84 ± 0.00	46.86 ± 0.00	7.87 ± 0.00	4.22 ± 0.00	40.57 ± 0.00	4.23 ± 0.00
MLP_t	5.98 ± 0.27	51.09 ± 5.21	9.77 ± 1.18	4.22 ± 0.02	40.52 ± 0.04	4.20 ± 0.02
MLP_m	6.35 ± 0.24	71.81 ± 17.97	9.19 ± 0.27*	4.22 ± 0.04	40.14 ± 0.27*	4.19 ± 0.07
RNN_t	5.56 ± 0.11	41.27 ± 1.42	8.73 ± 0.45	4.08 ± 0.03	41.10 ± 1.42	**4.00 ± 0.05**
RNN_m	7.81 ± 0.33	87.99 ± 9.73	12.14 ± 0.78	4.11 ± 0.06	**39.25 ± 0.61***	4.15 ± 0.15
$LSTM_t$	5.28 ± 0.57	40.00 ± 11.06	8.65 ± 0.60	4.06 ± 0.02	39.71 ± 0.14	4.06 ± 0.04
$LSTM_m$	6.71 ± 0.48	70.72 ± 9.57	10.94 ± 1.12	4.18 ± 0.07	40.33 ± 0.60	4.25 ± 0.10
GRU_t	5.87 ± 0.31	47.79 ± 5.05	8.80 ± 0.70	4.08 ± 0.07	40.57 ± 2.20	4.03 ± 0.09
GRU_m	5.01 ± 0.29*	32.03 ± 3.06*	9.44 ± 1.47	4.17 ± 0.03	40.28 ± 1.63*	4.29 ± 0.09
TCN_t	6.29 ± 0.84	77.96 ± 34.06	9.53 ± 0.93	4.08 ± 0.06	40.38 ± 0.27	4.12 ± 0.24
TCN_m	7.50 ± 1.16	162.34 ± 108.99	11.93 ± 1.23	**4.04 ± 0.03***	39.39 ± 0.41*	4.03 ± 0.07*
$Transformer_t$	6.38 ± 0.20	62.65 ± 12.11	11.11 ± 0.91	4.14 ± 0.03	41.23 ± 0.55	4.60 ± 0.25
$Transformer_m$	6.46 ± 0.19	74.44 ± 26.43	11.27 ± 1.35	4.16 ± 0.06	43.27 ± 1.13	4.54 ± 0.27
$RETAIN_t$	5.36 ± 0.33	39.42 ± 5.26	12.60 ± 2.19	4.17 ± 0.08	40.87 ± 0.35	4.24 ± 0.09
$RETAIN_m$	6.30 ± 0.57	115.36 ± 72.40	12.63 ± 1.56	4.11 ± 0.02*	39.41 ± 0.49*	4.35 ± 0.15
$StageNet_t$	5.49 ± 0.85	47.51 ± 22.86	9.45 ± 0.46	4.08 ± 0.03	40.75 ± 1.04	4.14 ± 0.08
$StageNet_m$	5.10 ± 0.43*	33.51 ± 6.01*	15.09 ± 7.87	4.09 ± 0.02	40.40 ± 0.43	4.16 ± 0.02
Dr. $Agent_t$	6.36 ± 0.29	57.96 ± 3.57	9.69 ± 0.84	4.09 ± 0.03	41.17 ± 1.03	4.28 ± 0.20
Dr. $Agent_m$	7.06 ± 0.43	72.02 ± 2.90	10.15 ± 1.17	4.13 ± 0.09	39.83 ± 0.95*	4.22 ± 0.19
$AdaCare_t$	**3.58 ± 0.66**	**19.65 ± 5.81**	9.26 ± 1.58	**4.04 ± 0.01**	40.37 ± 0.86	4.10 ± 0.08
$AdaCare_m$	5.13 ± 0.76	34.44 ± 8.48	16.89 ± 8.79	4.12 ± 0.19	43.40 ± 4.03	4.11 ± 0.12
$GRASP_t$	5.40 ± 0.53	40.75 ± 10.74	8.94 ± 0.74	4.20 ± 0.07	41.00 ± 0.20	4.29 ± 0.10
$GRASP_m$	5.80 ± 0.90	47.69 ± 18.62	11.91 ± 4.34	4.18 ± 0.06	41.03 ± 0.93	4.31 ± 0.09
$ConCare_t$	4.79 ± 0.44	29.92 ± 5.63	8.15 ± 0.22	4.16 ± 0.02	41.12 ± 0.45	4.08 ± 0.03
$ConCare_m$	7.62 ± 0.20	78.77 ± 6.50	10.24 ± 0.43	4.13 ± 0.16	39.36 ± 0.62*	4.12 ± 0.23

Table S7: *Benchmarking performance on the task of early mortality prediction on TJH and CDSL hold-out test sets.* The reported score is of the form $mean \pm std$. 'TA' denotes the model trained with the time-aware loss. **Bold** denotes the best performance. Underline indicates that the model with time-aware loss outperforms the original model. The asterisk * denotes that the performance improvement against the model without TA version is statistically significant (p-value < 0.05). All three metrics are multiplied by 100 for readability purposes.

Dataset	TJH			CDSL		
Metric	AUPRC(↑)	AUROC(↑)	ES(↑)	AUPRC(↑)	AUROC(↑)	ES(↑)
RF	99.48 ± 0.04	98.13 ± 0.19	52.16 ± 2.32	52.16 ± 2.32	83.28 ± 0.21	-8.08 ± 0.30
DT	94.31 ± 0.80	85.68 ± 1.80	44.84 ± 3.33	27.71 ± 0.00	76.58 ± 0.00	-4.47 ± 0.00
GBDT	99.34 ± 0.03	97.57 ± 0.11	50.80 ± 0.62	41.91 ± 0.28	**84.71 ± 0.04**	3.55 ± 0.35
CatBoost	99.38 ± 0.11	97.74 ± 0.39	67.62 ± 6.16	40.00 ± 0.36	84.17 ± 0.23	-6.91 ± 0.78
XGBoost	99.46 ± 0.00	98.17 ± 0.00	39.92 ± 0.00	40.32 ± 0.00	84.44 ± 0.00	-1.22 ± 0.00
MLP	99.03 ± 0.82	97.63 ± 0.98	69.16 ± 8.37	39.63 ± 0.79	83.41 ± 0.36	-3.68 ± 1.24
MLP-TA	98.75 ± 0.74	96.94 ± 1.01	<u>69.44 ± 6.61</u>	<u>40.46 ± 0.89</u>*	<u>83.49 ± 0.32</u>	<u>-2.26 ± 2.14</u>*
RNN	99.69 ± 0.14	98.94 ± 0.46	75.56 ± 4.28	41.91 ± 1.20	83.36 ± 0.90	5.44 ± 3.40
RNN-TA	<u>99.71 ± 0.13</u>*	<u>98.99 ± 0.44</u>*	<u>75.78 ± 4.04</u>	<u>42.65 ± 1.17</u>*	83.35 ± 0.63	<u>7.00 ± 5.62</u>*
LSTM	99.14 ± 0.14	97.50 ± 0.24	73.70 ± 1.79	39.76 ± 1.40	81.73 ± 0.77	10.56 ± 1.76
LSTM-TA	<u>99.16 ± 0.14</u>	<u>97.56 ± 0.23</u>	<u>74.15 ± 2.61</u>*	<u>40.77 ± 1.32</u>*	<u>82.39 ± 0.53</u>*	<u>13.03 ± 2.66</u>*
GRU	99.43 ± 0.25	98.07 ± 0.78	75.74 ± 3.88	41.43 ± 1.93	83.28 ± 0.90	5.38 ± 5.13
GRU-TA	<u>99.48 ± 0.20</u>	<u>98.21 ± 0.64</u>*	<u>76.42 ± 2.06</u>*	<u>42.18 ± 1.35</u>*	<u>83.64 ± 0.82</u>*	<u>9.01 ± 5.59</u>*
TCN	98.71 ± 0.23	96.84 ± 0.25	74.38 ± 1.29	39.64 ± 1.48	83.50 ± 0.44	4.17 ± 8.71
TCN-TA	<u>98.72 ± 0.20</u>	96.84 ± 0.22	73.70 ± 1.79	<u>40.61 ± 1.68</u>*	83.46 ± 0.44	3.45 ± 3.26
Transformer	99.06 ± 0.86	97.84 ± 1.23	64.22 ± 6.73	31.18 ± 4.86	80.04 ± 1.15	-1.93 ± 12.86
Transformer-TA	<u>99.53 ± 0.22</u>	<u>98.52 ± 0.56</u>	64.22 ± 6.81	<u>32.31 ± 2.44</u>*	<u>80.89 ± 1.22</u>*	<u>9.48 ± 6.30</u>*
RETAIN	97.23 ± 1.21	92.70 ± 2.90	50.39 ± 15.40	37.65 ± 2.17	81.84 ± 1.33	1.92 ± 9.56
RETAIN-TA	<u>97.50 ± 1.11</u>	<u>93.14 ± 2.82</u>	<u>52.66 ± 16.36</u>	<u>38.83 ± 2.30</u>*	<u>82.42 ± 1.65</u>*	<u>5.31 ± 9.79</u>*
StageNet	99.35 ± 0.26	97.94 ± 0.64	70.07 ± 2.59	42.08 ± 0.53	83.09 ± 0.36	12.88 ± 3.08
StageNet-TA	<u>99.38 ± 0.25</u>*	<u>98.06 ± 0.63</u>*	<u>70.98 ± 3.98</u>*	<u>42.51 ± 0.52</u>*	<u>83.17 ± 0.32</u>	<u>**14.06 ± 2.80**</u>*
Dr. Agent	99.35 ± 0.24	97.85 ± 0.57	72.57 ± 3.84	40.83 ± 1.18	82.98 ± 0.78	11.18 ± 6.92
Dr. Agent-TA	<u>99.37 ± 0.24</u>	<u>97.92 ± 0.57</u>	<u>73.47 ± 2.93</u>*	<u>41.87 ± 1.45</u>*	<u>83.07 ± 1.12</u>*	<u>12.46 ± 6.58</u>*
AdaCare	99.85 ± 0.13	99.49 ± 0.41	62.32 ± 10.08	40.78 ± 0.94	83.46 ± 0.12	8.27 ± 1.81
AdaCare-TA	<u>**99.87 ± 0.09**</u>*	<u>**99.53 ± 0.32**</u>*	<u>69.35 ± 4.41</u>*	<u>41.90 ± 1.15</u>*	<u>83.72 ± 0.28</u>*	<u>9.70 ± 1.59</u>*
GRASP	99.42 ± 0.16	98.19 ± 0.39	72.79 ± 4.63	33.13 ± 1.10	75.04 ± 0.74	-2.32 ± 6.00
GRASP-TA	<u>99.49 ± 0.22</u>	<u>98.40 ± 0.57</u>*	72.79 ± 4.90	<u>34.57 ± 1.12</u>*	<u>75.43 ± 1.20</u>	<u>1.92 ± 2.39</u>
ConCare	99.50 ± 0.20	98.24 ± 0.55	77.33 ± 2.82	43.12 ± 0.09	84.39 ± 0.08	-1.23 ± 5.16
ConCare-TA	99.49 ± 0.24	98.22 ± 0.66	<u>**78.91 ± 1.72**</u>*	<u>**43.47 ± 0.14**</u>*	84.28 ± 0.17	<u>2.55 ± 3.38</u>*

is similar to the CDSL and TJH dataset, which proves the generalizability of these models. Since the LOS information in the two MIMIC datasets has high variance, we do not report the OSMAE on these datasets. In future works, these metrics can be applied to more specific cohorts that have lower LOS variance.

Similar to the TJH and CDSL datasets, we also explore the early prediction setting on the MIMIC-III and MIMIC-IV datasets. As shown in Figure S1, the time-aware loss can effectively improve various models' prediction performance on more general datasets. The MIMIC dataset statistics used in our experiments are shown in Table S9 and S10.

Table S8: *Benchmarking performance on the task of early mortality prediction on MIMIC-III and MIMIC-IV hold-out test sets.* The reported score is of the form $mean \pm std$. 'TA' denotes the model trained with the time-aware loss. **Bold** denotes the best performance. Underline indicates that the model with time-aware loss outperforms the original model. The asterisk * denotes that the performance improvement against the model without TA version is statistically significant (p-value < 0.05). All three metrics are multiplied by 100 for readability purposes.

Dataset	MIMIC-III			MIMIC-IV		
Metric	AUPRC(↑)	AUROC(↑)	ES(↑)	AUPRC(↑)	AUROC(↑)	ES(↑)
RF	43.56 ± 0.24	81.94 ± 0.08	-1.58 ± 0.31	39.46 ± 0.21	80.58 ± 0.06	0.50 ± 0.23
DT	31.35 ± 0.16	70.65 ± 0.17	7.45 ± 0.20	29.56 ± 0.22	74.04 ± 0.22	9.49 ± 0.16
GBDT	45.73 ± 0.02	83.67 ± 0.00	11.80 ± 0.03	42.06 ± 0.04	81.80 ± 0.01	13.57 ± 0.05
CatBoost	45.44 ± 0.34	83.50 ± 0.08	6.72 ± 0.14	41.44 ± 0.11	81.66 ± 0.04	8.67 ± 0.27
XGBoost	45.73 ± 0.20	83.65 ± 0.04	10.68 ± 0.43	41.54 ± 0.00	81.76 ± 0.00	12.29 ± 0.00
MLP	45.01 ± 0.28	83.04 ± 0.07	12.73 ± 1.51	41.39 ± 0.54	81.35 ± 0.17	10.66 ± 1.29
MLP-TA	44.92 ± 0.57	82.86 ± 0.18	<u>13.68 ± 1.70</u>*	40.97 ± 0.35	81.18 ± 0.14	<u>13.80 ± 1.22</u>*
RNN	45.87 ± 0.61	83.34 ± 0.25	13.45 ± 0.96	44.08 ± 0.21	82.85 ± 0.10	15.93 ± 2.35
RNN-TA	45.38 ± 0.52	82.99 ± 0.20	<u>13.69 ± 1.12</u>	43.59 ± 0.34	82.59 ± 0.16	<u>**17.88 ± 1.81**</u>*
LSTM	47.46 ± 0.34	83.66 ± 0.06	15.35 ± 1.44	44.52 ± 0.49	83.14 ± 0.21	15.27 ± 1.73
LSTM-TA	47.14 ± 0.35	83.29 ± 0.15	<u>15.70 ± 1.32</u>	44.34 ± 0.44	82.86 ± 0.15	<u>17.37 ± 1.65</u>*
GRU	47.19 ± 0.45	83.80 ± 0.18	14.05 ± 0.70	**45.58 ± 0.18**	83.50 ± 0.09	15.43 ± 1.60
GRU-TA	46.87 ± 0.45	83.39 ± 0.19	<u>15.35 ± 2.62</u>*	45.33 ± 0.22	83.18 ± 0.14	<u>17.44 ± 2.03</u>*
TCN	47.71 ± 0.46	84.17 ± 0.13	**18.72 ± 1.85**	45.00 ± 0.51	**83.53 ± 0.23**	16.42 ± 2.34
TCN-TA	<u>47.73 ± 0.24</u>	84.04 ± 0.13	18.10 ± 2.31	<u>45.03 ± 0.59</u>	83.47 ± 0.20	<u>17.74 ± 1.75</u>*
Transformer	42.51 ± 0.49	82.43 ± 0.16	8.55 ± 1.88	42.32 ± 0.24	82.54 ± 0.16	10.12 ± 3.95
Transformer-TA	42.38 ± 0.54	82.24 ± 0.18	<u>9.68 ± 2.31</u>*	42.24 ± 0.36	82.54 ± 0.14	<u>13.77 ± 4.17</u>*
RETAIN	46.72 ± 0.44	83.61 ± 0.23	6.72 ± 1.94	44.89 ± 0.54	83.34 ± 0.26	6.65 ± 0.59
RETAIN-TA	46.49 ± 0.35	83.21 ± 0.15	<u>8.27 ± 1.86</u>*	44.48 ± 0.25	82.68 ± 0.22	<u>7.33 ± 1.19</u>*
StageNet	47.33 ± 0.38	83.60 ± 0.16	16.66 ± 2.23	44.79 ± 0.26	83.21 ± 0.35	15.62 ± 0.77
StageNet-TA	46.81 ± 0.41	83.22 ± 0.19	16.49 ± 1.26	44.22 ± 0.44	82.70 ± 0.33	<u>17.68 ± 3.12</u>*
Dr. Agent	47.94 ± 0.33	**84.30 ± 0.10**	15.71 ± 2.32	44.53 ± 0.69	83.15 ± 0.24	13.54 ± 1.53
Dr. Agent-TA	<u>**48.10 ± 0.23**</u>*	84.17 ± 0.13	<u>16.04 ± 2.59</u>*	44.00 ± 0.29	82.67 ± 0.15	<u>13.84 ± 1.64</u>
AdaCare	47.32 ± 0.37	83.62 ± 0.33	15.90 ± 1.16	44.41 ± 0.13	82.74 ± 0.13	15.33 ± 2.10
AdaCare-TA	47.29 ± 0.24	83.49 ± 0.23	15.45 ± 1.85	44.19 ± 0.16	82.42 ± 0.11	<u>17.78 ± 2.22</u>*
GRASP	46.96 ± 0.19	83.55 ± 0.11	13.95 ± 1.66	44.22 ± 0.31	82.87 ± 0.12	14.54 ± 0.81
GRASP-TA	46.64 ± 0.33	83.13 ± 0.18	<u>14.13 ± 1.56</u>	44.03 ± 0.41	82.49 ± 0.15	<u>16.03 ± 1.33</u>*
ConCare	46.40 ± 0.30	83.03 ± 0.10	15.30 ± 1.25	44.10 ± 0.47	82.70 ± 0.22	13.92 ± 3.04
ConCare-TA	45.91 ± 0.49	82.74 ± 0.26	15.29 ± 0.91	43.92 ± 0.46	82.48 ± 0.18	<u>15.62 ± 1.55</u>*

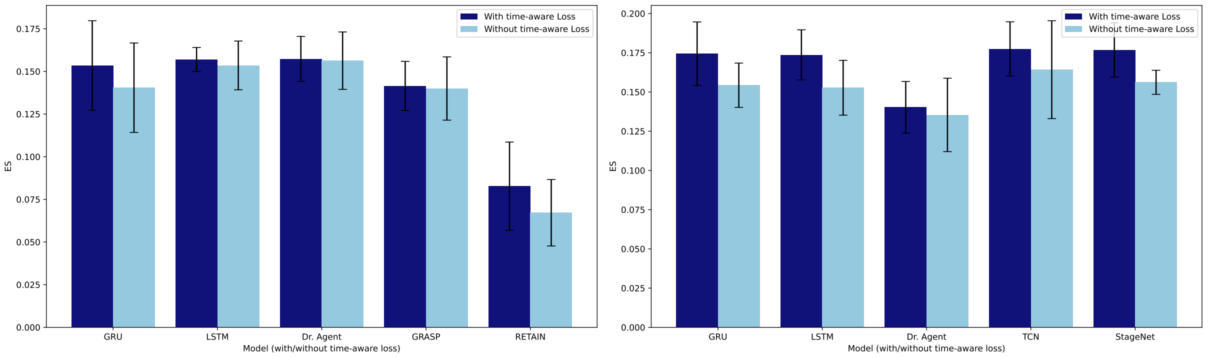

(a) MIMIC-III (b) MIMIC-IV

Figure S1: *Early prediction performance of 5 models with the highest ES on the MIMIC-III and MIMIC-IV datasets.* All models are trained using the first half of patient records. Error bars are standard deviations.

Table S9: *Statistics of the MIMIC-III dataset.* The reported statistics are of the form $Median[Q1, Q3]$.

Mortality Outcome	Total	Alive	Dead
# Patients	41517	37108 (89.38 %)	4409 (10.62 %)
# Records	3509005	2952672 (84.15 %)	556333 (15.85 %)
# Avg. records	46.0 [26.0, 88.0]	45.0 [26.0, 80.0]	66.0 [26.0, 154.0]
Age	65.5 [52.4, 77.9]	64.5 [51.6, 77.0]	74.6 [60.7, 83.3]
Age > Avg. (75.1)	12852(30.96 %)	10703(28.84 %)	2149(48.74 %)
Age $\leq$ Avg. (75.1)	28665(69.04 %)	26405(71.16 %)	2260(51.26 %)
Gender	55.9% Male	56.3% Male	52.8% Male
Male	23214(55.91 %)	20885(56.28 %)	2329(52.82 %)
Female	18303(44.09 %)	16223(43.72 %)	2080(47.18 %)
# Features		61	
Length of stay	46.9 [26.5, 88.5]	45.9 [26.3, 80.5]	68.6 [29.3, 158.8]

Table S10: *Statistics of the MIMIC-IV dataset.* The reported statistics are of the form $Median[Q1, Q3]$.

Mortality Outcome	Total	Alive	Dead
# Patients	56888	51451 (90.44 %)	5437 (9.56 %)
# Records	4055519	3466575 (85.48 %)	588944 (14.52 %)
# Avg. records	42.0 [24.0, 75.0]	41.0 [24.0, 72.0]	59.0 [24.0, 137.0]
Age	65.0 [53.0, 76.0]	64.0 [52.0, 75.0]	72.0 [60.0, 82.0]
Age > Avg. (63.1)	29950(52.65 %)	26232(50.98 %)	3718(68.38 %)
Age $\leq$ Avg. (63.1)	26938(47.35 %)	25219(49.02 %)	1719(31.62 %)
Gender	55.7% Male	55.8% Male	54.1% Male
Male	31669(55.67 %)	28726(55.83 %)	2943(54.13 %)
Female	25219(44.33 %)	22725(44.17 %)	2494(45.87 %)
# Features		61	
Length of stay	42.1 [24.0, 75.2]	41.0 [24.0, 71.9]	61.2[25.2, 138.6]

Supplemental Experimental Procedures 6
Model Embedding Visualization for Two-Stage and Multi-task Settings

To more effectively illustrate the learned embeddings from both model types, we employ t-SNE for visualizing the hidden states of patient embeddings. As depicted in Figure S2, the left figure presents the embeddings of the TCN model under a two-stage setting, while the right figure displays the embeddings from a multi-task setting. The embeddings derived under the multi-task setting appear more clustered, suggesting that the model is capable of learning more consistent embeddings in the latent space. This clustering is likely due to the model being tasked with optimizing for two objectives simultaneously.

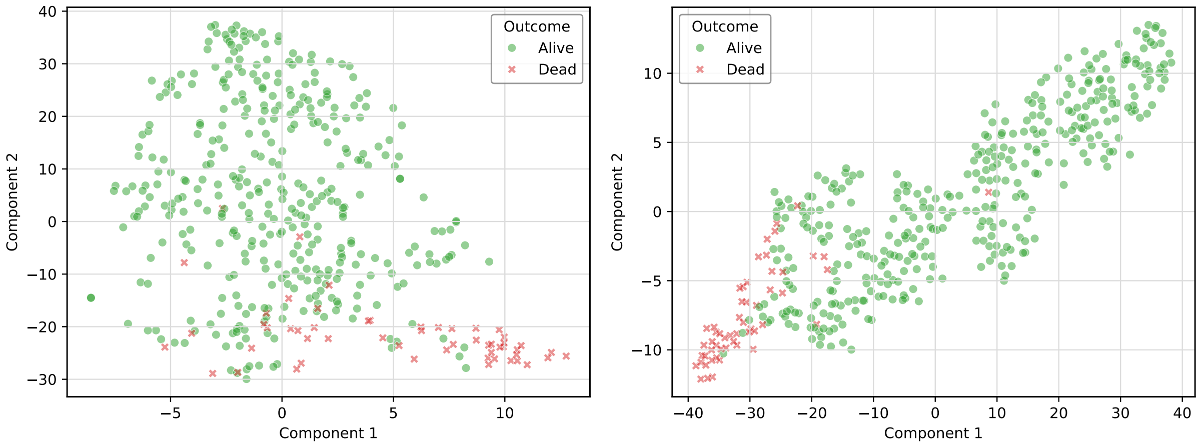

(a) TCN model's embedding under two-stage setting

(b) TCN model's embedding under multi-task setting

Figure S2: *Visualization of TCN model's embedding for LOS prediction under two settings using t-SNE.* The embeddings are extracted at each patient's last visit on the CDSL dataset.

Supplemental Experimental Procedures 7
# P-Values of T-Tests

To assess the statistical significance of our model's performance, we performed t-tests on all results. The bootstrap T-test[33] was employed to calculate p-values. We set the sample number for the bootstrap at 1000 and the number of sampling processes at 50, including all results from the 10-fold prediction process. The comparisons were made between different variations of the model — for instance, comparing the model with and without Time-Aware (TA) loss in the early mortality prediction task, and between two-stage and multi-task settings in the LOS prediction task. We report the p-value of AUPRC in the early mortality prediction task and MAE in the LOS prediction task in Table S11 and S12.

Table S11: *P-values of two-stage vs. multi-task performance in LOS predictions using t-test.* P-values are rounded to three decimal places; a value of 0.000 indicates a rounded p-value < 0.0005.

Dataset	TJH			CDSL		
Model	MAE	MSE	OSMAE	MAE	MSE	OSMAE
MLP	0.000	0.000	0.738	0.000	0.000	0.000
RNN	0.000	0.000	0.000	0.000	0.000	0.000
LSTM	0.000	0.000	0.000	0.000	0.000	0.000
GRU	0.000	0.011	0.000	0.000	0.000	0.000
TCN	0.000	0.000	0.000	0.000	0.000	0.000
Transformer	0.000	0.349	0.000	0.000	0.000	0.000
RETAIN	0.000	0.000	0.000	0.000	0.000	0.000
StageNet	0.000	0.000	0.000	0.000	0.000	0.000
Dr. Agent	0.000	0.000	0.000	0.000	0.000	0.000
AdaCare	0.000	0.000	0.000	0.000	0.000	0.000
GRASP	0.000	0.000	0.000	0.000	0.000	0.000
ConCare	0.000	0.000	0.000	0.000	0.000	0.000

Table S12: *P-values of naive models vs. models with the TA loss in early mortality predictions using t-test.* P-values are rounded to three decimal places; a value of 0.000 indicates a rounded p-value < 0.0005.

Dataset	TJH			CDSL		
Model	AUPRC	AUROC	ES	AUPRC	AUROC	ES
MLP	0.000	0.000	0.195	0.896	0.570	0.430
RNN	0.136	0.274	0.001	0.000	0.000	0.000
LSTM	0.000	0.000	0.042	1.000	0.378	1.000
GRU	0.584	0.176	0.000	0.000	0.000	0.000
TCN	0.132	0.002	0.092	0.972	0.540	0.005
Transformer	0.817	0.881	0.000	0.966	0.000	0.000
RETAIN	0.000	0.000	0.000	1.000	1.000	1.000
StageNet	0.000	0.000	0.003	0.010	0.010	1.000
Dr. Agent	0.030	0.000	0.236	0.000	0.000	0.000
AdaCare	0.586	0.118	0.000	0.700	0.964	0.007
GRASP	0.151	0.176	0.135	0.000	0.000	0.000
ConCare	0.380	0.953	0.001	0.815	0.755	1.000

Supplemental Experimental Procedures 8
Error Analysis

To more effectively analyze the sources of MAE, we plotted the MAE distributions for groups of patients who were alive and deceased. Additionally, we segmented each patient's record into two halves: the initial half and the latter half. As depicted in Figure S3, we observe that the MAE distributions for the first halves of the records are similar across both patient groups, with many records of MAE greater than 8. This is primarily because, in the initial stages of their hospital stay, patients' health statuses are often unstable, making it challenging for the model to accurately predict the remaining LOS. Conversely, during the latter half of the patients' stay, their health status tends to be more defined, resulting in a left-skewed MAE distribution for both groups. Notably, the MAE for deceased patients is predominantly less than 3, reflecting the more apparent nature of their health status and easier classification. In contrast, predicting LOS for alive patients is more complex, as their discharge from the ICU can be influenced by various external factors. Hence, their MAE values are larger (mostly clustered between 3 and 4).

These findings suggest that while the average MAE for the total cohort may be as large as the true LOS of patients, it can still effectively identify patients in critical condition, as indicated by the generally low MAE values for such cases. On the CDSL dataset, the similarity in MAE values is attributed to the fact that 87% of the patients are alive, with their MAE predominantly ranging between 2 and 4, leading to closely aligned average values.

Supplemental Experimental Procedures 9
Feature Statistics and Distributions

We provide statistics of all features which are used in the modeling process in Table S13 and S14. The data preprocessing details are shown in Figure S4. The length of stay distributions are shown in Figure S5. We plot the distributions of 16 features with the lowest missing rates in two datasets in Figure S6 and Figure S7.

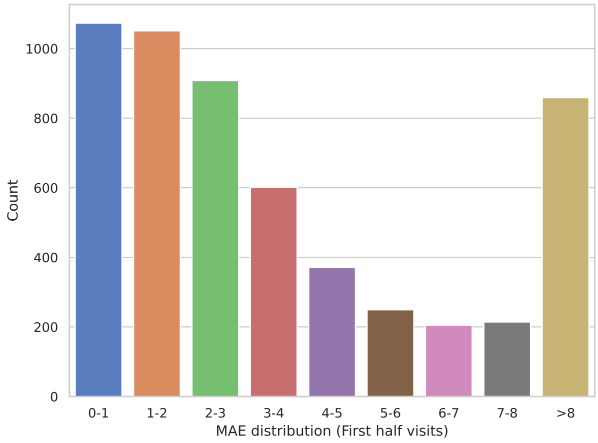

(a) The first half of records (alive patients)

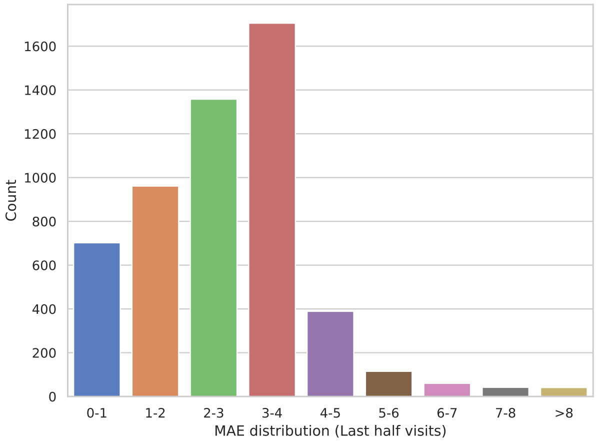

(b) The last half of records (alive patients)

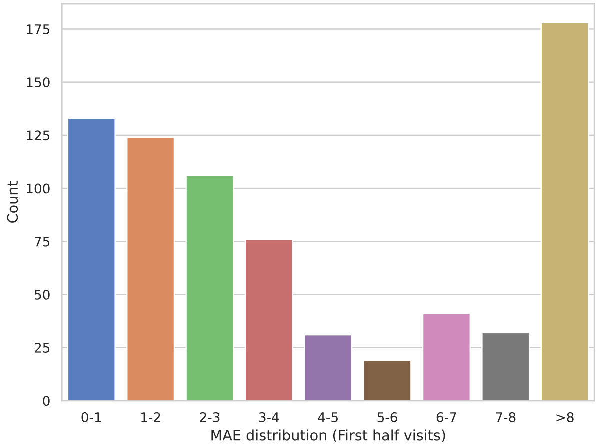

(c) The first half of records (deceased patients)

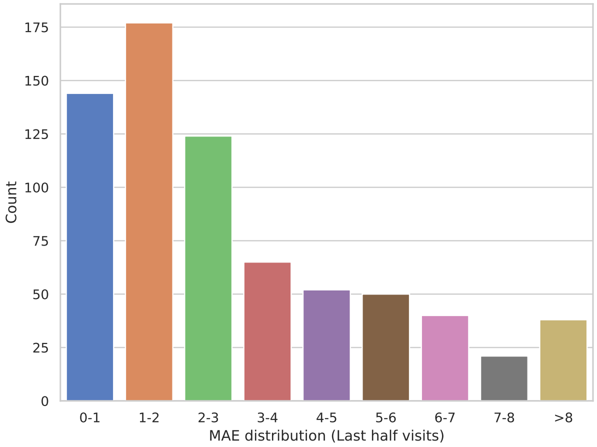

(d) The last half of records (deceased patients)

Figure S3: *MAE score distribution on the CDSL test set.* The figure compares the discrepancies in MAE performance distributions between the first and last half of patient records. The analysis utilizes the StageNet model with the multi-task setting.

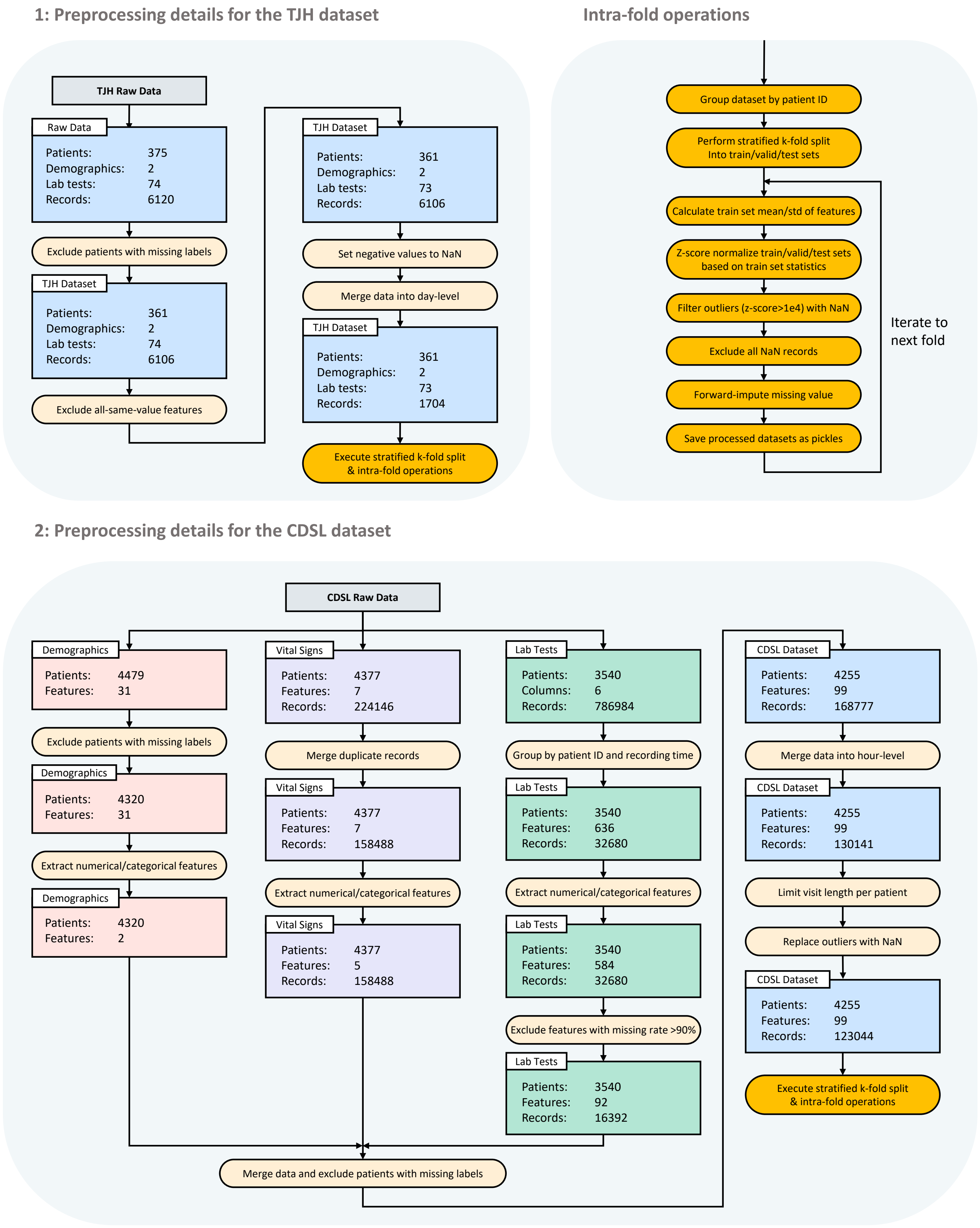

Figure S4: Data preprocessing details of two datasets.

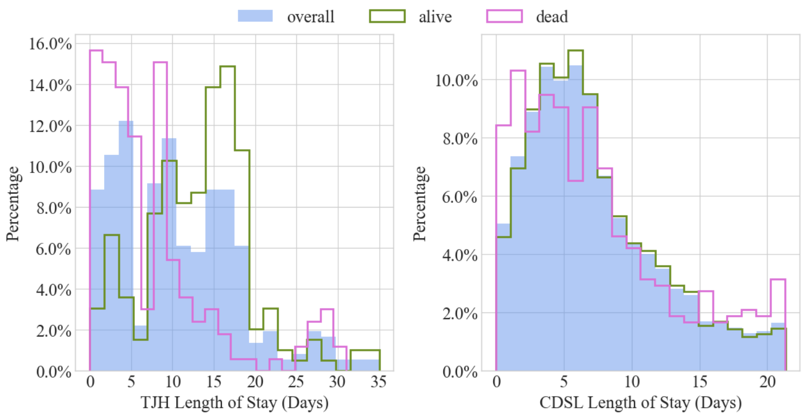

Figure S5: *Length of stay distributions in two datasets.* To keep the figure informative, we only show the statistics in the 0%-95% range for the CDSL dataset.

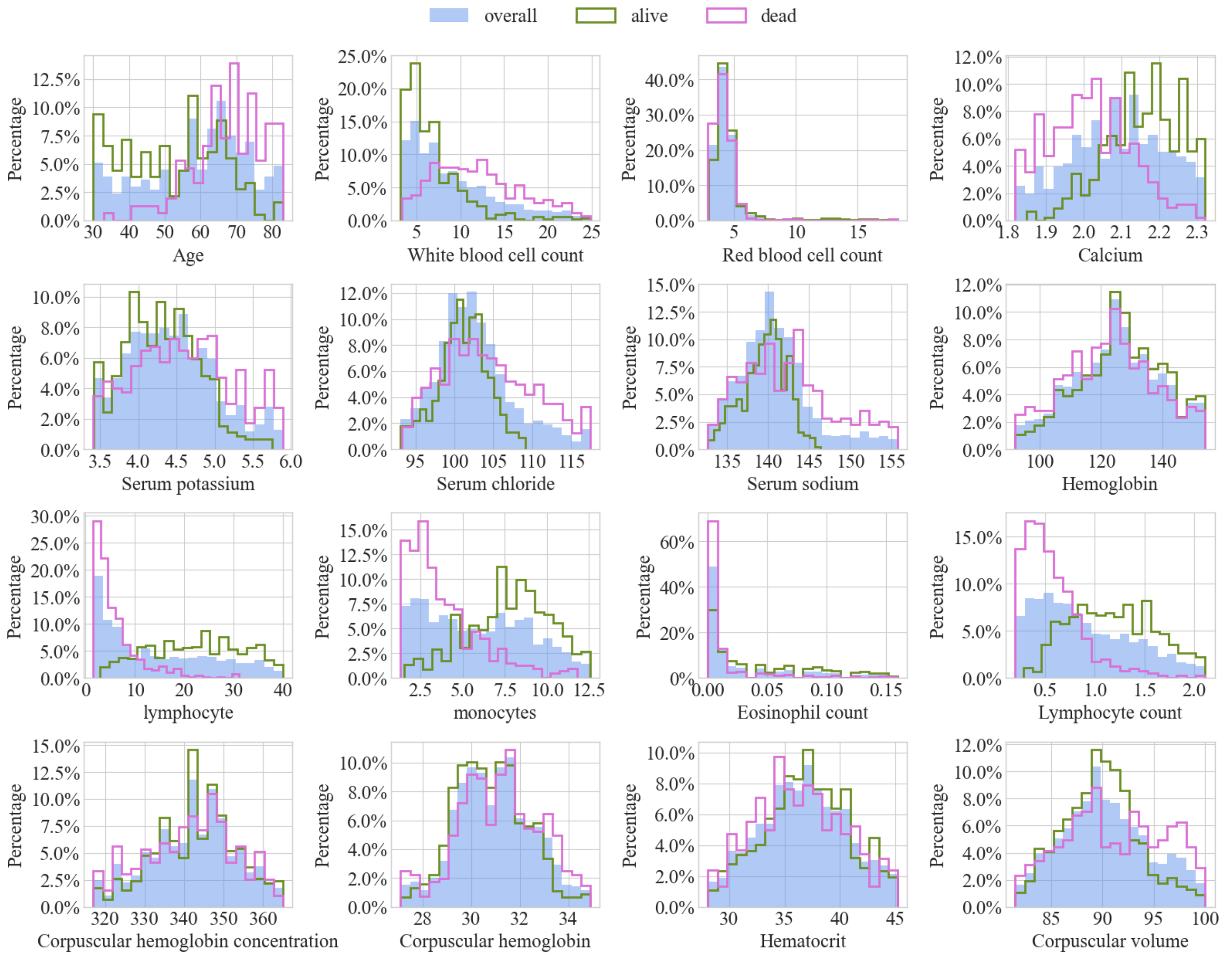

Figure S6: *Distributions of 16 features with the lowest missing rates in the TJH dataset.* We plot the data distributions of 16 features with the lowest missing rates for the overall, alive, and dead patients. The blue bars are distributions of total patients. The green and pink curves are distributions for alive and dead patients, respectively. To keep the figure informative, we only show the statistics in the 0%-95% range.

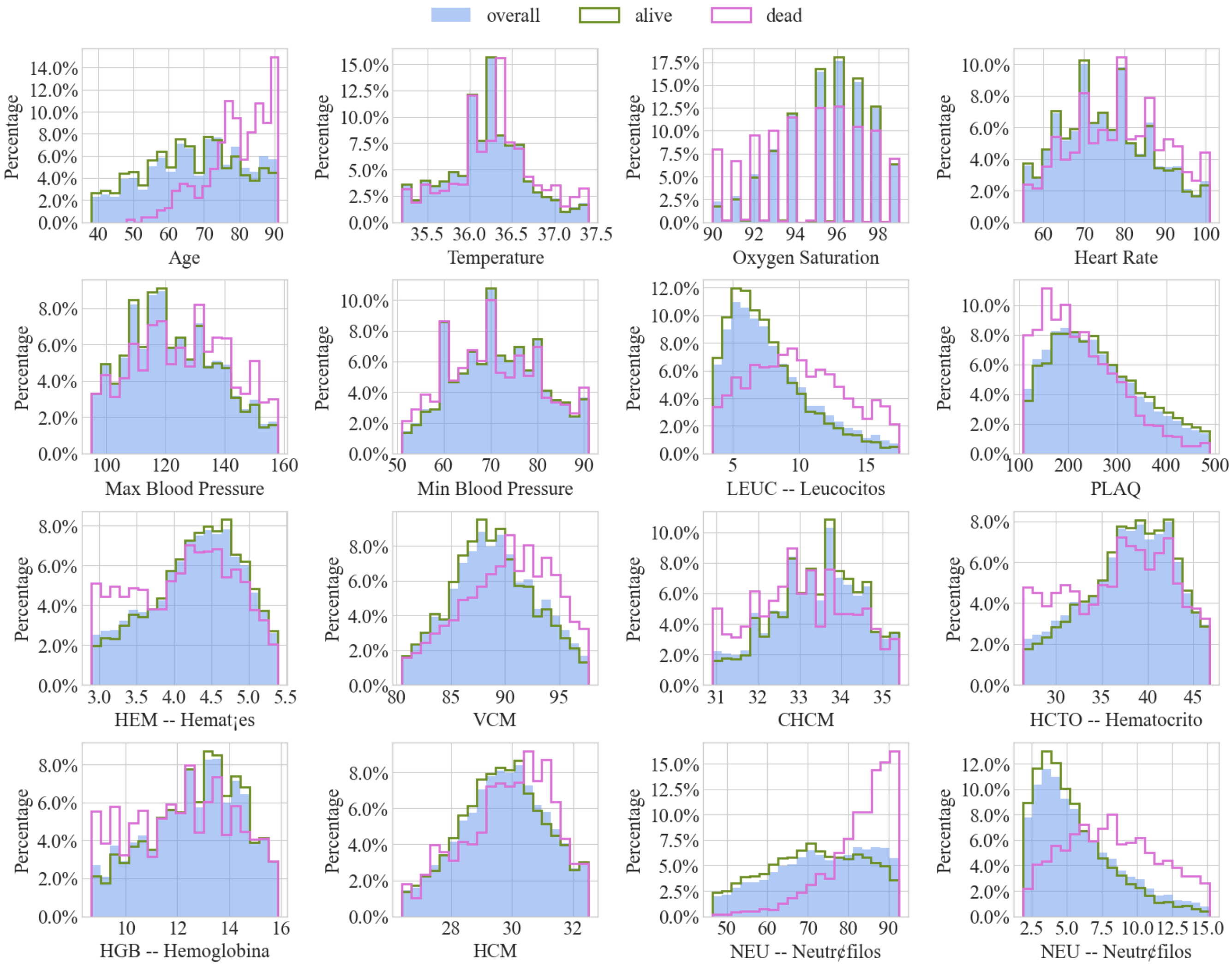

Figure S7: *Distributions of 16 features with the lowest missing rates in the CDSL dataset.* We plot the data distributions of 16 features with the lowest missing rates for the overall, alive, and dead patients. The blue bars are distributions of total patients. The green and pink curves are distributions for alive and dead patients, respectively. To keep the figure informative, we only show the statistics in the 0%-95% range.

Table S13: *Statistics of lab test features in the TJH dataset.* The reported statistics are of the form $Median[Q1, Q3]$.

Feature name	Statistics	Missing Rate
Hypersensitive cardiac troponinI	19.80 [4.20, 216.25]	29.23%
hemoglobin	125.00 [113.00, 137.00]	55.52%
Serum chloride	102.00 [98.95, 105.50]	55.58%
Prothrombin time	14.70 [13.60, 16.60]	38.44%
procalcitonin	0.10 [0.04, 0.40]	26.94%
eosinophils(%)	0.10 [0.00, 0.90]	55.40%
Interleukin 2 receptor	680.50 [460.00, 1169.75]	15.38%
Alkaline phosphatase	69.00 [54.00, 95.00]	53.81%
albumin	32.40 [27.50, 36.70]	53.99%
basophil(%)	0.20 [0.10, 0.30]	55.40%
Interleukin 10	5.80 [5.00, 12.50]	15.32%
Total bilirubin	10.60 [7.30, 16.70]	53.81%
Platelet count	181.00 [112.75, 250.25]	55.16%
monocytes(%)	5.70 [2.90, 8.70]	55.46%
antithrombin	86.00 [74.00, 97.00]	19.19%
Interleukin 8	15.95 [8.62, 33.58]	15.38%
indirect bilirubin	5.40 [3.80, 8.00]	52.46%
Red blood cell distribution width	12.60 [12.00, 13.60]	53.40%
neutrophils(%)	82.05 [65.07, 92.20]	55.40%
total protein	66.00 [61.10, 70.40]	53.81%
Treponema pallidum antibodies	0.05 [0.04, 0.07]	16.31%
Prothrombin activity	81.50 [65.00, 95.00]	38.26%
HBsAg	0.01 [0.00, 0.01]	16.31%
mean corpuscular volume	90.00 [86.90, 93.80]	55.40%
hematocrit	36.70 [33.50, 39.90]	55.40%
White blood cell count	7.60 [5.08, 12.52]	60.09%
Tumor necrosis factorα	8.60 [6.70, 11.58]	15.38%
corpuscular hemoglobin concentration	343.00 [333.75, 350.00]	55.40%
fibrinogen	4.12 [3.06, 5.51]	32.86%
Interleukin 1β	5.00 [5.00, 5.00]	15.38%
Urea	5.90 [4.00, 11.10]	53.99%
lymphocyte count	0.80 [0.47, 1.32]	55.40%
PH value	6.50 [6.00, 7.00]	21.01%
Red blood cell count	4.16 [3.67, 4.69]	60.09%
Eosinophil count	0.01 [0.00, 0.06]	55.40%
Corrected calcium	2.36 [2.27, 2.44]	52.82%
Serum potassium	4.41 [3.95, 4.86]	55.75%
glucose	6.98 [5.53, 10.15]	45.07%
neutrophils count	5.82 [3.08, 10.82]	55.40%
Direct bilirubin	4.80 [3.20, 8.00]	53.81%
Mean platelet volume	10.80 [10.10, 11.50]	50.12%
ferritin	711.60 [385.80, 1425.30]	16.49%
RBC distribution width SD	40.90 [38.50, 44.68]	53.40%
Thrombin time	16.80 [15.60, 18.30]	32.86%
lymphocyte(%)	11.70 [4.00, 25.00]	55.46%

Table S13: (Continued) Statistics of lab test features in the TJH dataset.

Feature name	Statistics	Missing Rate
HCV antibody quantification	0.06 [0.04, 0.09]	16.31%
D-D dimer	2.12 [0.60, 21.00]	36.74%
Total cholesterol	3.63 [3.01, 4.27]	53.87%
aspartate aminotransferase	27.00 [20.00, 42.00]	53.93%
Uric acid	244.00 [184.00, 332.60]	53.87%
HCO3-	23.50 [21.00, 25.90]	53.87%
calcium	2.09 [1.98, 2.19]	55.75%
NT-proBNP	571.50 [147.00, 2589.00]	27.58%
Lactate dehydrogenase	339.00 [217.00, 596.25]	53.87%
platelet large cell ratio	30.80 [25.50, 37.10]	50.12%
Interleukin 6	19.56 [4.66, 61.12]	15.61%
Fibrin degradation products	17.80 [4.00, 150.00]	19.19%
monocytes count	0.41 [0.27, 0.58]	55.40%
PLT distribution width	12.40 [11.10, 14.30]	50.12%
globulin	32.70 [29.70, 36.50]	53.81%
glutamyl transpeptidase	34.00 [22.00, 58.00]	53.81%
International standard ratio	1.14 [1.03, 1.33]	38.26%
basophil count(#)	0.01 [0.01, 0.02]	55.40%
mean corpuscular hemoglobin	30.90 [29.70, 32.20]	55.40%
Activation of partial thromboplastin time	39.20 [35.50, 44.10]	32.92%
Hypersensitive c-reactive protein	50.50 [5.35, 118.50]	42.66%
HIV antibody quantification	0.09 [0.07, 0.11]	16.26%
serum sodium	140.30 [137.70, 143.30]	55.58%
thrombocytocrit	0.21 [0.15, 0.27]	50.12%
ESR	28.00 [14.00, 45.50]	22.48%
glutamic-pyruvic transaminase	24.00 [16.00, 40.00]	53.87%
eGFR	88.10 [64.70, 104.20]	53.99%
creatinine	76.00 [58.00, 98.00]	53.99%

Table S14: *Statistics of lab test features in the CDSL dataset.* The reported statistics are of the form $Median[Q1, Q3]$.

Feature name	Statistics	Missing Rate
ADW – Coeficiente de anisocitosis	13.20 [12.10, 14.55]	8.34%
ADW – SISTEMATICO DE SANGRE	13.85 [12.30, 15.30]	2.68%
ALB – ALBUMINA	3.10 [2.70, 3.50]	1.27%
AMI – AMILASA	69.00 [46.92, 111.90]	1.25%
AP – ACTIVIDAD DE PROTROMBINA	79.00 [70.00, 87.00]	5.86%
APTT – TIEMPO DE CEFALINA (APTT)	30.80 [28.10, 33.70]	5.37%
AU – ACIDO URICO	4.70 [3.50, 6.50]	0.53%
BAS – Bas¢filos	0.02 [0.01, 0.04]	8.31%
BAS – SISTEMATICO DE SANGRE	0.02 [0.01, 0.04]	2.70%
BAS% – Bas¢filos %	0.30 [0.10, 0.50]	8.32%
BAS% – SISTEMATICO DE SANGRE	0.30 [0.10, 0.54]	2.70%
BD – BILIRRUBINA DIRECTA	0.26 [0.18, 0.40]	1.76%

Table S14: (Continued) Statistics of lab test features in the CDSL dataset.

Feature name	Statistics	Missing Rate
BE(b) – BE(b)	2.80 [0.00, 5.90]	2.57%
BE(b)V – BE (b)	1.90 [-0.60, 4.50]	1.00%
BEecf – BEecf	3.20 [-0.30, 6.78]	2.57%
BEecfV – BEecf	2.50 [-0.60, 5.40]	1.00%
BT – BILIRRUBINA TOTAL	0.40 [0.27, 0.57]	1.68%
BT – BILIRRUBINA TOTAL	0.52 [0.36, 0.77]	3.64%
CA – CALCIO	8.30 [8.00, 8.70]	0.77%
CA++ – Ca++ Gasometria	4.41 [4.21, 4.64]	2.30%
CHCM – Conc. Hemoglobina Corpuscular Media	33.40 [32.50, 34.20]	8.39%
CHCM – SISTEMATICO DE SANGRE	33.10 [32.10, 34.00]	2.70%
CK – CK (CREATINQUINASA)	66.10 [36.68, 140.00]	3.10%
CL – CLORO	102.30 [98.90, 106.43]	2.19%
CREA – CREATININA	0.82 [0.64, 1.06]	8.28%
DD – DIMERO D	985.00 [525.75, 2138.75]	6.31%
EOS – Eosin¢filos	0.03 [0.00, 0.13]	8.31%
EOS – SISTEMATICO DE SANGRE	0.10 [0.01, 0.23]	2.70%
EOS% – Eosin¢filos %	0.50 [0.00, 1.90]	8.32%
EOS% – SISTEMATICO DE SANGRE	1.30 [0.10, 3.20]	2.70%
FA – FOSFATASA ALCALINA	72.70 [55.50, 104.90]	3.29%
FER – FERRITINA	908.55 [445.92, 1645.00]	1.91%
FIB – FIBRINàGENO	562.00 [405.00, 724.00]	2.95%
FOS – FOSFORO	3.28 [2.70, 3.90]	2.15%
G-CORONAV (RT-PCR)	1.00 [0.00, 1.00]	0.49%
GGT – GGT	65.00 [30.00, 140.50]	5.53%
GLU – GLUCOSA	111.20 [94.00, 144.20]	7.67%
GOT – GOT (AST)	30.30 [20.80, 48.50]	7.42%
GPT – GPT (ALT)	32.60 [18.90, 61.00]	7.23%
HCM – Hemoglobina Corpuscular Media	29.80 [28.60, 30.80]	8.39%
HCM – SISTEMATICO DE SANGRE	29.90 [28.70, 30.90]	2.70%
HCO3 – HCO3-	27.40 [24.10, 31.10]	2.57%
HCO3V – HCO3-	27.20 [24.05, 29.90]	1.00%
HCTO – Hematocrito	38.30 [33.60, 42.00]	8.39%
HCTO – SISTEMATICO DE SANGRE	34.40 [29.60, 39.20]	2.70%
HEM – Hemat¡es	4.33 [3.75, 4.78]	8.39%
HEM – SISTEMATICO DE SANGRE	3.83 [3.25, 4.45]	2.70%
HGB – Hemoglobina	12.80 [11.10, 14.10]	8.39%
HGB – SISTEMATICO DE SANGRE	11.30 [9.70, 13.10]	2.70%
INR – INR	1.17 [1.09, 1.27]	5.87%
K – POTASIO	4.25 [3.86, 4.67]	8.10%
LAC – LACTATO	1.50 [1.10, 2.10]	2.46%
LDH – LDH	532.00 [410.05, 704.00]	7.29%
LEUC – Leucocitos	7.25 [5.34, 10.17]	8.39%
LEUC – SISTEMATICO DE SANGRE	7.70 [5.74, 10.85]	2.70%
LIN – Linfocitos	1.08 [0.72, 1.55]	8.39%
LIN – SISTEMATICO DE SANGRE	1.24 [0.79, 1.78]	2.70%
LIN% – Linfocitos %	15.50 [8.80, 24.50]	8.38%
LIN% – SISTEMATICO DE SANGRE	16.33 [9.06, 26.30]	2.70%

Table S14: (Continued) Statistics of lab test features in the CDSL dataset.

Feature name	Statistics	Missing Rate
MG – MAGNESIO	2.05 [1.81, 2.31]	2.40%
MONO – Monocitos	0.55 [0.36, 0.77]	8.32%
MONO – SISTEMATICO DE SANGRE	0.60 [0.42, 0.82]	2.70%
MONO% – Monocitos %	7.60 [4.80, 10.40]	8.32%
MONO% – SISTEMATICO DE SANGRE	7.80 [5.15, 10.30]	2.70%
NA – SODIO	138.00 [135.60, 140.70]	8.10%
NEU – Neutr¢filos	5.23 [3.46, 8.10]	8.39%
NEU – SISTEMATICO DE SANGRE	5.39 [3.53, 8.43]	2.70%
NEU% – Neutr¢filos %	74.10 [62.90, 84.60]	8.39%
NEU% – SISTEMATICO DE SANGRE	71.70 [59.20, 83.40]	2.70%
PCO2 – pCO2	41.50 [35.50, 48.50]	2.57%
PCO2V – pCO2	44.00 [38.00, 50.00]	1.00%
PCR – PROTEINA C REACTIVA	34.75 [8.72, 93.02]	8.03%
PH – pH	7.44 [7.39, 7.47]	2.57%
PHV – pH	7.40 [7.36, 7.43]	1.00%
PLAQ – Recuento de plaquetas	241.00 [176.00, 326.00]	8.39%
PLAQ – SISTEMATICO DE SANGRE	238.00 [174.00, 315.00]	2.70%
PO2 – pO2	82.80 [63.00, 106.00]	2.57%
PO2V – pO2	42.00 [27.00, 59.00]	1.00%
PROCAL – PROCALCITONINA	0.14 [0.08, 0.30]	0.77%
PT – PROTEINAS TOTALES	5.62 [5.10, 6.10]	0.84%
SO2C – sO2c (Saturaci¢n de ox¡geno)	95.67 [92.00, 97.67]	2.57%
SO2CV – sO2c (Saturaci¢n de ox¡geno)	76.00 [48.00, 90.00]	1.00%
TCO2 – tCO2(B)c	28.70 [25.20, 32.53]	2.57%
TCO2V – tCO2 (B)	28.60 [25.10, 31.49]	1.00%
TP – TIEMPO DE PROTROMBINA	13.00 [12.10, 14.10]	5.86%
TROPO – TROPONINA	15.16 [7.95, 34.92]	0.99%
U – UREA	41.60 [29.00, 61.10]	8.06%
VCM – SISTEMATICO DE SANGRE	90.00 [87.00, 93.40]	2.70%
VCM – Volumen Corpuscular Medio	88.90 [86.00, 92.30]	8.39%
VPM – SISTEMATICO DE SANGRE	10.50 [9.80, 11.30]	2.67%
VPM – Volumen plaquetar medio	10.40 [9.70, 11.10]	8.30%
VSG – VSG	47.50 [16.00, 75.00]	0.47%

References

1. Knight, S. R., Ho, A., Pius, R., Buchan, I., Carson, G., Drake, T. M., Dunning, J., Fairfield, C. J., Gamble, C., Green, C. A. et al. (2020). Risk stratification of patients admitted to hospital with covid-19 using the isaric who clinical characterisation protocol: development and validation of the 4c mortality score. bmj *370*.

2. Noh, J., Yoo, K. D., Bae, W., Lee, J. S., Kim, K., Cho, J.-H., Lee, H., Kim, D. K., Lim, C. S., Kang, S.-W. et al. (2020). Prediction of the mortality risk in peritoneal dialysis patients using machine learning models: a nation-wide prospective cohort in korea. Scientific reports *10*, 1–11.

3. Iwendi, C., Bashir, A. K., Peshkar, A., Sujatha, R., Chatterjee, J. M., Pasupuleti, S., Mishra, R., Pillai, S., and Jo, O. (2020). Covid-19 patient health prediction using boosted random forest algorithm. Frontiers in public health *8*, 357.

4. Li, S., Lin, Y., Zhu, T., Fan, M., Xu, S., Qiu, W., Chen, C., Li, L., Wang, Y., Yan, J. et al. (2021). Development and external evaluation of predictions models for mortality of covid-19 patients using machine learning method. Neural Computing and Applications (1–10).

5. Chen, T., and Guestrin, C. Xgboost: A scalable tree boosting system. In: *Proceedings of the 22nd acm sigkdd international conference on knowledge discovery and data mining* (2016):(785–794).

6. Yan, L., Zhang, H.-T., Goncalves, J., Xiao, Y., Wang, M., Guo, Y., Sun, C., Tang, X., Jing, L., Zhang, M. et al. (2020). An interpretable mortality prediction model for covid-19 patients. Nature machine intelligence *2*, 283–288.

7. Dorogush, A. V., Ershov, V., and Gulin, A. (2018). Catboost: gradient boosting with categorical features support. arXiv preprint arXiv:1810.11363. doi:`10.48550/arXiv.1810.11363`.

8. Kim, J. S. (2021). Covid-19 prediction and detection using machine learning algorithms: Catboost and linear regression. American Journal of Theoretical and Applied Statistics *10*, 208–215.

9. Tomašev, N., Glorot, X., Rae, J. W., Zielinski, M., Askham, H., Saraiva, A., Mottram, A., Meyer, C., Ravuri, S., Protsyuk, I. et al. (2019). A clinically applicable approach to continuous prediction of future acute kidney injury. Nature *572*, 116–119.

10. Rumelhart, D. E., Hinton, G. E., and Williams, R. J. (1986). Learning representations by back-propagating errors. nature *323*, 533–536.

11. Raket, L. L., Jaskolowski, J., Kinon, B. J., Brasen, J. C., Jönsson, L., Wehnert, A., and Fusar-Poli, P. (2020). Dynamic electronic health record detection (detect) of individuals at risk of a first episode of psychosis: a case-control development and validation study. The Lancet Digital Health *2*, e229—e239.

12. Choi, E., Bahadori, M. T., Schuetz, A., Stewart, W. F., and Sun, J. Doctor ai: Predicting clinical events via recurrent neural networks. In: *Machine learning for healthcare conference*. PMLR (2016):(301–318).

13. Hochreiter, S., and Schmidhuber, J. (1997). Long short-term memory. Neural computation *9*, 1735–1780.

14. Thorsen-Meyer, H.-C., Nielsen, A. B., Nielsen, A. P., Kaas-Hansen, B. S., Toft, P., Schierbeck, J., Strøm, T., Chmura, P. J., Heimann, M., Dybdahl, L. et al. (2020). Dynamic and explainable machine learning prediction of mortality in patients in the intensive care unit: a retrospective study of high-frequency data in electronic patient records. The Lancet Digital Health *2*, e179–e191.

15. Chung, J., Gulcehre, C., Cho, K., and Bengio, Y. (2014). Empirical evaluation of gated recurrent neural networks on sequence modeling. arXiv preprint arXiv:1412.3555. doi:`10.48550/arXiv.1412.3555`.

16. Meyer, A., Zverinski, D., Pfahringer, B., Kempfert, J., Kuehne, T., Sündermann, S. H., Stamm, C., Hofmann, T., Falk, V., and Eickhoff, C. (2018). Machine learning for real-time prediction of complications in critical care: a retrospective study. The Lancet Respiratory Medicine *6*, 905–914.

17. Bai, S., Kolter, J. Z., and Koltun, V. (2018). An empirical evaluation of generic convolutional and recurrent networks for sequence modeling. arXiv preprint arXiv:1803.01271. doi:`10.48550/arXiv.1803.01271`.

18. Zhang, J., Pathak, H. S., Snowdon, A., and Greiner, R. (2022). Learning models for forecasting hospital resource utilization for covid-19 patients in canada. Scientific reports *12*, 1–14.

19. Vaswani, A., Shazeer, N., Parmar, N., Uszkoreit, J., Jones, L., Gomez, A. N., Kaiser, Ł., and Polosukhin, I. (2017). Attention is all you need. Advances in neural information processing systems *30*.

20. Nitski, O., Azhie, A., Qazi-Arisar, F. A., Wang, X., Ma, S., Lilly, L., Watt, K. D., Levitsky, J., Asrani, S. K., Lee, D. S. et al. (2021). Long-term mortality risk stratification of liver transplant recipients: real-time application of deep learning algorithms on longitudinal data. The Lancet Digital Health *3*, e295–e305.

21. Choi, E., Bahadori, M. T., Sun, J., Kulas, J., Schuetz, A., and Stewart, W. (2016). Retain: An interpretable predictive model for healthcare using reverse time attention mechanism. Advances in neural information processing systems *29*.

22. Gao, J., Xiao, C., Wang, Y., Tang, W., Glass, L. M., and Sun, J. Stagenet: Stage-aware neural networks for health risk prediction. In: *Proceedings of The Web Conference 2020* (2020):(530–540).

23. Gao, J., Xiao, C., Glass, L. M., and Sun, J. (2020). Dr. agent: Clinical predictive model via mimicked second opinions. Journal of the American Medical Informatics Association *27*, 1084–1091.

24. Ma, L., Gao, J., Wang, Y., Zhang, C., Wang, J., Ruan, W., Tang, W., Gao, X., and Ma, X. (2020). Adacare: Explainable clinical health status representation learning via scale-adaptive feature extraction and recalibration. Proceedings of the AAAI Conference on Artificial Intelligence *34*, 825–832. URL: `https://ojs.aaai.org/index.php/AAAI/article/view/5427`. doi:`10.1609/aaai.v34i01.5427`.

25. Ma, L., Zhang, C., Wang, Y., Ruan, W., Wang, J., Tang, W., Ma, X., Gao, X., and Gao, J. (2020). Concare: Personalized clinical feature embedding via capturing the healthcare context. Proceedings of the AAAI Conference on Artificial Intelligence *34*, 833–840. URL: `https://ojs.aaai.org/index.php/AAAI/article/view/5428`. doi:`10.1609/aaai.v34i01.5428`.

26. Zhang, C., Gao, X., Ma, L., Wang, Y., Wang, J., and Tang, W. (2021). Grasp: Generic framework for health status representation learning based on incorporating knowledge from similar patients. Proceedings of the AAAI Conference on Artificial Intelligence *35*, 715–723. URL: https://ojs.aaai.org/index.php/AAAI/article/view/16152. doi:10.1609/aaai.v35i1.16152.

27. Feldman, M., Friedler, S. A., Moeller, J., Scheidegger, C., and Venkatasubramanian, S. Certifying and removing disparate impact. In: *proceedings of the 21th ACM SIGKDD international conference on knowledge discovery and data mining* (2015):(259–268).

28. Hardt, M., Price, E., and Srebro, N. (2016). Equality of opportunity in supervised learning. Advances in neural information processing systems *29*.

29. Calders, T., and Verwer, S. (2010). Three naive bayes approaches for discrimination-free classification. Data mining and knowledge discovery *21*, 277–292.

30. Johnson, A. E., Pollard, T. J., Shen, L., Lehman, L.-w. H., Feng, M., Ghassemi, M., Moody, B., Szolovits, P., Anthony Celi, L., and Mark, R. G. (2016). Mimic-iii, a freely accessible critical care database. Scientific data *3*, 1–9.

31. Johnson, A., Bulgarelli, L., Pollard, T., Horng, S., Celi, L. A., and Mark, R. (2020). Mimic-iv. PhysioNet. Available online at: https://physionet. org/content/mimiciv/1.0/(accessed August 23, 2021).

32. Harutyunyan, H., Khachatrian, H., Kale, D. C., Ver Steeg, G., and Galstyan, A. (2019). Multitask learning and benchmarking with clinical time series data. Scientific data *6*, 1–18.

33. Dwivedi, A. K., Mallawaarachchi, I., and Alvarado, L. A. (2017). Analysis of small sample size studies using nonparametric bootstrap test with pooled resampling method. Statistics in medicine *36*, 2187–2205.
